# Rare Chromones from a Fungal Mutant of the Marine-Derived *Penicillium purpurogenum* G59

**DOI:** 10.3390/md13085219

**Published:** 2015-08-18

**Authors:** Ming-Wen Xia, Cheng-Bin Cui, Chang-Wei Li, Chang-Jing Wu, Ji-Xing Peng, De-Hai Li

**Affiliations:** 1 State Key Laboratory of Toxicology and Medical Countermeasures, Beijing Institute of Pharmacology and Toxicology, Beijing 100850, China; E-Mails: xiamingwen@126.com (M.-W.X.); sdrlcw@126.com (C.-W.L.); wucj2009@163.com (C.-J.W.); 2 Key Laboratory of Structure-Based Drug Design & Discovery of Ministry of Education, School of Traditional Chinese Materia Medica, Shenyang Pharmaceutical University, Shenyang 110016, China; 3 Key Laboratory of Marine Drugs, School of Medicine and Pharmacy, Ocean University of China, Qingdao 266003, China; E-Mails: pengjixing1987@163.com (J.-X.P.); dehaili@ouc.edu.cn (D.-H.L.)

**Keywords:** chromone derivatives, remisporine, epiremisporine, isoconiochaetone, ECD, marine-derived fungus, *Penicillium purpurogenum* G59, DES mutagenesis

## Abstract

Three new and rare chromones, named epiremisporine B (**2**), epiremisporine B1 (**3**) and isoconiochaetone C (**4**), along with three known remisporine B (**1**), coniochaetone A (**5**) and methyl 8-hydroxy-6-methyl-9-oxo-9*H*-xanthene-1-carboxylate (**6**) were isolated from a mutant from the diethyl sulfate (DES) mutagenesis of a marine-derived *Penicillium purpurogenum* G59. The structures of **2**–**4** including the absolute configurations were determined by spectroscopic methods, especially by NMR analysis and electronic circular dichroism (ECD) experiments in conjunction with calculations. The absolute configuration of the known remisporine B (**1**) was determined for the first time. Compounds **2** and **3** have a rare feature that has only been reported in one example so far. The compounds **1**–**6** were evaluated for their cytotoxicity against several human cancer cell lines. The present work explored the great potential of our previous DES mutagenesis strategy for activating silent fungal pathways, which has accelerated the discovery of new bioactive compounds.

## 1. Introduction

Cyclopentachromones (CPCs), the tricyclic cyclopentabenzopyan-9-one derivatives, are relatively rare in nature. Only a few in this family has so far been reported from fungi, including coniochaetones A–D [1,2,3] and E–I [4], coniothyrione [5,6], diaportheones A–B [7], preussochromones D–F [8], cryptosporioptide [9], and remisporine A [10]. Remisporine B is a unique CPC dimer resulting from a spontaneous Diels-Alder reaction of remisporine A [10].

Activating silent pathways for secondary metabolites in fungi has been a promising route to discover new compounds [11,12,13,14]. Several strategies, such as the one strain-many compounds [15], co-cultivation [16], and chemical epigenetics [17] strategies, have been widely applied for this purpose. The ribosome engineering [18,19] strategy, awaking silent bacterial pathways by introducing drug-resistant mutations to obtain new compounds [20,21], has been well studied in bacteria. Recently, this strategy has been applied to fungi due to the development of new methodologies particularly for fungi [22,23,24,25,26,27]. The recently reported mutagenesis strategy using diethyl sulphate (DES) also provided a practical method to discover new compounds by activating silent fungal pathways [28,29,30,31].

**Figure 1:**
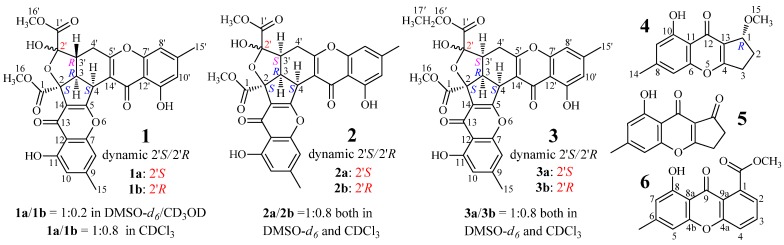
Structures of **1**–**6** produced in the mutant AD-1-2 of *P. purpurgenum* G59.

AD-1-2 is a mutant strain from the DES mutagenesis [28] of a marine-derived fungus, *Penicillium* *purpurogenum* G59 [32]. Previously, we reported three new unusual C25 steroids from AD-1-2 [30]. Here, we report three new chromone derivatives, epiremisporine B (**2**), epiremisporine B1 (**3**) and isoconiochaetone C (**4**), together with the three known ones, remisporine B [10] (**1**), coniochaetone A (**5**) [1,2] and methyl 8-hydroxy-6-methyl-9-oxo-9*H*-xanthene-1-carboxylate (**6**) [33] (Figure 1), from the same mutant AD-1-2. All of the six compounds were not presented in the parent G59 extract. The absolute configuration of **1** was determined by electronic circular dichroism (ECD) experiments in conjunction with calculations, for the first time in the present study. Compounds **2** and **3** are new CPC dimers with a rare feature that has only been reported in one case (**1**) as far as we know.

## 2. Results and Discussion

### 2.1. Identification and Absolute Configuration Determination of Remisporine B (**1**)

**Figure 2:**
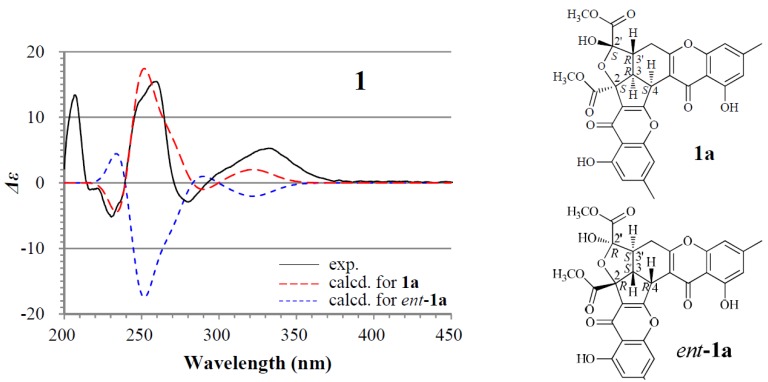
Experimentally measured and calculated electronic circular dichroism (ECD) spectra of **1** in MeOH.

Compound **1**, a yellow amorphous powder (MeOH), ${\left[\alpha \right]}_{D}^{25}$ +898.9 (*c* 0.18, MeOH), gave a protonated molecular ion peak at *m*/*z* 577 [M + H]^+^ in positive ESIMS and a deprotonated one at *m*/*z* 575 [M − H]^−^ in negative mode. The UV absorptions of **1** at 227.2, 240.5, 259.0, and 323.3 nm showed the feature of the chromone skeleton. The ^1^H and ^13^C NMR spectra of **1** in DMSO-*d*_6_ showed signals arising from nearly a single major isomer (Table 1), which were confirmed to be identical with those of remisporine B [10]. Eventually, **1** was identified as remisporine B by the NOEs detected between H-4/H-3, H-3/Hα-4′, Hα-4′/H-3′, and H-3′/HO-2′ by the 1D ROESY experiments in DMSO-*d*_6_ and the CD spectrum (Figure 2) which is identical to that of remisporine B [10]. The ^1^H NMR spectrum of **1** in CDCl_3_ showed two sets of ^1^H signals from the isomers **1a** and **1b** in 1:0.8 ratio (Table 2), which were analyzed by comparison with the data in DMSO-*d*_6_ (Table 1). Significant differences in the ^1^H NMR data (Table 2) between **1a** and **1b** appeared mainly on the protons around C-2′ on the aliphatic rings, H-3, H-3′, H-4, H-4′, and HO-2′, indicating that they are epimers at C-2′. Careful examination of the ^1^H NMR spectrum of **1** in DMSO-*d*_6_ (in the Supplementary Information) has shown that except for the major set of ^1^H signals from **1a** (Table 1), additional very weak signals from H-4, HO-2′, HO-11, and H-11′ of the minor isomer **1b** were also clearly recognized in the spectrum, although the other proton signals for **1b** were hardly assigned because of the signal broadening or overlapping. The ratio of **1a** to **1b** was determined approximately to be 1:0.2 by the standard integrals of related proton signals. Similarly, in CD_3_OD, **1** also existed as a pair of **1a** and **1b** in the same 1:0.2 ratio. These observations indicated that **1** exists in a dynamic isomerism between **1a** and **1b** in the solutions.

For the major isomer **1a** which should dominate the CD curve of **1**, there are two possible absolute configurations, 2*S*3*R*4*S*2′*S*3′*R* (**1a**) and its enantiomer 2*R*3*S*4*R*2′*R*3′*S* (*ent*-**1a**). We performed ECD calculations on **1a** and *ent*-**1a**. Time dependent density functional theory (TDDFT) ECD calculations performed at the B3LYP/6-31+G(d) level were used to generate ECD spectra for a set of five lowest-energy conformers for each of **1a** and *ent*-**1a**. The resulting ECD spectra were combined by Boltzmann weighting to give a composite spectrum. By contrast with the enantiomer *ent*-**1a**, the calculated ECD spectrum of the enantiomer **1a** matched well the experimental data of **1** (Figure 2). This thus enabled us to assign the 2*S*3*R*4*S*2′*S*3′*R* absolute configuration to the major isomer **1a**. Accordingly, the minor isomer **1b** was reasonably assigned the 2*S*3*R*4*S*2′*R*3′*R* absolute configuration.

**Table 1:** The ^1^H and ^13^C NMR data of **1**–**3** in DMSO-*d*_6_. ^a^

Position	1a ^b^		2a ^c^		2b ^c^		3a ^c^		3b ^c^	
	δ_H_ (*J* in Hz)	δ_C_	δ_H_ (*J* in Hz)	δ_C_	δ_H_ (*J* in Hz)	δ_C_	δ_H_ (*J* in Hz)	δ_C_	δ_H_ (*J* in Hz)	δ_C_
1	—	170.0 s	—	170.8, s	—	171.3 s	—	170.8 s	—	171.4 s
2	—	86.6 s	—	88.2 s	—	89.5 s	—	88.2 s	—	89.4 s
3	3.29 (dd, 12.2, 6.0)	47.2 d	3.78 (dd, 9.2, 9.0)	47.0 d	3.88 (dd, 9.0, 8.4)	46.6 d	3.81 (dd, 9.4, 8.9)	47.3 d	3.87 (dd, 9.0, 8.4)	46.6 d
4	4.60 (d, 6.0)	39.1 d	4.98 (d, 9.0)	36.9 d	5.02 (d, 9.0)	36.2 d	4.99 (d, 8.9)	36.9 d	5.01 (d, 9.0)	36.2 d
5	—	171.5 s	—	169.3 s	—	168.9 s	—	168.9 s	—	168.9 s
7	—	156.7 s	—	156.78 s	—	156.75 s	—	156.8 s	—	156.7 s
8	6.86 (s)	108.3 d	6.79 (s)	108.3 d	6.77 (s)	108.3 d	6.81 (s)	108.3 d	6.79 (s)	108.3 d
9	—	147.5 s	—	147.5 s	—	147.5 s	—	147.47 s	—	147.51 s
10	6.69 (s)	112.5 d	6.64 (s)	112.3 d	6.64 (s)	112.3 d	6.65 (s)	112.3 d	6.65 (s)	112.3 d
11	—	159.8 s	—	159.87 s	—	159.85 s	—	159.9 s	—	159.8 s
12	—	108.5 s	—	108.4 s	—	108.4 s	—	108.4 s	—	108.4 s
13	—	179.7 s	—	178.87 s	—	178.89 s	—	178.8 s	—	178.9 s
14	—	119.3 s	—	119.2 s	—	118.7 s	—	119.3 s	—	118.7 s
15	2.35 (s)	21.5 q	2.30 (s)	21.5 q	2.28 (s)	21.4 q	2.30 (s)	21.45 q	2.29 (s)	21.42 q
16	3.71 (s)	52.7 q	3.69 (s)	52.8 q	3.70 (s)	52.6 q	3.68 (s)	52.6 q	3.70 (s)	52.5 q
1′	—	168.1 s	—	169.4 s	—	167.7 s	—	169.2, s	—	167.4 s
2′	—	102.8 s	—	105.7 s	—	106.2 s	—	105.7 s	—	106.1 s
3′	2.63 (tdd, 12.2, 4.3, 1.3)	45.5 d	3.10 (ddd, 10.1, 9.2, 6.4)	42.7 d	2.79 (ddd, 12.5, 8.4, 5.9)	47.3 d	3.09 (ddd, 10.3, 9.4, 6.5)	42.9 d	2.79 (ddd, 11.3, 8.4, 6.6)	47.2 d
4′α	2.91 (dd, 16.8, 12.2)	28.4 t	2.70 (dd, 17.0, 6.4)	26.3 t	2.48 (dd, 15.9, 5.9)	26.9 t	2.69 (dd, 16.9, 6.5)	26.3 t	2.48 (dd, 15.9, 6.6)	27.0 t
β	2.83 (dd, 16.8, 4.3)		2.64 (dd, 17.0, 10.1)		2.43 (dd, 15.9, 12.4)		2.64 (dd, 16.9, 10.3)		2.44 (dd, 15.9, 11.3)	
5′	—	169.0 s	—	168.0 s	—	167.4 s	—	168.1 s	—	167.1 s
7′	—	155.5 s	—	155.5 s	—	155.6 s	—	155.5 s	—	155.6 s
8*′*	6.91 (s)	107.4 d	6.90 (s)	107.6 d	6.88 (s)	107.6 d	6.92 (s)	107.6 d	6.91 (s)	107. 7 d
9′	—	147.7 s	—	147.40 s	—	147.44 s	—	147.40 s	—	147.43 s
10*′*	6.72 (s)	111.8 d	6.71 (s)	112.0 d	6.71 (s)	112.0 d	6.72 (s)	111.9 d	6.72 (s)	111.9 d
11′	—	159.4 s	—	159.52 s	—	159.55 s	—	159.5 s	—	159.5 s
12′	—	107.3 s	—	107.7 s	—	107.8 s	—	107.8 s	—	107.7 s
13′	—	181.4 s	—	179.4 s	—	179.3 s	—	179.4 s	—	179.3 s
14′	—	113.1 s	—	111.9 s	—	111.7 s	—	111.9 s	—	111.8 s
15′	2.39 (s)	21.8 q	2.38 (s)	21.77 q	2.38 (s)	21.79 q	2.39 (s)	21.8 q	2.38 (s)	21.8 q
16′	3.67 (s)	52.4 q	3.74 (s)	52.28 q	3.75 (s)	52.31 q	4.25–4.18 (m)	61.3 t	4.17–4.12 (m)	61.2 t
17′	—	—	—	—	—	—	1.26 (t, 7.1)	13.8 q	1.26 (t, 7.1)	14.0 q
11–OH	12.20 (s)	—	12.15 (s)	—	12.12 (s)	—	12.16 (s)	—	12.13 (s)	—
2′–OH	7.96 (d, 1.3)	—	7.80 (s)	—	7.53 (s)	—	7.74 (s)	—	7.49 (s)	—
11′–OH	12.50 (s)	—	12.49 (s)	—	12.47 (s)	—	12.50 (s)	—	12.48 (s)	—

^a^ The ^1^H and ^13^C NMR signals were recorded in *δ* values using the solvent DMSO-*d*_6_ signals (δ_H_ 2.50/δ_C_ 39.52) as references, respectively. ^b^ Recorded at 400 MHz ^1^H and 150 MHz ^13^C NMR. Except for the ^1^H signals of **1a** given in this Table, additional very weak ^1^H signals of minor isomer **1b** (δ4.74, d, *J* = 8.5 Hz, H-4; δ12.28, br s, HO-11; δ7.83, br s, HO-2′; δ12.53, br s, HO-11′) were also detected in the ^1^H NMR spectrum of **1** although full ^1^H signals were hardly recognized for **1b**. The ratio of **1a** and **1b** was determined approximately to be 1:0.2 by the standard integrals of their H-4 and HO-2′ signals. ^c^ Recorded at 600 MHz ^1^H and 150 MHz ^13^C NMR. The signals of **2a** and **2b** for **2** were assigned on the basis of HMQC, HMBC, NOESY, and 1D GOESY experiments. The signals of **3a** and **3b** for **3** were assigned by comparison with the data of **2a** and **2b**, coupled with the result of NOESY experiments for **3**. The ratio of **2a**/**2b** and **3a**/**3b** were approximately determined both to be 1:0.8 by the standard integrals of H-4, HO-2′ and H-3′ signals for **2** and H-3 and H-3′ signals for **3**, respectively.

**Table 2:** The 400 MHz ^1^H and 100 MHz ^13^C NMR data of **1** and **4** in CDCl_3_. ^a^

Proton	1		Position	4	
	δ_H_ (*J* in Hz) of 1a	δ_H_ (*J* in Hz) of 1b		δ_H_ (*J* in Hz)	δ_C_
3	3.25 (dd, 12.0, 6.0)	3.54 (dd, 11.3, 7.0)	1	4.94 (dt, 6.8, 1.5)	79.5 d
4	4.78 (d, 6.0)	4.98 (d, 7.0)	2α	2.14 (dddd, 14.0, 8.6, 2.6, 1.5)	27.8 t
8	6.71 (s)	6.70 (s)	β	2.31 (dddd, 14.0, 9.4, 7.4, 6.8)	
10	6.63 (s)	6.63 (s)	3α	3.17 (dddd, 18.0, 8.6, 7.4, 1.5)	30.3 t
15	2.38 (s)	2.37 (s)	β	2.77 (ddd, 18.0, 9.4, 2.6)	
16	3.82 (s)	3.90 (s)	4	—	174.0 s
3′	2.78 (ddd, 12.7, 12.0, 4.0)	3.93 (td, 11.3, 5.1)	6	—	157.5 s
4′α	2.94 (dd, 17.0, 12.7)	2.75 (dd, 17.6, 11.9)	7	6.70 (br s)	107.8 d
β	2.84 (dd, 17.0, 4.0)	3.05 (dd, 17.6, 5.1)	8	—	146.8 s
8′	6.78 (s)	6.73 (s)	9	6.63 (br s)	112.8 d
10′	6.71 (s)	6.70 (s)	10	—	161.2 d
15′	2.43 (s)	2.43 (s)	11	—	109.2 d
16′	3.80 (s)	3.85 (s)	12	—	181.3 d
11–OH	12.12 (s)	11.89 (s)	13	—	120.1 d
2′–OH	4.60 (br s)	4.49 (s)	14	2.39 (s)	22.4 q
11′–OH	12.37 (s)	12.34 (s)	15	3.49 (s)	57.5 q
—	—	—	10-OH	12.55 (s)	—

^a^ ^1^H and ^13^C NMR signals were recorded as δ values using the solvent CDCl_3_ signals (δ_H_ 7.26 and δ_C_ 77.16) as references, respectively. Remisporine B (**1**) existed in dynamic isomerism between major isomer **1a** and minor isomer **2b** in CDCl_3_, and the signals of **1a** and **1b** were assigned by comparison with the data of **1a** in DMSO, given in Table 1. The ratio of **1a** and **1b** was determined approximately to be 1:0.8 by the standard integrals of their H-4 signals.

### 2.2. Structure Determination of New Chromones **2**–**4**

Epiremisporine B (**2**) had the molecular formula C_30_H_24_O_12_ by HRESIMS, the same as **1**, and the IR absorptions indicated the ester (1742 cm^−1^) and conjugated (1655 cm^−1^) carbonyls in **2**. The similar UV and 1D NMR data (Table 1) of **2** and **1** indicated their similar structures. Differing from **1**, however, **2** gave both ^1^H and ^13^C NMR signals (Table 1) as pairs in a ratio of 1:0.8 in DMSO-*d*_6_, indicating that **2** is a stereoisomer of **1** and exists in a dynamic isomerism between major (**2a**) and minor (**2b**) isomers in DMSO-*d*_6_. The planar structure of **2**, the same as **1**, was deduced by 1D (Table 1) and 2D NMR data (Table S1). Finally, **2** was determined to be an epimer of **1** at C-3′ by the 1D GOESY experiments. The orientation of H-3′, H-3 and H-4 in the same spatial direction on the same side of the ring system in **2** was established by the NOEs detected between H-3′/H-3, H-3′/H-4 and H-3/H-4 of **2a** and **2b** by the 1D GOESY experiments. Further in the 1D GOESY experiments, a more remarkable NOE was detected on 2′-OH in **2b** than in **2a** by irradiating H-3′ in **2a** and **2b**, respectively. This thus evidenced that **2b** is an isomer with *cis* H-3′/2′-OH and **2a** is another one with *trans* H-3′/2′-OH.

Epiremisporine B1 (**3**) was assigned the molecular formula C_31_H_26_O_12_ by HRESIMS, which had one more CH_2_ than **2**. It showed UV and IR absorptions similar to **2**, and the ^1^H and ^13^C NMR spectra of **3** in DMSO-*d*_6_ and CDCl_3_ gave both pairs of signals in a ratio of 1:0.8, indicating the presence of dynamic isomerism in the solutions between major **3a** and minor **3b** isomers, like **2**. The ^1^H and ^13^C NMR data of **3a** and **3b** in DMSO-*d*_6_ (Table 1) and CDCl_3_ (Table 3) are almost identical with those of **2a** and **2b** in DMSO-*d*_6_ (Table 1) and CDCl_3_ (Table 3), respectively, except for the signals from an ethoxy group in **3a** and **3b** instead of the signals of the 16′ methoxy group in **2a** and **2b**. Thus, **3** must be a derivative of **2**, with an ethoxy group instead of the 16′ methoxy group in **2**. The NOEs detected between H-4/H-3′, H-3′/H-3 and H-3/H-4 in the NOESY spectrum of **3** in DMSO-*d*_6_, especially the NOE between H-4/H-3′, further evidenced the relative stereochemistry of **3**, the same as **2**.

**Table 3:** The 400 MHz ^1^H NMR data (δ, *J* in Hz) of **2** and **3** in CDCl_3_. ^a^

Proton	2		3	
	2a	2b	3a	3b
3	3.93 (t, 9.0)	3.86–3.82 (masked by H-16/16′)	3.95 (t, 9.0)	3.90 (t, 9.0)
4	5.19 (d, 9.0)	5.22 (d, 9.0)	5.21 (d, 9.0)	5.22 (d, 9.0)
8	6.70 (s)	6.70 (s)	6.70 (s)	6.70 (s)
10	6.60 (s)	6.60 (s)	6.60 (s)	6.60 (s)
15	2.32 (s)	2.30 (s)	2.33 (s)	2.32 (s)
16	3.79 (s)	3.82 (s)	3.79 (s)	3.84 (s)
3′	3.01 (ddd, 11.7, 9.0, 5.8)	2.93 (ddd, 12.8, 8.2, 5.2)	2.98 (ddd, 11.9, 9.0, 5.7)	2.92 (ddd, 12.7, 9.0, 5.4)
4′α	2.87 (dd, 16.7, 5.8)	2.81 (dd, 15.9, 5.2)	2.88 (dd, 16.6, 5.7)	2.81 (dd,15.9, 5.4)
β	2.62 (dd, 16.7, 11.7)	2.46 (dd, 15.9, 12.8)	2.62 (dd, 16.6, 11.9)	2.49 (dd,15.9, 12.7)
8′	6.71 (s)	6.70 (s)	6.72 (s)	6.71 (s)
10′	6.68 (s)	6.68 (s)	6.69 (s)	6.69 (s)
15′	2.42 (s)	2.42 (s)	2.42 (s)	2.42 (s)
16′	3.87 (s)	3.85 (s)	4.37-4.29 (m)	4.35-4.27 (m)
17′	—	—	1.35 (t, 7.1)	1.37 (t, 7.0)
11–OH	12.04 (s)	11.95 (s)	12.06 (s)	11.98 (s)
2′–OH	4.64 (br s)	4.64 (br s)	4.64 (br s)	4.64 (br s)
11′–OH	12.35 (s)	12.30 (s)	12.36 (s)	12.31 (s)

^a^ The chemical shift was recorded as δ value using the solvent CDCl_3_ signal (δ_H_ 7.26) as reference. Both **2** and **3** existed in dynamic isomerism between the major and minor isomers, **2a**/**2b** and **3a**/**3b**, in CDCl_3_, respectively. The signals for each isomer were assigned by comparison with their data in DMSO given Table 1, respectively. The ratio of **2a**/**2b** and **3a**/**3b** were approximately determined both to be 1:0.8 by the standard integrals of H-4, HO-11 and HO-11′ signals for **2** and Hβ-4 signals for **3**, respectively.

**Figure 3:**
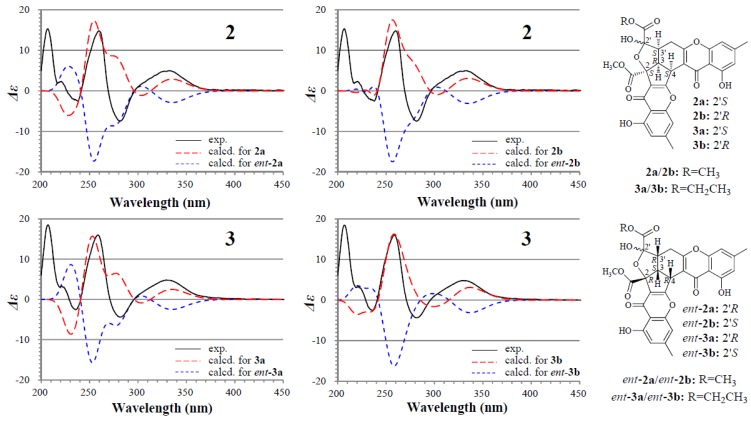
Experimentally measured and calculated ECD spectra of **2** and **3** in MeOH.

The absolute configuration of **2** and **3** was determined on the basis of experimental and theoretically ECD data. Because **2a**/**2b** and **3a**/**3b** exist in nearly equal proportion (1:0.8) in the solutions of **2** and **3**, there are four questionable absolute configurations 2*S*3*R*4*S*2′*S*3′*S* (**2a**/**3a**) and 2*S*3*R*4*S*2′*R*3′*S* (**2b**/**3b**) and their enantiomers 2*R*3*S*4*R*2′*R*3′*R* (*ent*-**2a**/*ent*-**3a**) and 2*R*3*S*4*R*2′*S*3′*R* (*ent*-**2b**/*ent*-**3b**) for **2** and **3**. We performed theoretical ECD calculations on all four questionable absolute configurations for **2** and **3** because it was unknown which one of them mainly dominates the CD of **2** and **3**, although the chirality of C-2′ would little affect the CD in view of its location far away from the aromatic ring systems. TDDFT calculations performed at the B3LYP/6-31+G(d) level for **2** and the B3LYP/6-31G(d) level for **3** were used to generate ECD spectra for a set of lowest-energy conformers for each absolute configuration, and the resulting spectra were combined by Boltzmann weighting to give a composite spectrum. The calculated ECD spectra of the enantiomers **2a** and **2b** for **2** and **3a** and **3b** for **3** properly reproduced the experimental data. Especially the calculated ECD spectra of the enantiomers **2b** and **3b** agreed well with the experimental data of **2** and **3** (Figure 3), indicating that the enantiomers **2b** and **3b** likely dominated the CD of **2** and **3**, respectively. Thus, the 2*S*3*R*4*S*2′*S*3′*S* and 2*S*3*R*4*S*2′*R*3′*S* absolute configurations were assigned to the isomers **2a**/**3a** and **2b**/**3b**, respectively.

Isoconiochaetone C (**4**), colorless needles (MeOH), m.p. 99–100 °C, ${\left[\alpha \right]}_{D}^{25}$ +76.7 (*c* 0.16, MeOH), was assigned the molecular formula C_14_H_14_O_4_ by HRESIMS. The molecular formula and the typical UV absorptions indicated that **4** is a monomeric chromone. The ^1^H and ^13^C NMR data of **4** in CDCl_3_ (Table 2) matched well the data of coniochaetone C in CDCl_3_ [3] although splitting patters of H-1 and H_2_-2 signals in the literature [3] could not be fully analyzed probably because of the limitation in the spectral resolution, indicating the same planar structure of both compounds. Coniochaetone C, with the 1*S* absolute configuration, showed negative optical rotation (${\left[\alpha \right]}_{D}^{23}$ −49.0 (*c* 0.1, MeOH) [3], while **4** gave a positive sign (+76.7) which fitted well with the optical rotation of their 1*R*-OH analogue coniochaetone B (${\left[\alpha \right]}_{D}^{25}$ +84.0; *c* 0.1, MeOH) [1], indicating that **4** has the 1*R* absolute configuration.

### 2.3. Detection of **1**–**6** in the Mutant AD-1-2 Extract by HPLC-PDAD-UV/HPLC-ESI-MS Analyses

Each of the EtOAc extracts of mutant AD-1-2 and the parent G59 strain was analyzed by HPLC-photodiode array detector (PDAD)-UV and HPLC-electron spray ionization (ESI)-MS. In the HPLC-PDAD-UV analysis, **1**–**6** were detected in the mutant but not in the G59 extract, which was confirmed by both retention times and UV spectra in comparison to those of standard compounds **1**–**6** (Figure S1). In the HPLC-PDAD-UV profile, compound **3** was only detected in a trace at 242 nm (Figure S1), by contrast, it was clearly detected by HPLC-SEI-MS. The other five compounds were also detected in the mutant extract by selective *pseudo*-molecular ion monitoring with both extracted ion chromatograms and MS spectra, but not in the G59 extract (Figure S2).

### 2.4. Mechanism of **1**–**3** Formation

**Figure 4:**
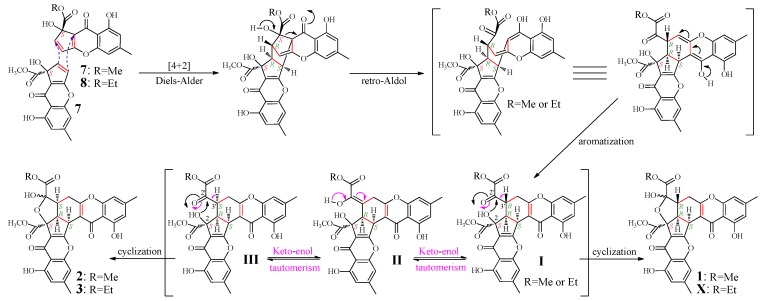
Proposed mechanism for the **1**–**3** formation from **7** and **8**, extending the original proposal of Kong *et al*. for the **1** formation from **7**, initially reported in the literature [10]. Me and Et are the abbreviation of methyl and ethyl respectively.

According to the mechanism of **1** formation from remisporine A (**7**) [10], **2** and **3** were expected to be formed by dimerization of **7** and **8** (Figure 4). Indeed, both **7** and **8** were presumably detected in the mutant but not the G59 extract in the HPLC-PDAD-UV and HPLC-ESI-MS analyses (Figures S1 and S2). However, **7** and **8** could not be obtained in pure form because of their instability [10]. Thus, we proposed the mechanism for the **1**–**3** formation, as shown in Figure 4, which involved the formation of C-3′ isomers, extending the original proposal of Kong *et al.* [10]. The spontaneous Diels-Alder reaction of two molecules of **7** or of combination of **7** and **8** followed by the *retro*-Aldol condensation and aromatization could produce **I**. The intermediate **I** would further undergo keto-enol tautomerism with **II** as an intermediate to give **III** bearing an inverted absolute configuration of C-3′. The intramolecular cyclization of **I** *via* attacking the keto carbonyl at C-2′ by the hydroxyl at C-2 could produce **1** and **X**, existing as interconvertible isomers in solutions. A similar process could be applied to **III** to generate **2** and **3**. Incidentally, according to the mechanism that requires the *endo* mode of the Diels-Alder reaction of **7** and **8**, adopting the less hindered orientation with hydroxyl groups facing each other rather than the bulky methoxy carbonyls [10], the absolute configuration of **7** and **8** both should be *S*.

### 2.5. Inhibitory Effects of **1**–**6** on Several Human Cancer Cell Lines

The inhibitory effect of **1**–**6** was tested by the 3-(4,5-dimethylthiazol-2-yl)-2,5-diphenyltetrazolium bromide (MTT) assay on human cancer K562, HL-60, HeLa, and BGC-823 cell lines. Compounds **1**–**6** inhibited the growth of the tested four human cancer cell lines at variable inhibition rates (IR%) at 100 μg/mL (Table 4). The half inhibitory concentration (IC_50_) of **1**–**3** on the K562 and HL-60 cell lines was determined as given below. **1**: 83.1 μg/mL (144.3 μM) for K562 and 75.3 μg/mL (130.7 μM) for HL-60; **2**: 69.0 μg/mL (119.8 μM) for K562 and 62.9 μg/mL (109.2 μM) for HL-60; **3**: 53.1 μg/mL (90.0 μM) for K562 and 54.7 μg/mL (92.7 μM) for HL-60.

**Table 4:** IR% values of **1***–***6** on human cancer cell lines at the 100 μg/mL. ^a^

Compound	K562	HL-60	HeLa	BGC-823
**1**	64.0%	71.6%	35.7%	36.8%
**2**	73.1%	77.4%	44.6%	41.4%
**3**	74.0%	80.0%	45.7%	46.8%
**4**	20.4%	26.0%	11.9%	—
**5**	36.1%	62.4%	13.9%	11.4%
**6**	38.8%	46.3%	29.1%	27.8%
**Decotaxol**	49.2%	46.9%	41.7%	44.1%

^a^ The cells were treated with the samples at 37 ºC for 24 h and then the IR% was measured by the MTT method; K562: Human chronic myelogenous leukemia K562 cell line, HL-60: Human acute promyelocytic leukemia HL-60 cell line, HeLa: Human cervical cancer HeLa cell line, BGC-823: Human gastric adenocarcinoma BGC-823 cell line; Decotaxol was used as positive control.

### 2.6. Discussion

In a continuation of our previous work on the C25 steroids newly produced in mutant AD-1-2 [30], further chromatography of the AD-1-2 extract, tracing newly produced chromones by bioassays and chemical analyses, resulted in the isolation of six choromone derivatives **1**–**6**, including the three new and rare chromones **2**–**4**. Two rare CPC monomers **7** and **8** were also found both to be produced in the mutant AD-1-2 by HPLC-PDAD-UV and HPLC-ESI-MS analyses. In contrast, none of **1***–***8** was detected in the parent G59 extract by the HPLC-PDAD-UV/HPLC-ESI-MS analyses. The proposed mechanism (Figure 4) according to the present results, extending the original proposal of Kong *et al.* [10], explored well the formation of **1***–***3** from **7** and **8**, including the conversion of the absolute configuration at C-3′ and the isomerism of **1***–***3** between the isomers at C-2′. Compounds **7** and **8** were detected in the ethyl acetate (EtOAc) extract of the mutant AD-1-2. We used MeOH and EtOH in the separation, but not in the extraction of AD-1-2 cultures to obtain the EtOAc extract. This thus excluded the doubt whether the methyl or ethyl ester in **7** and **8** were formed by esterification during the extraction. These results proved that the production of **4**–**8** in mutant AD-1-2 was caused by the activation of silent pathways in G59 strain by DES mutagenesis, although biological details for regulating the activation are still unknown, while **1**–**3** were formed by subsequently occurred spontaneous dimerization of the produced **7** and **8** *via* Diels-Alder reaction (Figure 4).

During NMR experiments in the present study, approximately 70% of **1** was converted into **2** after seven days at room temperature in DMSO-*d*_6_, evidenced by the appearance of a pair of the ^1^H NMR signals (**2a**/**2b** in 1:0.8 ratio), but no more than 70% of **1** was further converted into **2** even after 15 days. The conversion of **2** into **1** was not observed in DMSO-*d*_6_ solution. In the NMR experiments for crude **X**, which showed UV absorptions similar to **1**–**3** and had the molecular size (*m*/*z* 591 [M + H]^+^ in ESI-MS) the same as **3**, over 90% of **X** was converted into **3** within two days in DMSO-*d*_6_ at room temperature, and further purification of the samples provided an additional amount of **3** but **X** could not be obtained. Further, in the ^1^H NMR spectra of **3** in DMSO-*d*_6_ and CDCl_3_, very weak signals from **X** were detected together with some other also very weak signals from **III** (R=CH_2_CH_3_, in Figure 4), as seen in the ^1^H NMR spectra in the Supplementary Information. These weak signals always coexisted with the signals of **3** even after the samples for the NMR experiment were repeatedly purified by HPLC, indicating the presence of the conversion between **X** and **3** in the solutions. These observations further supported the mechanism in Figure 4, which involved the keto-enol tautomerism that enables both conversions of **1**/**2** and **3**/**X**. 

## 3. Experimental Section

### 3.1. General Experimental

Melting points were measured on a Beijing Tiandiyu X-4 exact micro melting point apparatus (Tiandiyu science and technology Co., Ltd., Beijing, China) and the temperatures were not corrected. Optical rotations were measured on an Optical Activity Limited polAAr 3005 spectropolarimeter (Optical Activity Limited, Ramsey, UK). ESIMS was recorded on an Applied Biosystems API 3000 LC-MS spectrometer (AB SCIEX, Framingham, MA, USA) and HRESIMS on an Agilent 6520 Q-TOF LC-MS spectrometer (Agilent Technologies, Santa Clara, CA, USA). UV data were taken on a GBC Cintra 20 spectrophotometer (GBC, Melbourne, Australia), IR spectra on a Bruker Tensor-27 infrared spectrophotometer (Bruker, Karlsruhe, Germany), CD on a Biologic Science MOS450 CD (Bio-Logic, Pont-de-Claix, France) and NMR spectra on a JEOL JNM-GX 400 (400 MHz ^1^H and 100 MHz ^13^C NMR) (JEOL Ltd., Tokyo, Japan) or Bruker-600 (600 MHz ^1^H and 150 MHz ^13^C NMR) NMR spectrometer (Bruker, Karlsruhe, Germany).

Precoated silica gel GF_254_ plates (10 cm × 20 cm, 0.25-mm thickness, Yantai Chemical Industrial Institute, Yantai, China) were used in TLC, and spots were detected under sunlight and UV light (254 and 365 nm) or by using Vaughan’s reagent [25,30,31] or 10% sulfuric acid reagent. Silica gel H (200–300 mesh, Yantai Chemical Industrial Institute, Yantai, China), YMC^*^GEL^®^ ODS-A-HG (12 nm S-50 μm, YMC Co., Ltd., Kyoto, Japan), and Sephadex™ LH-20 (GE Healthcare, Uppsala, Sweden) were used for column chromatography. HPLC were performed on Waters HPLC systems equipped with Waters 600 controller, Waters 600 pump, Waters 2414 refractive index detector, Waters 2996 (for analytical HPLC) or 2998 (for preparative HPLC) photodiode array detector, and Waters Empower™ software (Waters, Milford, MA, USA). Venusil MP C_18_ (5 μm, 100 Å, 4.6 mm × 250 mm; Agela Technologies, Tianjin, China), Capcell Pak C_18_ (UG120Å, 4.6 mm × 250 mm; Shiseido Co., Ltd., Tokyo, Japan), and Capcell Pak C_18_ (MG II, 4.6 mm × 250 mm; Shiseido Co., Ltd., Tokyo, Japan) columns were used in analytical HPLC, and Capcell Pak C_18_ (UG120Å, 20 mm × 250 mm; Shiseido Co., Ltd., Tokyo, Japan) and Capcell Pak C_18_ (MG II, 20 mm × 250 mm; Shiseido Co., Ltd., Tokyo, Japan) columns were used in preparative HPLC.

ZHWY-2102 rotary shakers (Shanghai ZhiCheng Analyzing Instrument Manufactory Co., Ltd., Shanghai, China) were used for fermentation. A VERSAmax-BN03152 micro plate reader (Molecular Devices, Silicon Valley, CA, USA) was used to read the optical density (OD) in the MTT assay and an AE31 EF-INV inverted microscope (Motic China Group Co., Ltd., Xiamen, Fujian, China) was used for examination of the tumor cell morphology.

Human chronic myelogenous leukemia K562 cell line was provided by Lili Wang (Beijing Institute of Pharmacology and Toxicology, Beijing, China), and Human acute promyelocytic leukemia HL-60, human cervical cancer HeLa and Human gastric adenocarcinoma BGC-823 cell lines by Wenxia Zhou (Beijing Institute of Pharmacology and Toxicology). Fetal bovine serum was purchased from Tianjin Hao Yang Biological manufacture Co., Ltd. (Tianjin, China), RPMI-1640 medium (lot No. 1403238) from Gibco (Grant Island, NY, USA), and MTT (lot No. 0793) from Amresco (Solon, OH, USA). Streptomycin (lot No. 071104) and penicillin (lot No. X11303302) were purchased from North China Pharmaceutical Group Corporation, Beijing, China, and docetaxol (DOC, lot No.20110326) from Aladdin Chemistry Co., Ltd. (Shanghai, China).

### 3.2. MTT Assay

EtOAc extracts and fractions were dissolved in MeOH at 10 mg/mL, and the MeOH solutions were used in MTT assays. Compounds **1**–**6** and DOC were dissolved in MeOH to prepare 10.0 mg/mL stock solutions, respectively, and serial dilutions for compounds **1**–**3** were made for MTT assay. DOC was used as positive control, and MeOH was used as blank control.

MTT assay was performed according to the procedure that we repeatedly used in the previous studies [22,23,24,25,26,27,28,29,30,31,32]. Exponentially growing K562, HL-60, HeLa, and BGC-823 cells were treated with samples at 37 °C for 24 h. The assay was run in triplicate, and the OD value was read at 570 nm on a VERSAmax-BN03152 plate reader. The IR% was calculated using OD mean values according to the formula, IR% = (OD_control_ − OD_sample_)/OD_control_ × 100%. The IC_50_ for compounds **1**–**3** was obtained from their IR% values at different concentrations.

### 3.3. Fermentation and Isolation of Compounds **1**–**6**

#### 3.3.1. Parent Fungal Strain and Its Mutant AD-1-2 the **1**–**8** Producing Strain

The parent strain *Penicillium purpurogenum* G59 was isolated from a soil sample collected at the tideland of Bohai Bay around Lüjühe in Tanggu district of Tianjin, China, in September 2004 [32], and was identified by Liang-Dong Guo, the Institute of Microbiology, Chinese Academy of Sciences, China. This strain was deposited at the China General Microbiological Culture Collection Center under the accession number CGMCC No. 9721.

AD-1-2 is a bioactive mutant obtained by DES mutagenesis of the G59 strain through treatment of G59 spores with 0.5% (*v*/*v*) DES in 50% (*v*/*v*) DMSO at 4 °C for 1 day [28]. The mutant AD-1-2 was deposited at the China General Microbiological Culture Collection Center under the accession number CGMCC No.8634.

#### 3.3.2. Fermentation, Extraction, and Preparation of Targeted Bioactive Fraction

By a large-scale fermentation (72 L) and extraction, we had previously obtained an EtOAc extract (37.3 g) of the mutant AD-1-2, showing inhibitory effect on K562 cells with an IR% of 58.6% at 100 μg/mL [30], and a vacuum liquid chromatography of the EtOAc extract (37.2 g) on a silica gel column (bed 7.5 cm × 20 cm, silica gel 300 g) eluted by b.p. 60–90 °C petroleum ether→dichloromethane (D)–methanol (M) 100:0→0:100 had afforded a fraction **Fr-3** (7.0 g, eluted by DM 98:2 → 96:4) [30]. **Fr-3** contained targeted chromone derivatives and inhibited K562 cells with an IR% of 53.1% at 100 μg*/*mL. Thus, **Fr-3** was further separated in the present study to isolate **1**–**6**, and the EtOAc extract of the mutant AD-1-2 was used in HPLC-PDAD-UV and HPLC-ESI-MS analyses to detect **1**–**8**. By fermentation and extraction of the parent G59 strain at the same time with the same conditions of the mutant AD-1-2, we had also previously obtained an EtOAc extract (610 mg) of the G59 strain, which did not inhibit the K562 cells (an IR% of 5.6% at 100 μg*/*mL) [30]. This extract was used for tracing newly produced **1**–**6** in the mutant AD-1-2 extract in the separation of **Fr-3** and also in the HPLC-PDAD-UV and HPLC-ESI-MS analyses for detecting **1**–**8**, all as negative controls, in the following experiment.

#### 3.3.3. Isolation of **1**–**6**

**Fr-3** (7.0 g) was subjected to Sephadex LH-20 column eluted by alcohol (95%) to give six fractions, **Fr-3-1** to **Fr-3-6** in the order of elution. **Fr-3-5** (2.0 g) contained targeted chromone derivatives and inhibited K562 cells with an IR% of 49.0% at 100 μg*/*mL. **Fr-3-5** (2.0 g) was thus further separated by a Sephadex LH-20 column eluted by dichloromethane–methanol (1:1) to give four fractions: **Fr-3-5-1** (100 mg), **Fr-3-5-2** (800 mg), **Fr-3-5-3** (300 mg), and **Fr-3-5-4** (800 mg). **Fr-3-5-2**, **Fr-3-5-3,** and **Fr-3-5-4** inhibited K562 cells with IR% of 43.0%, 78.0%, and 75.0% at 100 μg*/*mL, respectively.

**Fr-3-5-2** (800 mg) was subjected to preparative HPLC (Capcell Pak C_18_ column, UG120 Å, 5 μm, 20 mm × 250 mm; room temperature; mobile phase 55% methanol; flow rate 5.0 ml/min) to afford **4** (16 mg, *t*_R_ = 33.0 min) and **5** (24 mg, *t*_R_ = 25.6 min). The separation of **Fr-3-5-3** (300 mg) by the same preparative HPLC, except for the use of 70% methanol as mobile phase, gave **6** (18 mg, *t*_R_ = 45.6 min). **Fr-3-5-4** (800 mg) was separated by the same preparative HPLC, except for the use of 85% methanol as mobile phase, to afford crude **1** (*t*_R_ 27.11 min), **2** (*t*_R_ 26.16 min), **3** (*t*_R_ 28.91 min), and **X** (*t*_R_ 30.65 min). The whole crude **1**–**3** samples were subjected again, without weighing, to preparative HPLC (Capcell Pak C_18_ column, MG II, 5 μm, 20 mm × 250 mm; room temperature; mobile phase 70% acetonitrile; flow rate 8.0 mL/min) to obtain **1** (5.3 mg, *t*_R_ = 40.6 min), **2** (12.5 mg, *t*_R_ = 38.4 min), and **3** (6.2 mg, *t*_R_ = 43.5 min), respectively.

### 3.4. Physicochemical and Spectroscopic Data of **1**–**6**

Remisporine B (**1**): a yellow amorphous powder (MeOH), ${\left[\alpha \right]}_{D}^{25}$ +898.9 (*c* 0.18, MeOH). Positive ESIMS *m*/*z*: 559 [M − H_2_O + H]^+^, 577 [M + H]^+^, 599 [M + Na]^+^; negative ESI-MS *m*/*z*: 575 [M − H]^−^. UV λ_max_ nm (log *ε*) in MeOH: 204.6 (4.34), 227.2 (4.34), 240.5 (4.40), 259.0 (shoulder peak, 4.25), and 323.3 (3.71). CD (MeOH) Δε (nm): +13.41 (207.0), 0 (214.5), −1.02 (219), −5.19 (230.5), 0 (239.0), +15.46 (259.5), 0 (271.0), −2.91 (280.0), 0 (292.5), +5.29 (332.0), 0 (438.5). ^1^H and ^13^C NMR data in DMSO-*d*_6_: Table 1; ^1^H NMR data in CDCl_3_: Table 2.

Epiremisporine B (**2**): a yellow amorphous powder (MeOH), ${\left[\alpha \right]}_{D}^{25}$ +524.4 (*c* 0.16, MeOH). Positive ESIMS *m*/*z*: 559 [M − H_2_O + H]^+^, 577 [M + H]^+^, 599 [M + Na]^+^; negative ESI-MS *m*/*z*: 575 [M − H] ^−^. Positive HRESIMS *m*/*z*: measured 577.1349 [M + H]^+^, calculated for C_30_H_2__5_O_12_ [M + H]^+^ 577.1346. UV λ_max_ nm (log *ε*) in MeOH: 206.3 (4.42), 227.6 (4.45), 240.4 (4.50), 258.0 (shoulder peak, 4.40), and 323.2 (3.83). IR ν_max_ cm^−1^ (Diamond ATR crystal): 3600–2500 (br, OH), 2955 (CH_3_/CH_2_/CH), 1742 (ester carbonyl), 1656 (conjugated carbonyl), 1626, 1598, 1491 (Ar-ring), 1447, 1364, 1282, 1205, 1063, 1027, 1005, 899, 875, 826. CD (MeOH) Δε (nm): +15.31 (207.0), +2.29 (220.5), 0 (226.5), −2.50 (238.0), 0 (241.5), +14.79 (260.0), 0 (269.5), −7.48 (281.5), 0 (296.5), +4.99 (332.0), 0 (438.5). ^1^H and ^13^C NMR data in DMSO-*d*_6_: Table 1; ^1^H NMR data in CDCl_3_: Table 3.

Epiremisporine B1 (**3**): a yellow amorphous powder (MeOH), ${\left[\alpha \right]}_{D}^{25}$ +531.6 (*c* 0.07, MeOH). Positive ESIMS *m*/*z*: 573 [M − H_2_O + H]^+^, 591 [M + H]^+^, 613 [M + Na]^+^ , 629 [M + K]^+^; negative ESI-MS *m*/*z*: 589 [M − H]^−^. Positive HRESIMS *m*/*z*: measured 591.1497 [M + H]^+^, calculated for C_30_H_2__5_O_12_ [M + H]^+^ 591.1503. UV λ_max_ nm (log *ε*) in MeOH: 205.4 (4.33), 227.5 (4.36), 241.3 (4.43), 259.5 (shoulder peak, 4.33), and 325.8 (3.74). IR ν_max_ cm^−1^ (Diamond ATR crystal): 3600–2500 (br, OH), 2957, 2919, 2850, 2830 (CH_3_/CH_2_/CH), 1748 (ester carbonyl), 1653 (conjugated carbonyl), 1623, 1600, 1490 (Ar-ring), 1446, 1420, 1356, 1290, 1200, 1068, 1034, 1019, 934, 919, 876, 824. CD (MeOH) Δε (nm): +18.49 (207.0), +3.28 (221.0), 0 (229.0), −2.56 (235.5), 0 (241.0), +16.03 (258.5), 0 (271.0), −4.38 (281.5), 0 (296.0), +4.77 (331.5), 0 (431). ^1^H and ^13^C NMR data in DMSO-*d*_6_: Table 1; ^1^H NMR data in CDCl_3_: Table 3.

Isoconiochaetone C (**4**): colorless needles (MeOH), m.p. 99–100 °C, ${\left[\alpha \right]}_{D}^{25}$ +76.7 (*c* 0.16, MeOH). Positive ESIMS *m*/*z*: 215 [M − CH_3_OH + H]^+^, 247 [M + H]^+^, 269 [M + Na]^+^, 515 [2M + Na]^+^. Positive HRESIMS *m*/*z*: measured 247.0962 [M + H]^+^, calculated for C_30_H_2__5_O_12_ [M + H]^+^ 247.0970. UV λ_max_ nm (log *ε*) in MeOH: 203.7 (4.22), 228.0 (4.30), 238.7 (4.35), 257.0 (shoulder peak, 4.19), and 323.2 (3.67). ^1^H and ^13^C NMR data in CDCl_3_: Table 2.

Coniochaetone A (**5**): colorless needles (MeOH), m.p. 179−180 °C, Positive ESI-MS *m*/*z*: 231 [M + H]^+^, 253 [M + Na]^+^, 269 [M + K]^+^, 483 [2M + Na]^+^; negative ESI-MS *m*/*z*: 229 [M − H]^−^. ^1^H NMR (CDCl_3_, 400 MHz) *δ*: 12.23 (1H, s, HO-10), 6.78 (1H, br s, H-7), 6.69 (1H, br s, H-9), 3.11–3.08 (2H, m, H_2_-3), 2.73–2.70 (2H, m, H_2_-2), 2.42 (3H, s, H_3_-14). ^13^C NMR (100 MHz, CDCl_3_) *δ*: 197.6 (C-1), 189.8 (C-4), 178.2 (C-12), 162.0 (C-10), 156.5 (C-6), 148.4 (C-8), 118.1 (C-13), 114.4 (C-9), 108.8 (C-11), 108.2 (C-7), 33.9 (C-2), 26.3 (C-3), 22.5 (C-14). The MS and NMR data are identical with those in the literature [1].

Methyl 8-hydroxy-6-methyl-9-oxo-9*H*-xanthene-1-carboxylate (**6**): Pale yellow needles (MeOH), m.p. 194-195 °C, Positive ESI-MS *m*/*z*: 285 [M + H]^+^, 307 [M + Na]^+^, 591 [2M + Na]^+^. 1H NMR (CDCl_3_, 400MHz) *δ*: 12.13 (1H, s, HO-8), 7.73 (1H, dd, *J* = 8.5, 7.3 Hz, H-3), 7.50 (1H, dd, *J* = 8.5, 0.9 Hz, H-4), 7.29 (1H, dd, *J* = 7.3, 0.9 Hz, H-2), 6.72 (1H, br s, H-5), 6.61 (1H, br s, H-7), 4.02 (3H, s, OCH_3_), 2.41 (3H, s, 6-CH_3_). ^13^C NMR (100 MHz, CDCl_3_) *δ*: 180.5 (C-9), 169.8 (CO_2_CH_3_), 161.5 (C-8), 156.0 (C-4a), 155.7 (C-4b), 149.5 (C-6), 134.9 (C-3), 133.6 (C-1), 122.6 (C-2), 119.5 (C-4), 117.6 (C-9a), 111.8 (C-7), 107.5 (C-5), 107.0 (C-8a), 53.2 (OCH_3_), 22.7 (6-CH_3_). The MS and NMR data are identical with those in the literature [33].

### 3.5. HPLC-PDAD-UV Analysis of the AD-1-2 and G59 Extracts for Detecting **1**–**8**

The EtOAc extracts of mutant AD-1-2 and G59 strain were dissolved in MeOH to prepare sample solutions at 10 mg/mL for HPLC analysis. Crude samples of **1**–**6** in MeOH at 10 mg/mL were used as reference standards in the HPLC-PDAD-UV analysis. The **4** and **5** solutions in MeOH at 10 mg/mL were mixed in a ratio of 4:6 and the mixed solution was used for shortening HPLC running times.

The HPLC-PDAD-UV analysis was carried out on a Venusil MP C_18_ column (5 μm, 100 Å, 4.6 mm × 250 mm; Agela Technologies, Tianjin, China) using the Waters HPLC equipment given in Section 3.1. Sample and standard solutions were filtered using 0.22 μm pore membrane filters, and each 5 μl of the solutions was injected into the column. Elution was performed using MeOH–H_2_O in linear gradient (20% MeOH at initial time 0 min→100% MeOH at 60 min→100% MeOH at 90 min; flow rate, 1.0 mL/min). The acquired photodiode array data were processed by the Waters Empower™ software to obtain targeted HPLC-PDAD-UV data. Compounds **1**–**6** were eluted as peaks with *t*_R_ of 58.90 min for **1**, 58.30 min for **2**, 63.70 min for **3**, 39.38 min for **4**, 33.23 min for **5**, and 52.58 min for **6**, and their detection was achieved both by *t*_R_ and UV spectra. The peaks of **7** (*t*_R_ = 38.07 min) and **8** (*t*_R_ = 37.38 min) were detected by their typical UV absorptions around 270 and 340 nm [10].

### 3.6. HPLC-ESI-MS Analysis of the AD-1-2 and G59 Extracts for Detecting **1**–**8**

The MeOH solution of the EtOAc extracts of mutant AD-1-2 and the control G59 strain, which had been used in the HPLC-PDAD-UV analysis, was used in HPLC-ESI-MS analysis. The HPLC-ESI-MS analysis was performed on an LC-MS equipment equipped with Agilent 1100 HPLC system, AB Sciex API 3000 LC-MS/MS system and AB Sciex Analyst 1.4 software (AB SCIEX, Framingham, MA, USA). HPLC was carried out on the same Venusil MP C_18_ column (5 μm, 100 Å, 4.6 mm × 250 mm; Agela Technologies, Tianjin, China) at the same conditions of HPLC-PDAD-UV analysis. The mass detector was set to scan a range from *m*/*z* 150–1500 both in the positive and negative modes. The acquired data were processed by the Analyst 1.4 software to obtain targeted HPLC-ESI-MS data. The *pseudo*-molecular ions of **1**–**8** appeared as peaks with *t*_R_ of 55.0–57.0 min for **1**/**2**, 60.40–61.41 min for **3**, 36.60–37.00 min for **4**, 30.60–31.00 min for **5**, 49.69–49.89 min for **6**, 35.19–35.59 min for **7**, and 34.89–35.12 min for **8**. The retention times were slightly shorter than in HPLC-PDAD-UV analysis by the shortened flow length from the outlet of the HPLC column to the inlet of MS in the HPLC-ESI-MS. Detection of **1**–**8** was achieved by selective ion (*m*/*z*: 575 [M − H]^−^ for **1**/**2**, 589 [M − H]^−^ for **3**, 215 [M − CH_3_OH + H]^+^ for **4**, 231 [M + H]^+^ for **5**, 307 [M + Na]^+^ for **6**, 311 [M + Na]^+^ for **7**, 341 [M + K]^+^ for **8**) monitoring with both extracted ion chromatograms and related MS spectra.

### 3.7. Computation Section for ECD Calculation

Conformational searches were performed employing the “systematic” procedure implemented in Spartan′14 [34], using MMFF (Merck molecular force field). All MMFF minima were re-optimized with density functional theory (DFT) calculations at the B3LYP/6-31+G(d) level for **1** and **2** and at the B3LYP/6-31G(d) level for **3** using the Gaussian 09 program [35]. The geometry was optimized starting from various initial conformations, with vibrational frequency calculations confirming the presence of minima. Theoretical ECD calculations on the optimized conformers were run by TDDFT calculations performed on five lowest-energy conformations (>5% population) for each of the **1a** and *ent*-**1a** configurations (Figure S3) using 20 excited states for **1**, three (for **2a** and **2b**) or four (for *ent*-**2a** ) or one (for *ent*-**2b**) lowest-energy conformations (>5% population) for **2a**, *ent*-**2a**, **2b** and *ent*-**2b** configurations (Figure S4) using 30 excited states for **2**, and seven (for **3a** and *ent*-**3a**) or five (for **3b**) or six (for *ent*-**3b**) lowest-energy conformations (>5% population) for **3a**, *ent*-**3a**, **3b** and *ent*-**3b** configurations (Figure S5) using 30 excited states for **3**, respectively. A polarizable continuum model (PCM) in MeOH was adopted to consider solvent effects in the TDDFT calculations. CD spectra were generated using the program SpecDis [36] by applying a Gaussian band shape with 0.26 eV width, from dipole-length rotational strengths. The dipole velocity forms yielded negligible differences. The ECD spectra of the conformers were combined using Boltzmann weighting, with the lowest-energy conformations accounting for 98% of the weights for **1**, 99% of the weights for **2**, and 97% (for **3a** and *ent*-**3a**) or 99% (for **3b** and *ent*-**3b**) of the weights for **3**, respectively. The calculated spectrum was blue-shifted by 5 nm for **1** and red-shifted by 6 nm for **2** and **3** to facilitate comparison with the experimental data.

## 4. Conclusions

A chemical investigation on a mutant AD-1-2 of marine-derived fungus *P. purpurogenum* G59 resulted in the discovery of three new chromones **2**–**4** and the isolation of three known chromones **1**, **5**, and **6**. Two rare CPCs **7** and **8** were also found to be produced in the mutant AD-1-2, but none of **1***–***8** were detected in the G59 extract. The present work further exemplified the effectiveness of our previous DES mutagenesis strategy [28,29,30,31] for activating silent fungal pathways to discover new bioactive compounds.

## Supplementary Files

Supplementary File 1
