# Supplementary material for: Rare Chromones from a Fungal Mutant of the Marine-Derived Penicillium purpurogenum G59
Source: Mar Drugs. 2015 Aug 18;13(8):5219–36. doi: 10.3390/md13085219 (PMC4557021; doi:10.3390/md13085219)
Supplement: Supplementary File 1 [file marinedrugs-13-05219-s001.docx]

Supplementary Information

**Table S1.** 600 MHz ^1^H and 150 MHz ^13^C NMR data of **2** in DMSO-*d*_6_. ^a^

| **Position** | **2a (2′β-OH: 2′*S*)** | | | | **2b (2′α-OH: 2′*R*)** | | | |
| --- | --- | --- | --- | --- | --- | --- | --- | --- |
|  | **δ_H_ (*J* in Hz)** | **δ_C_** | **HMBC ^b^** | **NOE ^c^** | **δ_H_ (*J* in Hz)** | **δ_C_** | **HMBC ^b^** | **NOE ^c^** |
| 1 | — | 170.8 s | — | — | — | 171.3 s | — | — |
| 2 | — | 88.2 s | — | — | — | 89.5 s | — | — |
| 3 | 3.78 (dd, 9.2, 9.0) | 47.0 d | C-1, 2, 3′, 4, 4′, 5, 14 | H-3′, H-4 | 3.88 (dd, 9.0, 8.4) | 46.6 d | C-1, 2, 3′, 4, 4′, 5, 14 | H-3′, H-4, 2′–OH |
| 4 | 4.98 (d, 9.0) | 36.9 d | C-3, 3′, 5, 5′, 13′, 14, 14′,15 | H-3 | 5.02 (d, 9.0) | 36.2 d | C-3, 3′, 5, 5′, 13′, 14, 14′,15 | H-3 |
| 5 | — | 169.3 s | — | — | — | 168.9 s | — | — |
| 7 | — | 156.78 s | — | — | — | 156.75 s | — | — |
| 8 | 6.79 (s) | 108.3 d | C-7, 10, 12, 13,15 | H-15 | 6.77 (s) | 108.3 d | C-7, 10, 12, 13,15 | H-15 |
| 9 | — | 147.5 s | — | — | — | 147.5 s | — | — |
| 10 | 6.64 (s) | 112.3 d | C-7, 8, 12, 13,15 | H-15 | 6.64 (s) | 112.3 d | C-7, 8, 12, 13,15 | H-15 |
| 11 | — | 159.87 s | — | — | — | 159.85 s | — | — |
| 12 | — | 108.4 s | — | — | — | 108.4 s | — | — |
| 13 | — | 178.87 s | — | — | — | 178.89 s | — | — |
| 14 | — | 119.2 s | — | — | — | 118.7 s | — | — |
| 15 | 2.30 (s) | 21.5 q | C-8, 9, 10 | H-8, H-10 | 2.28 (s) | 21.4 q | C-8, 9, 10 | H-8, H-10 |
| 16 | 3.69 (s) | 52.8 q | C-1 | — | 3.70 (s) | 52.6 q | C-1 | — |
| 1′ | — | 169.4 s | — | — | — | 167.7 s | — | — |
| 2′ | — | 105.7 s | — | — | — | 106.2 s | — | — |
| 3′ | 3.10 (ddd, 10.1, 9.2, 6.4) | 42.7 d | C-1′, 2, 2′, 3, 4′, 5′ | H-3, Hα-4′, Hβ-4′, 2′–OH | 2.79 (ddd, 12.5, 8.4, 5.9) | 47.3 d | C-1′, 2, 2′, 3, 4′, 5′ | H-3, Hα-4′, Hβ-4′, 2′–OH |
| 4′α | 2.70 (dd, 17.0, 6.4) | 26.3 t | C-2′, 3, 3′, 5′, 14′ | H-3′, Hβ-4′, 2′–OH | 2.48 (dd, 15.9, 5.9) | 26.9 t | C-2′, 3, 3′, 5′, 14′ | H-3′, Hβ-4′, H_3_-16 |
| β | 2.64 (dd, 17.0, 10.1) |  | C-2′, 3, 3′, 5′, 14′ | H-3′, Hα-4′, 2′–OH | 2.43 (dd, 15.9, 12.4) |  | C-2′, 3, 3′, 5′, 14′ | H-3′, Hα-4 |
| 5′ | — | 168.0 s | — | — | — | 167.4 s | — | — |
| 7′ | — | 155.5 s | — | — | — | 155.6 s | — | — |
| 8′ | 6.90 (s) | 107.6 d | C-7′, 10′, 12′, 13′,15′ | H-15′ | 6.88 (s) | 107.6 d | C-7′, 10′, 12′, 13′,15′ | H-15′ |
| 9′ | — | 147.40 s | — | — | — | 147.44 s | — | — |
| 10′ | 6.71 (s) | 112.0 d | C-7′, 8′, 12′, 13′,15′ | H-15′ | 6.71 (s) | 112.0 d | C-7′, 8′, 12′, 13′,15′ | H-15′ |
| 11′ | — | 159.52 s | — | — | — | 159.55 s | — | — |
| 12′ | — | 107.7 s | — | — | — | 107.8 s | — | — |

**Table S1.** *Cont*.

| 13′ | — | 179.4 s | — | — | — | 179.3 s | — | — |
| --- | --- | --- | --- | --- | --- | --- | --- | --- |
| 14′ | — | 111.9 s | — | — | — | 111.7 s | — | — |
| 15′ | 2.38 (s) | 21.77 q | C-8′, 9′, 10′ | H-8′, H-10′ | 2.38 (s) | 21.79 q | C-8′, 9′, 10′ | H-8′, H-10′ |
| 16′ | 3.74 (s) | 52.28 q | C-1′ | — | 3.75 (s) | 52.31 q | C-1′ | Hα-4′, Hβ-4′ |
| 11–OH | 12.15 (s) | — | C-9, 10, 11, 12 | H-10 | 12.12 (s) | — | C-9, 10, 11, 12 | H-10 |
| 2′–OH | 7.80 (s) | — | C-1′, 2′, 3′ | Hα-4′, Hβ-4′ | 7.53 (s) | — | C-1′, 2′, 3′ | H-3, H-3′ |
| 11′–OH | 12.49 (s) | — | C-9′, 10′, 11′, 12′ | H-10′ | 12.47 (s) | — | C-9′, 10′, 11′, 12′ | H-10′ |

^a^ Chemical shifts of ^1^H and ^13^C nuclei were recorded in δ values using the solvent DMSO-*d*_6_ signals (δ_H_ 2.50/δ_C_ 39.52) as references, respectively, and the signals were assigned on the basis of HMQC, HMBC, NOESY, and 1D GOESY experiments. The ratio of **2a** to **2b** was determined approximately to be 1:0.8 by the standard integrals of the H-4, HO-2′ and H-3′ signals in the DMSO-*d*_6_ solution of **2**. ^b^ Numbers in each line of this column indicate the carbons that showed HMBC correlations with the proton in the corresponding line in the HMBC experiments optimized for the 8.3 Hz of long-range *J*_CH_ value. ^c^ Numbers in each line of this column indicate the protons that showed NOE correlations with the proton in the corresponding line in 2D NOESY or 1D GOESY experiments. The NOEs between two protons in a spin coupling relationship were detected by the 1D GOESY experiments. In the 1D GOESY experiments, a more remarkably NOE was detected on 2′-OH in **2b** than in **2a** by irradiating H-3′ in **2a** and **2b**, respectively.

| 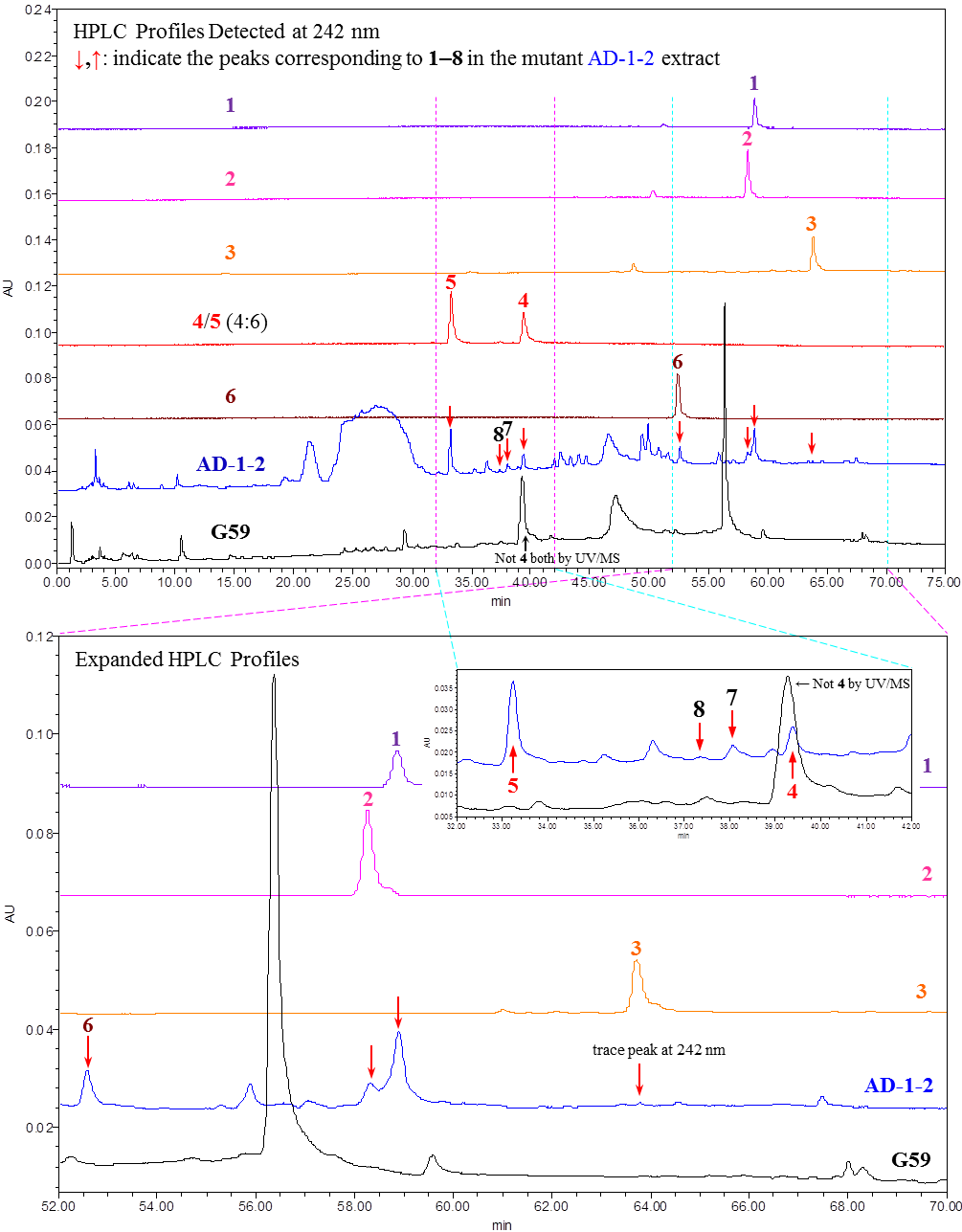 |
| --- |
| (**A**) |

**Figure S1.** Cont.

| 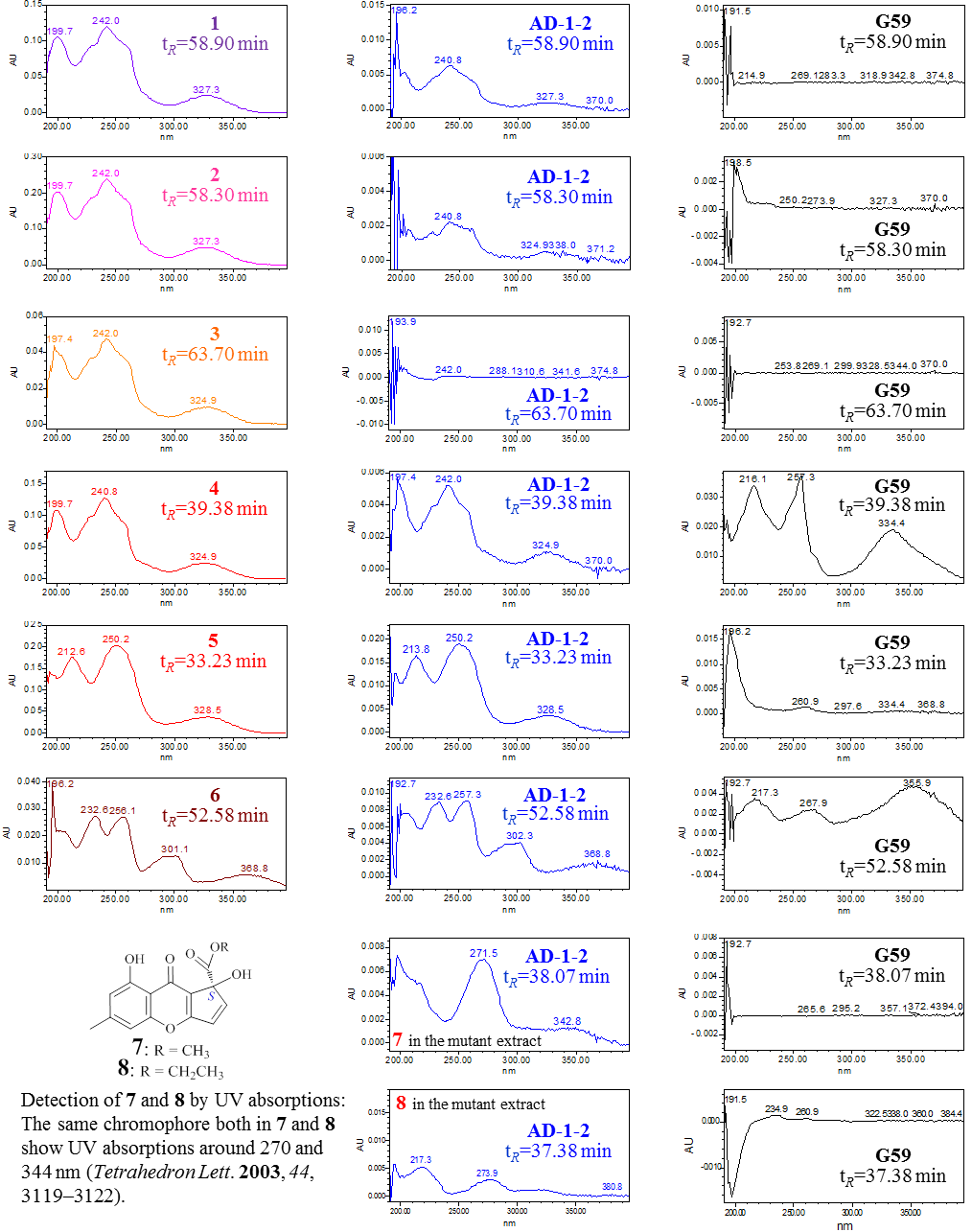 |
| --- |
| (**B**) |

**Figure S1.** *Cont.*

| 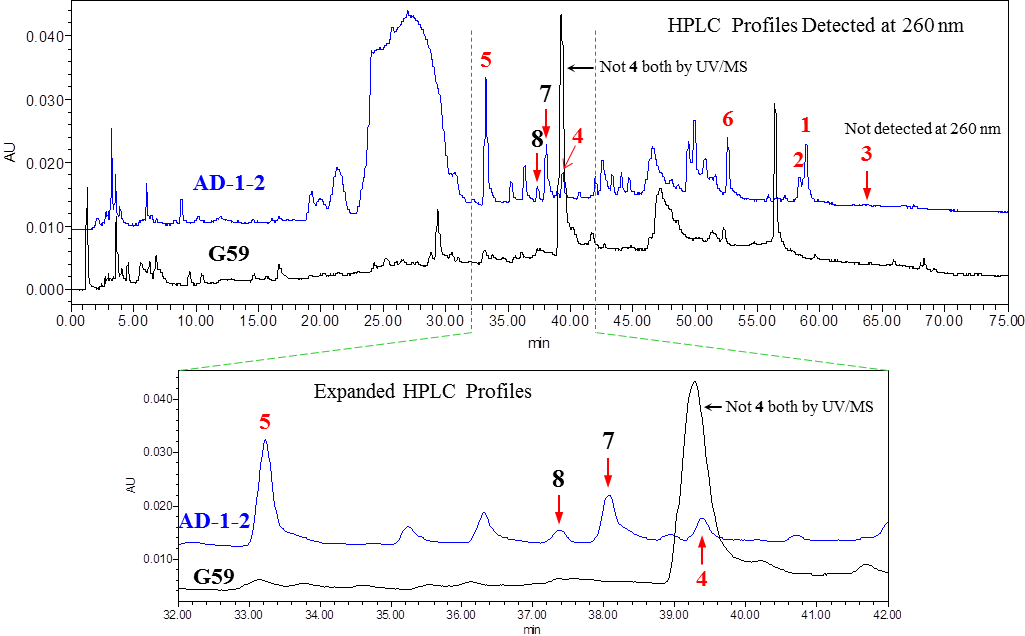 |
| --- |
| (**C**) |
| 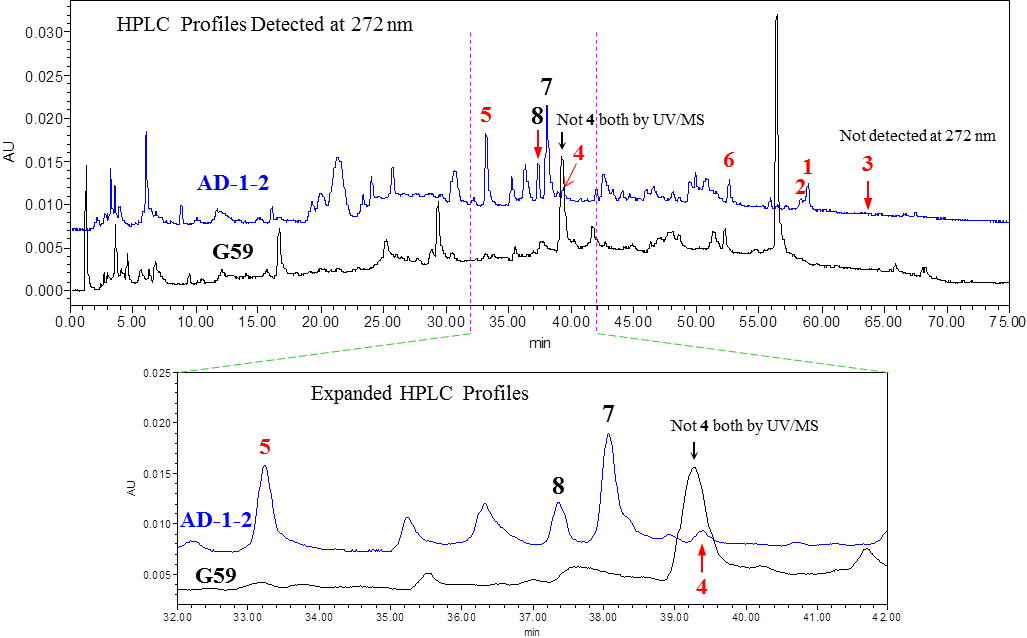 |
| (**D**) |

**Figure S1.** HPLC-PDAD-UV analysis of the EtOAc extracts of mutant AD-1-2 and the parent G59 strain for detecting **1**–**8**. (**A**) HPLC profiles of **1**–**6** and the AD-1-2 and G59 extracts detected at 242 nm; (**B**) Detection of **1**–**8** in the AD-1-2 and G59 extracts by the UV absorptions in the HPLC profiles detected at 242 nm; (**C**) HPLC profiles of the EtOAc extracts of AD-1-2 and G59 cultures detected at 260 nm; (**D**) HPLC profiles of the EtOAc extracts of AD-1-2 and G59 cultures detected at 272 nm.

| 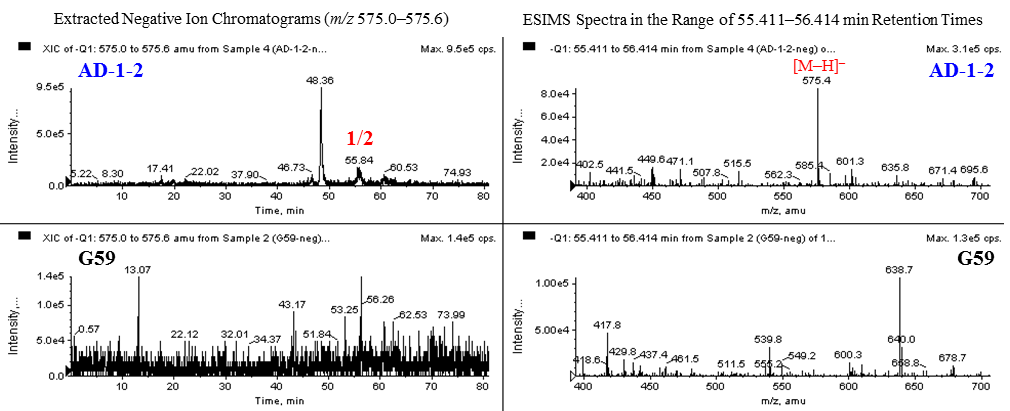 |
| --- |
| (**A**) |
| 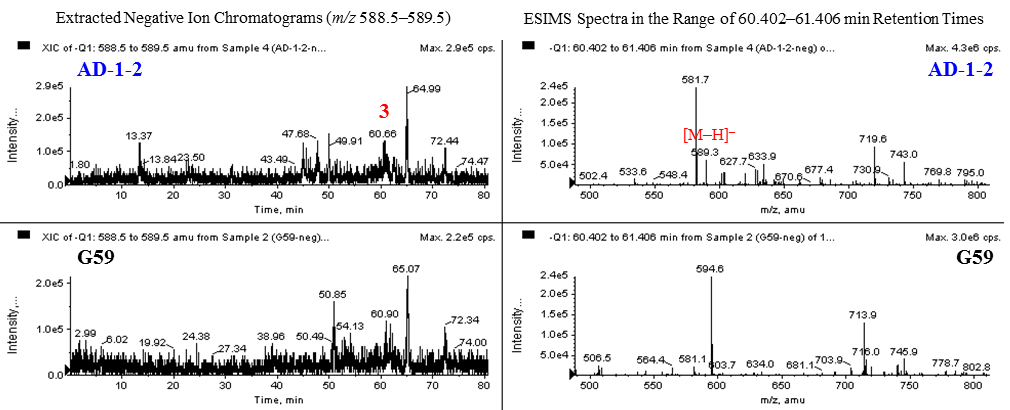 |
| (**B**) |
| 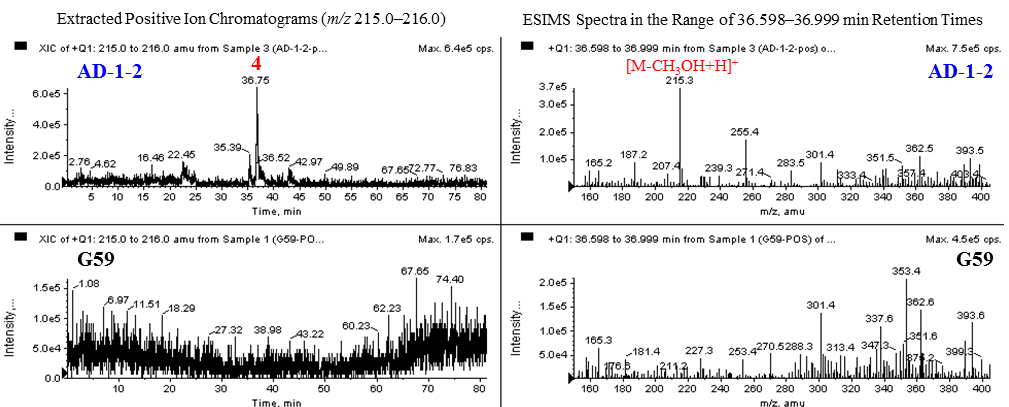 |
| (**C**) |

**Figure S2.** *Cont.*

| 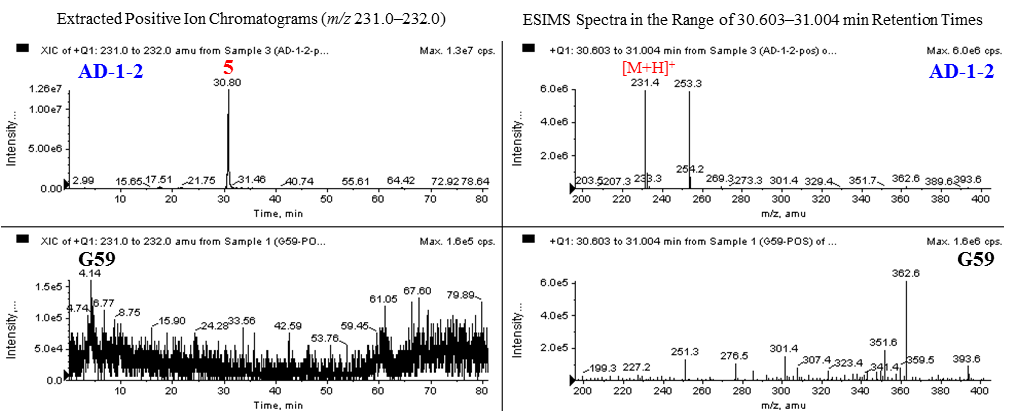 |
| --- |
| (**D**) |
| 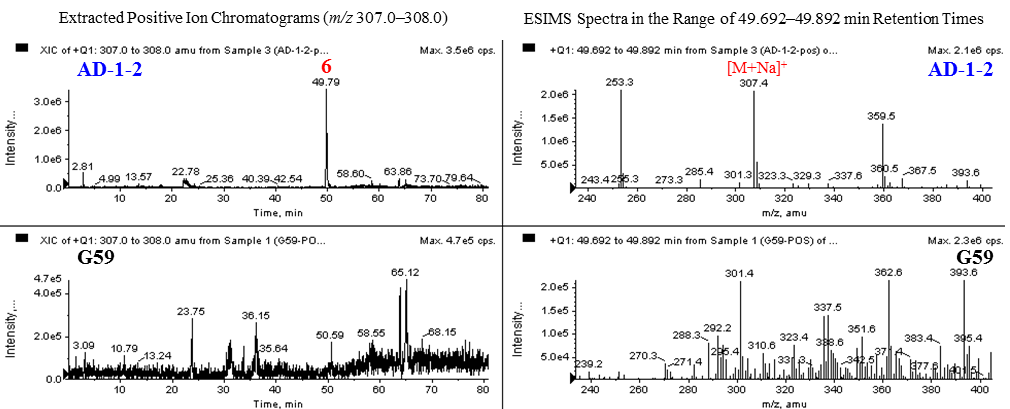 |
| (**E**) |
| 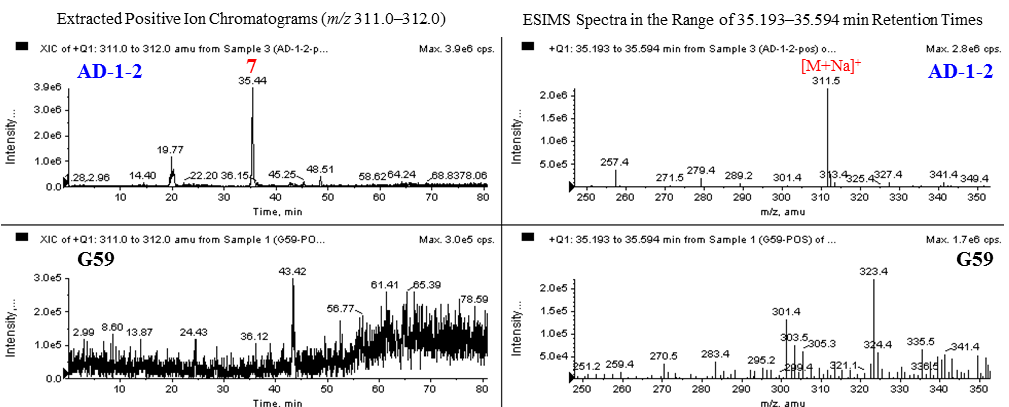 |
| (**F**) |

**Figure S2.** *Cont.*

| 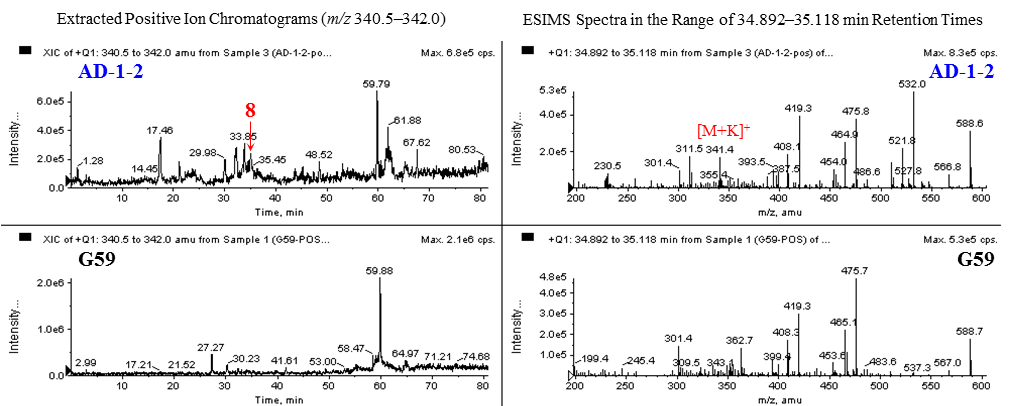 |
| --- |
| (**G**) |

**Figure S2.** HPLC-ESI-MS analysis of the EtOAc extracts of mutant AD-1-2 and the
parent G59 strain for detecting **1**–**8**. (**A**) HPLC-Negative ion ESI-MS analysis (ESIMS
*m*/*z*: 575 [M − H]^−^ for both **1** and **2**); (**B**) HPLC-Negative ion ESI-MS analysis (ESIMS *m*/*z*: 589 [M − H]^−^ for **3**); (**C**) HPLC-Positive ion ESI-MS analysis (ESIMS
*m*/*z*: 215 [M-CH_3_OH + H]^+^ for **4**); (**D**) HPLC-Positive ion ESI-MS analysis (ESIMS
*m*/*z*: 231 [M + H]^+^ for **5**); (**E**) HPLC-Positive ion ESI-MS analysis (ESIMS
*m*/*z*: 307 [M + Na]^+^ for **6**); (**F**) HPLC-Positive ion ESI-MS analysis (ESIMS
*m*/*z*: 311 [M + Na]^+^ for **7**); (**G**) HPLC-Positive ion ESI-MS analysis (ESIMS
*m*/*z*: 341 [M + K]^+^ for **8**).

| 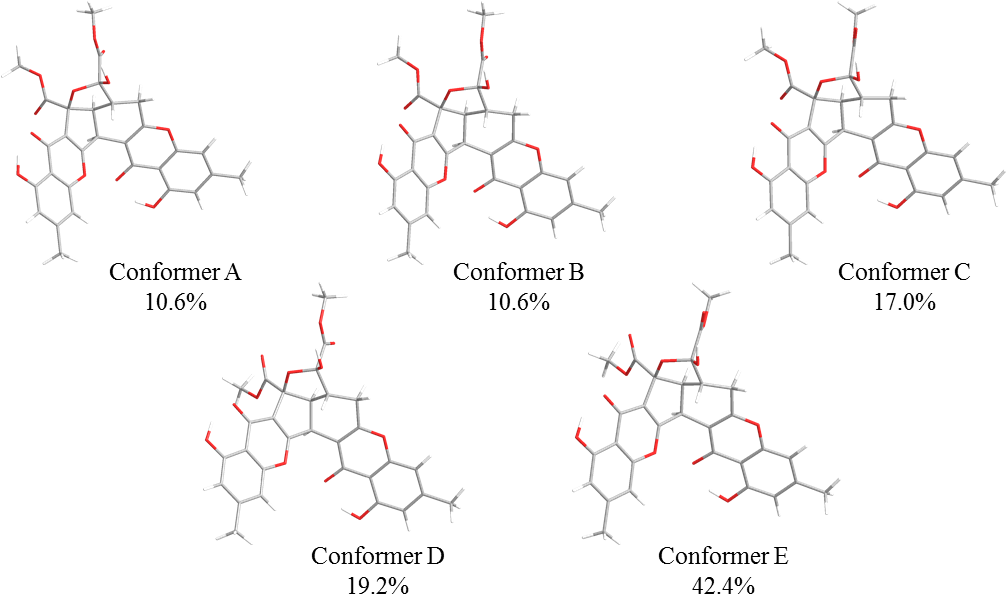 |
| --- |
| (**A**) |

**Figure S3.** *Cont.*

| 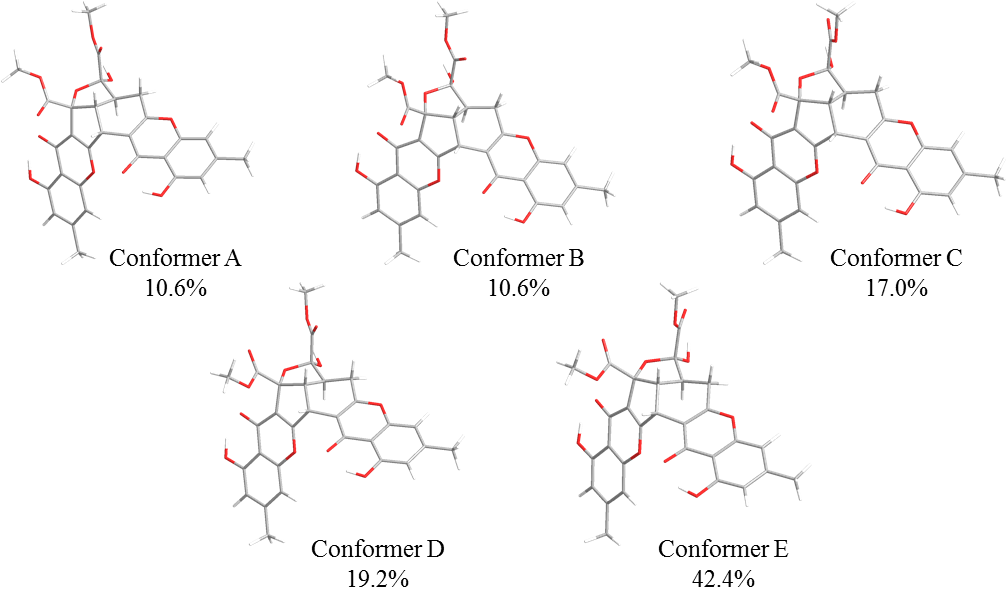 |
| --- |
| (**B**) |

**Figure S3.** DFT-optimized structures for low-energy conformers of **1a**/*ent*-**1a** at the B3LYP/6-31+g(d) level using a polarizable continuum model (PCM) in MeOH. Conformer populations were calculated using the Gibbs free energy and Boltzmann population at 298 K estimated thereof; (**A**) Low-energy conformers of **1a** (2*S*,3*R*,4*S*,2′*S*,3′*R*); (**B**) Low-energy conformers of *ent*-**1a** (2*R*,3*S*,4*R*,2′*R*,3′*S*).

| **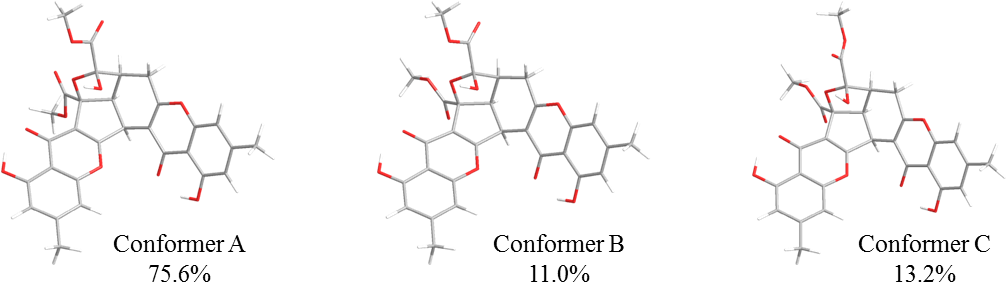** |
| --- |
| (**A**) |

**Figure S4.** *Cont.*

| **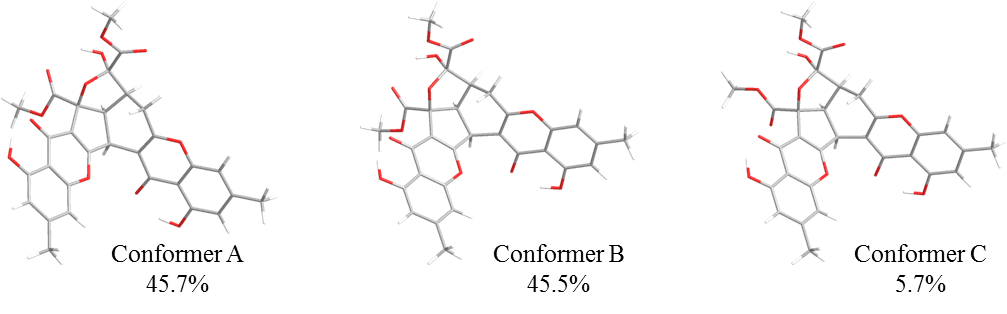** |
| --- |
| (**B**) |
| **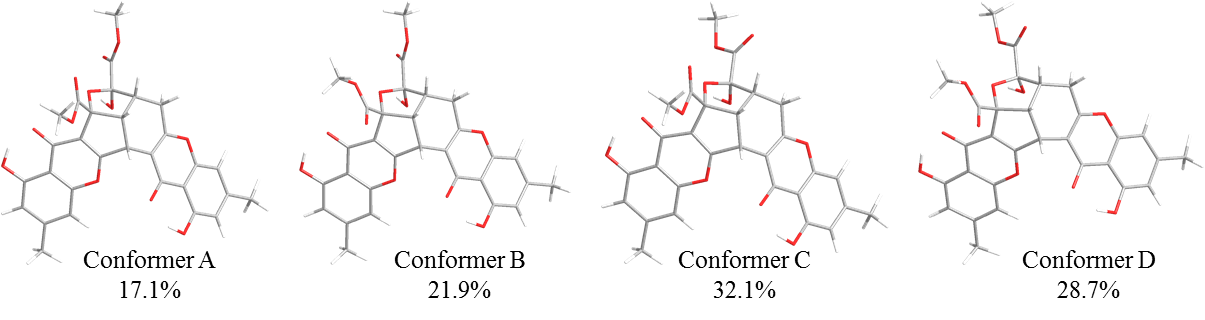** |
| (**C**) |
| **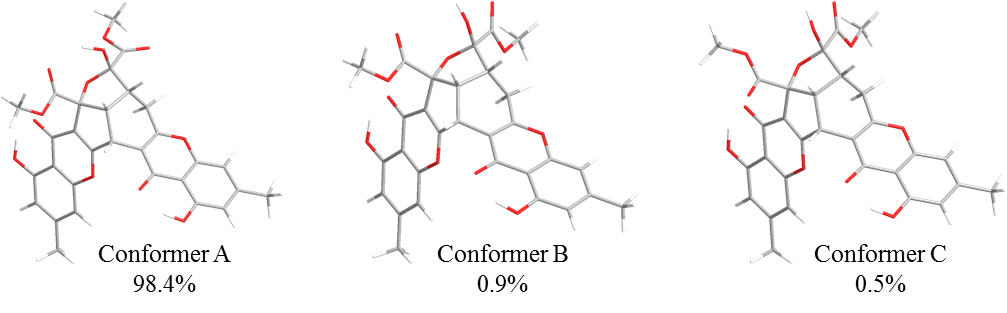** |
| (**D**) |

**Figure S4.** DFT-optimized structures for low-energy conformers of **2a**, **2b**, *ent*-**2a** and *ent*-**2b** for **2** in vacuum at the B3LYP/6-31+g(d) level. Conformer populations were calculated using the Gibbs free energy and Boltzmann population at 298 K estimated thereof; (**A**) Low-energy conformers of **2a** (2*S*3*R*4*S*2′*S*3′*S*); (**B**) Low-energy conformers of **2b** (2*S*3*R*4*S*2′*R*3′*S*); (**C**) Low-energy conformers of *ent*-**2a** (2*R*3*S*4*R*2′*R*3′*R*); (**D**) Low-energy conformers of *ent*-**2b** (2*R*3*S*4*R*2′*S*3′*R*).

| 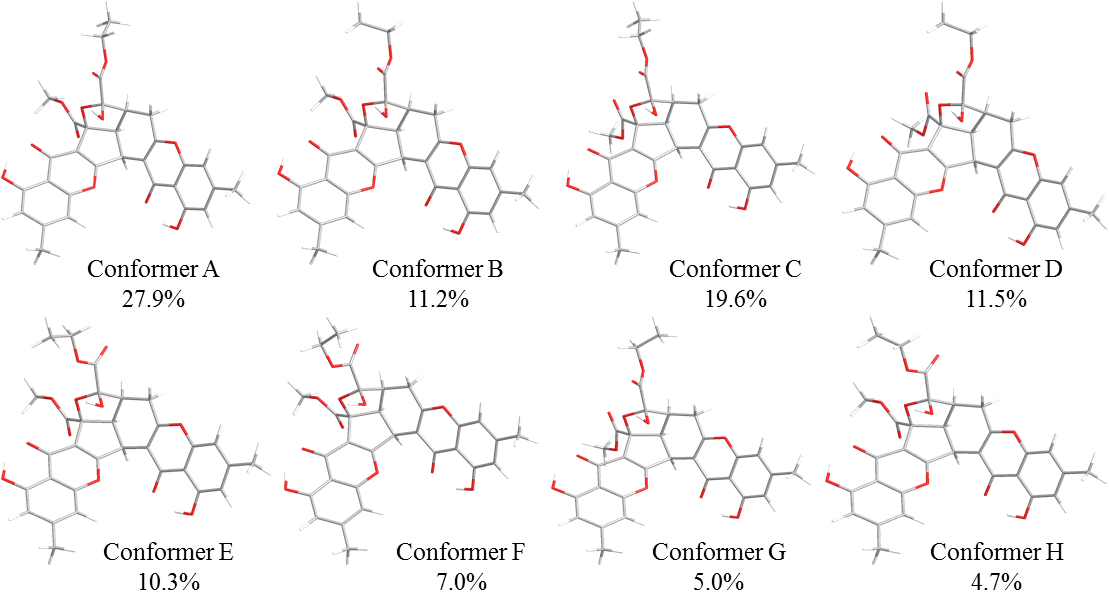 |
| --- |
| (**A**) |
| 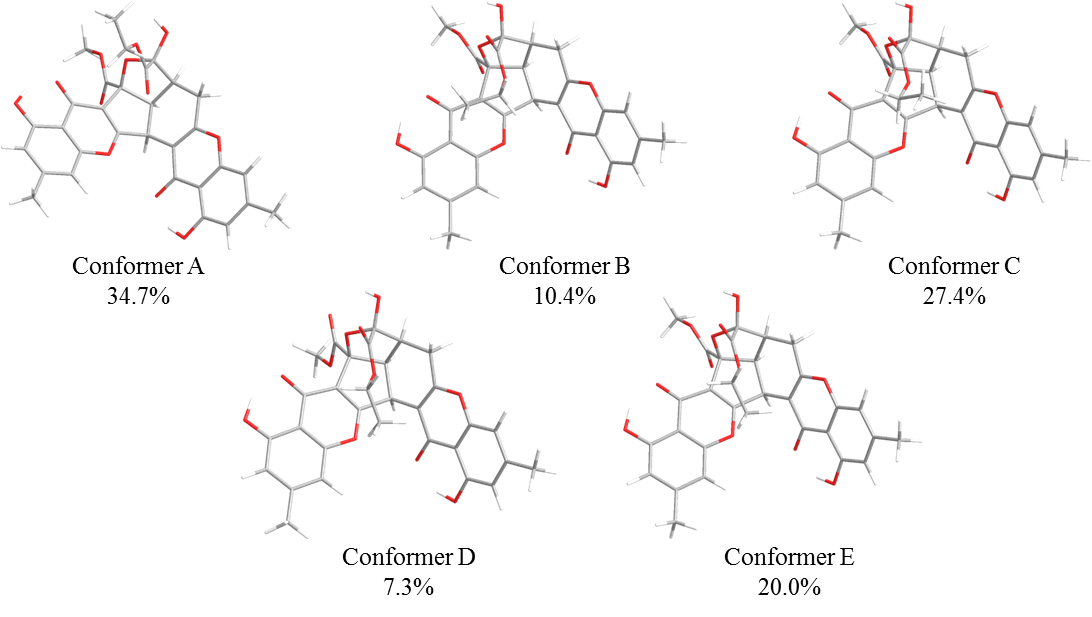 |
| (**B**) |

**Figure S5.** *Cont*.

| **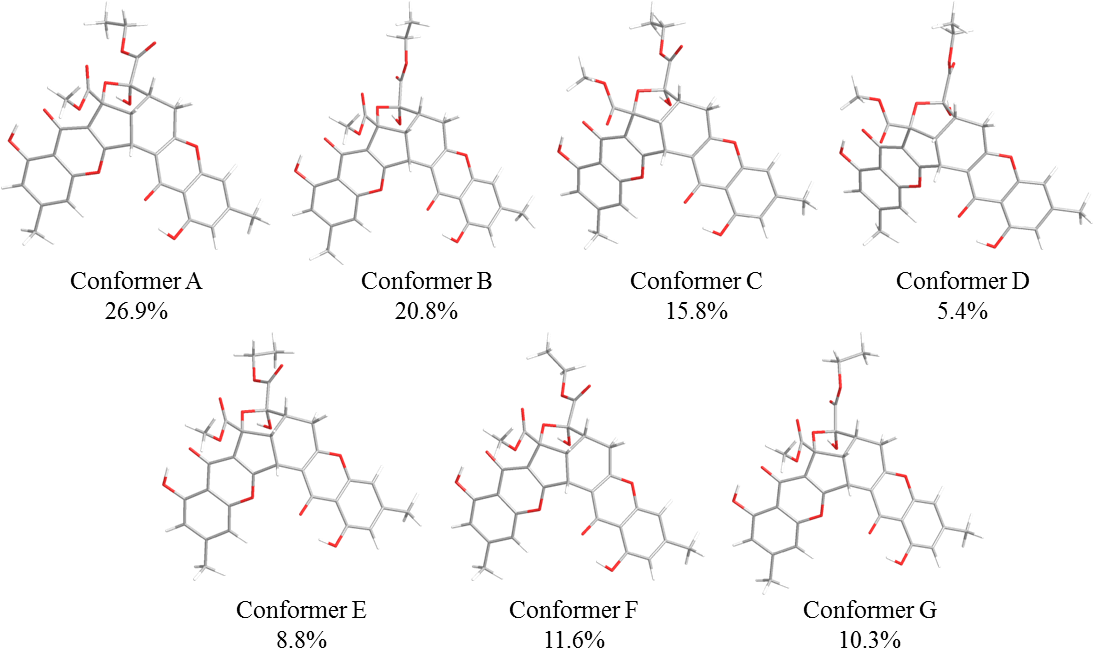** |
| --- |
| (**C**) |
| **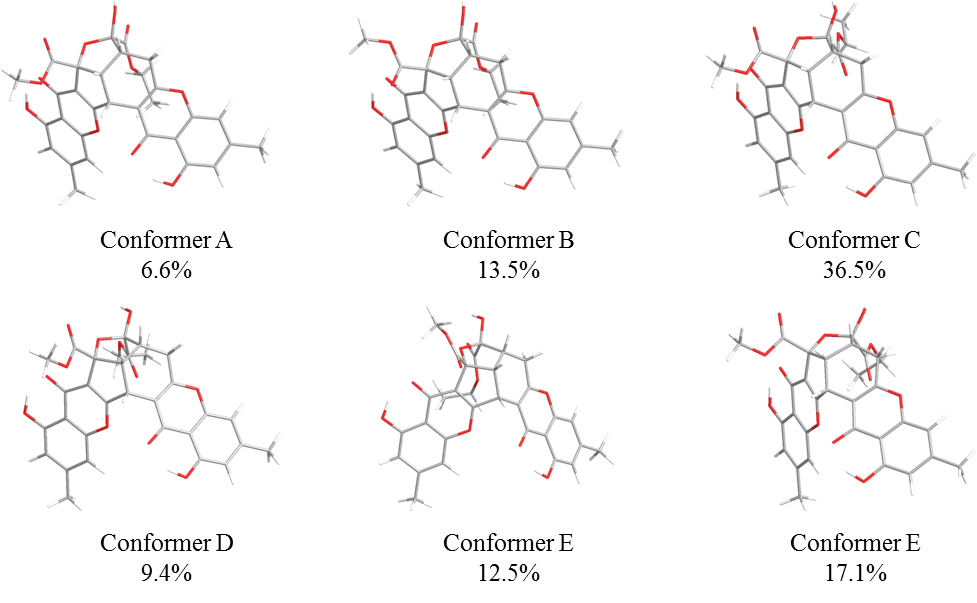** |
| (**D**) |

**Figure S5.** DFT-optimized structures for low-energy conformers of **3a**, **3b**, *ent*-**3a** and *ent*-**3b** for **3** in vacuum at the B3LYP/6-31g(d) level. Conformer populations were calculated using the Gibbs free energy and Boltzmann population at 298 K estimated thereof; (**A**) Low-energy conformers of **3a** (2*S*3*R*4*S*2′*S*3′*S*); (**B**) Low-energy conformers of **3b** (2*S*3*R*4*S*2′*R*3′*S*); (**C**) Low-energy conformers of *ent*-**3a** (2*R*3*S*4*R*2′*R*3′*R*); (**D**) Low-energy conformers of *ent*-**3b** (2*R*3*S*4*R*2′*S*3′*R*).


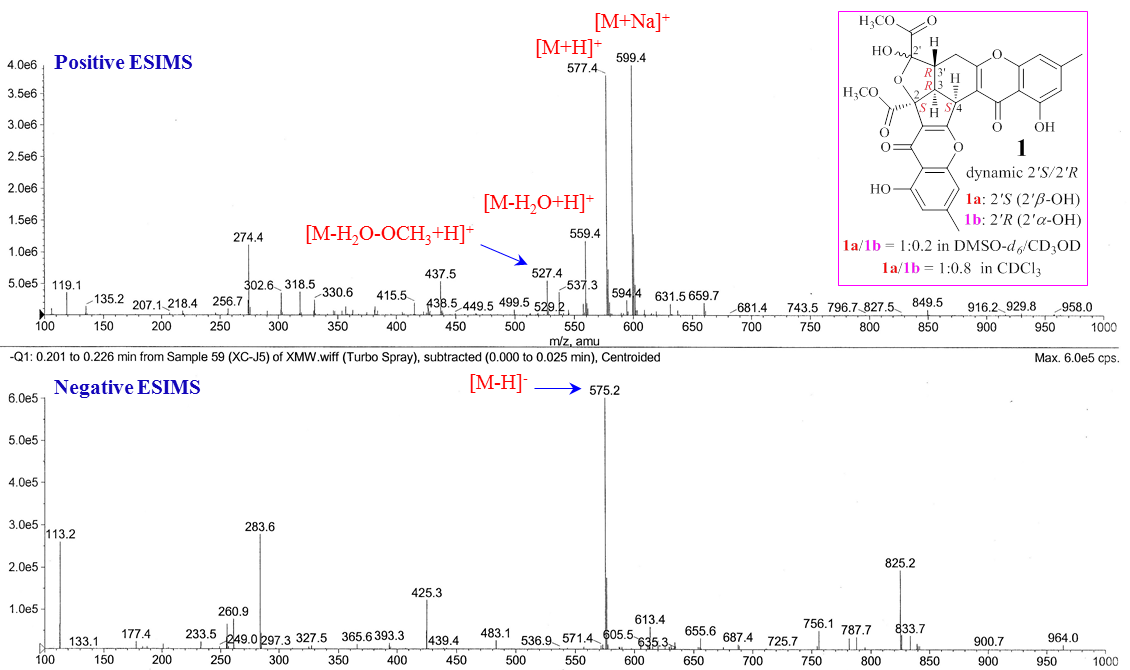


**Figure S6.** Positive and negative ESIMS spectra of **1**.

**
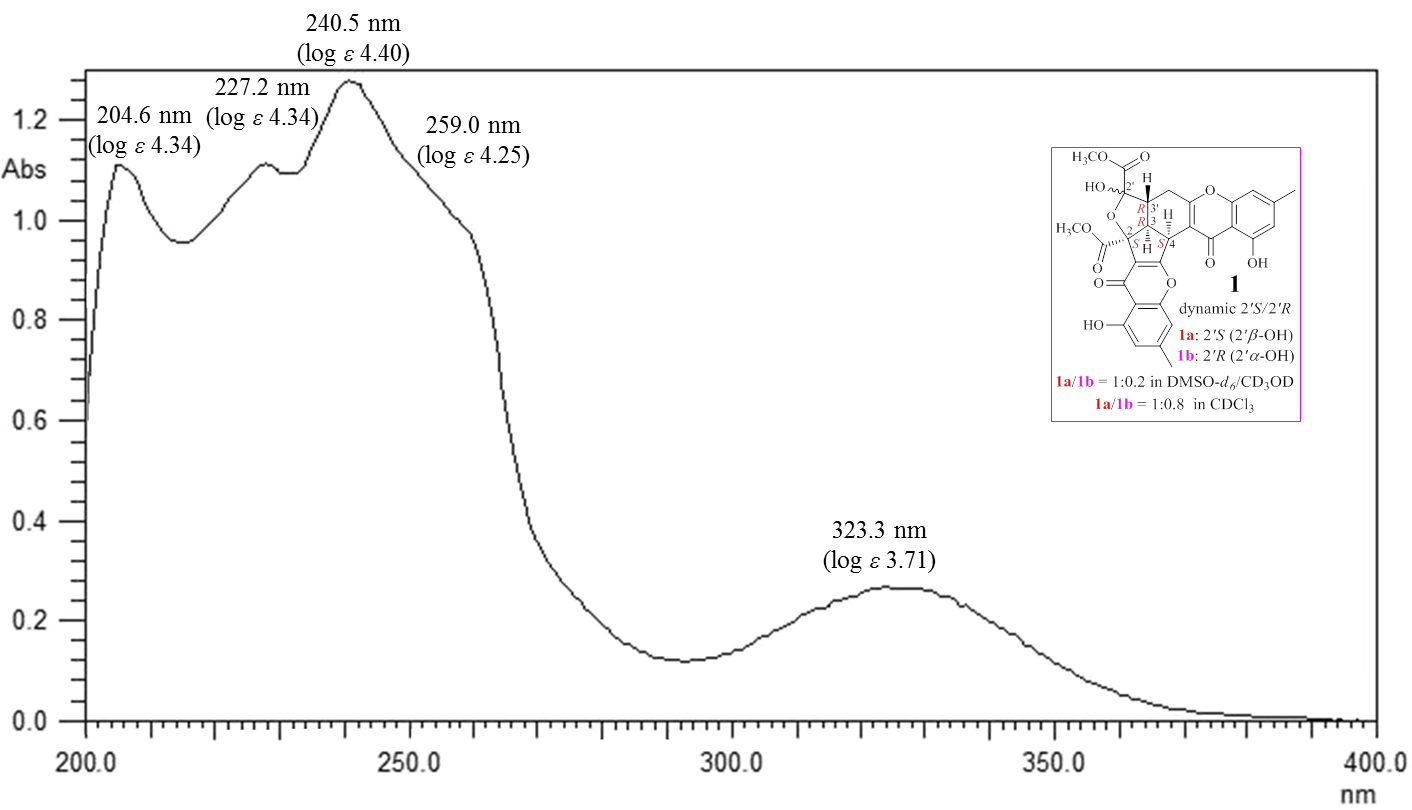
**

**Figure S7.** UV Spectrum of **1** in MeOH.

**
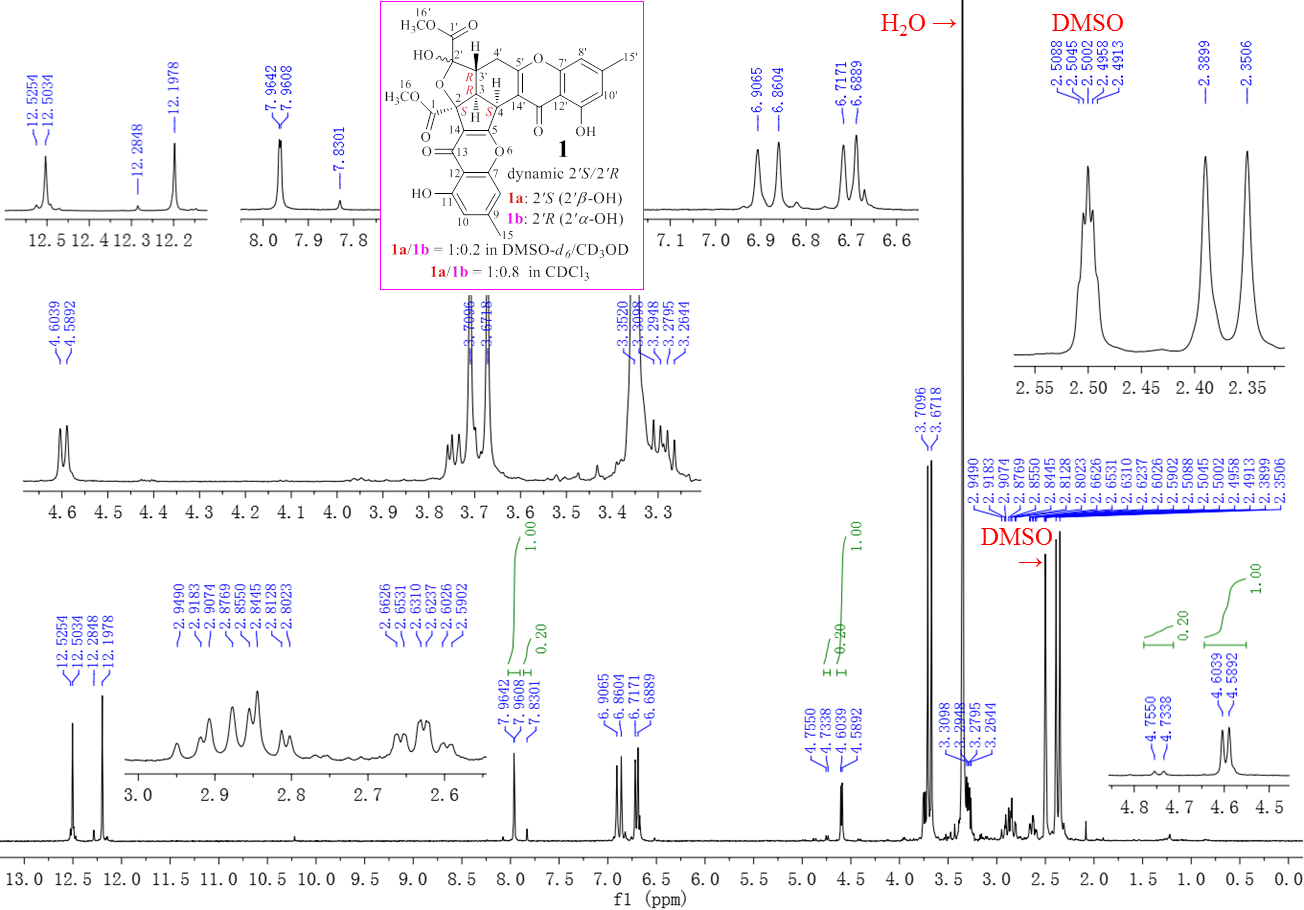
**

**Figure S8.** 400 MHz ^1^H NMR Spectrum of **1** in DMSO-*d*_6_.

**
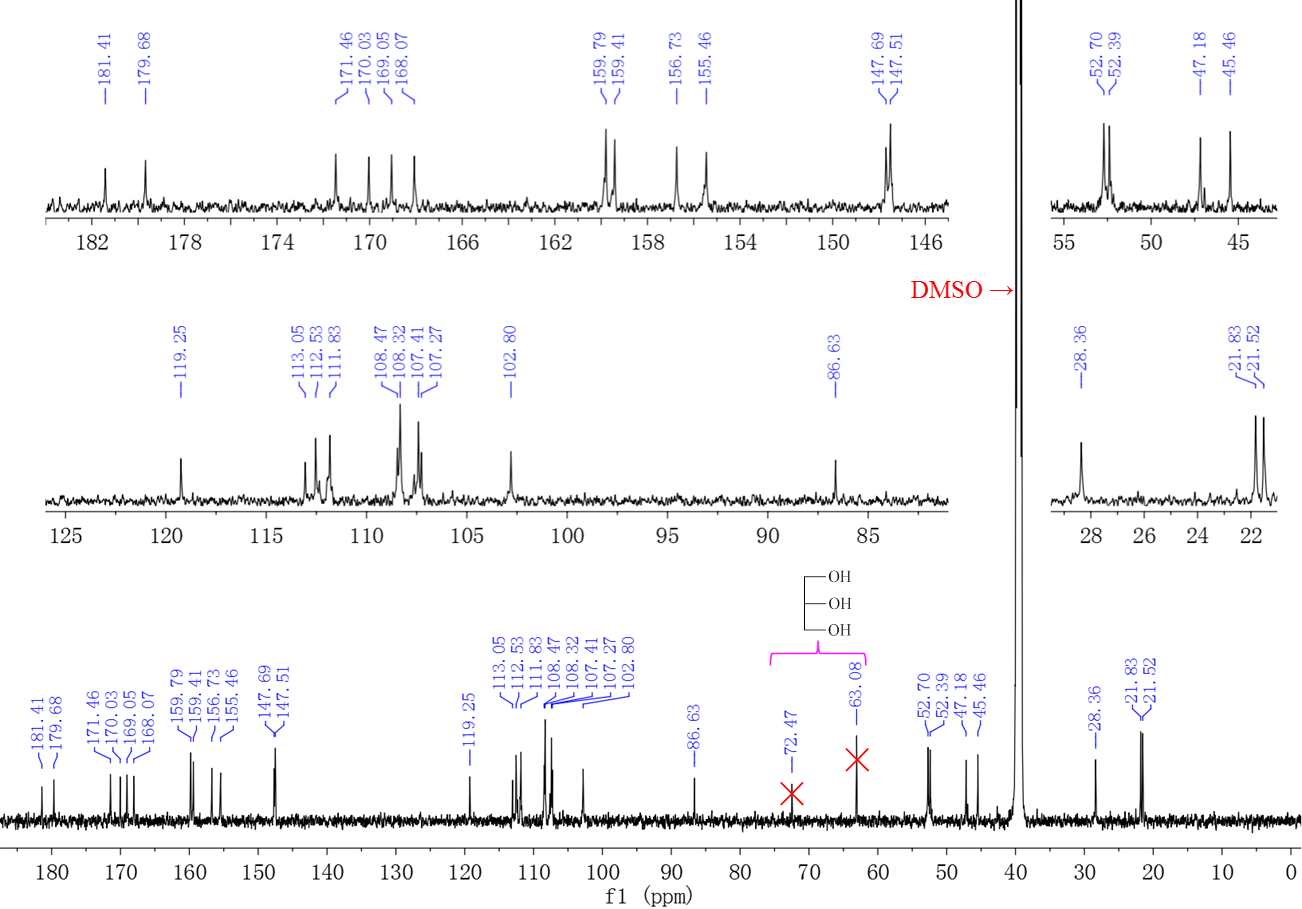
**

**Figure S9.** 150 MHz ^13^C NMR spectrum of **1** in DMSO-*d*_6_.

**
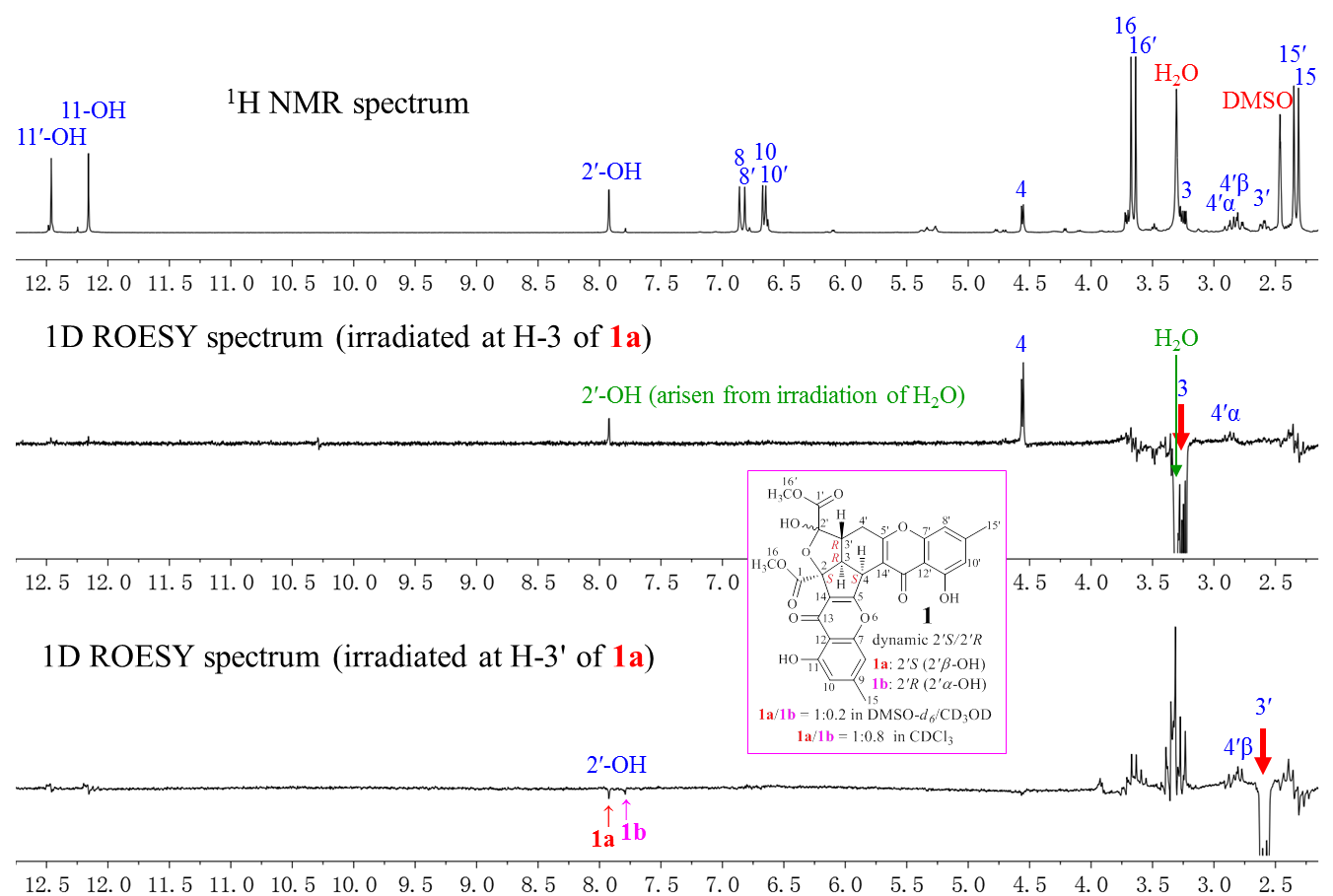
**

**Figure S10.** 400 MHz ^1^H NMR and 1D ROESY spectra of **1** in DMSO-*d*_6_.

**
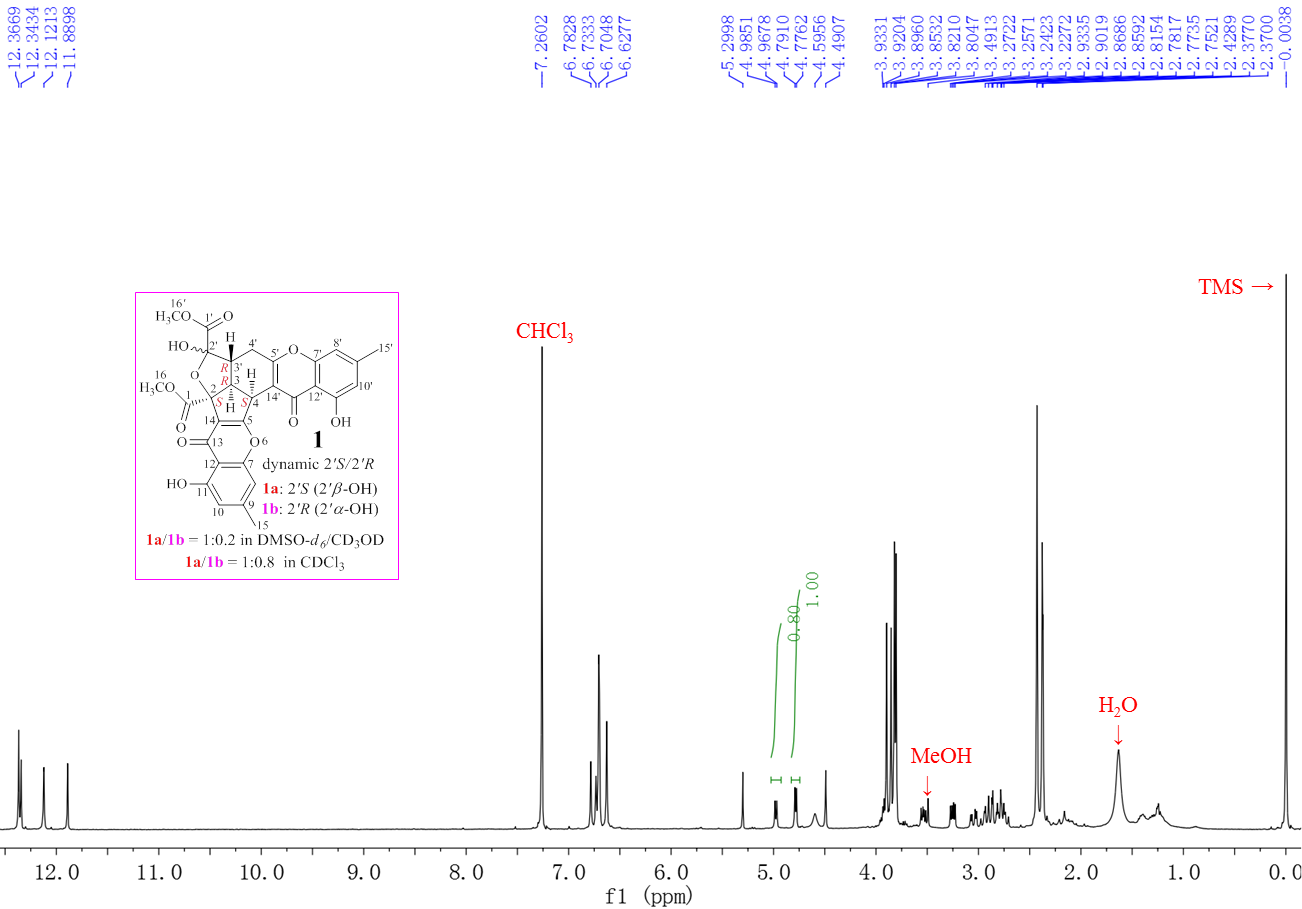
**

**Figure S11.** 400 MHz ^1^H NMR spectrum of **1** in CDCl_3_.

**
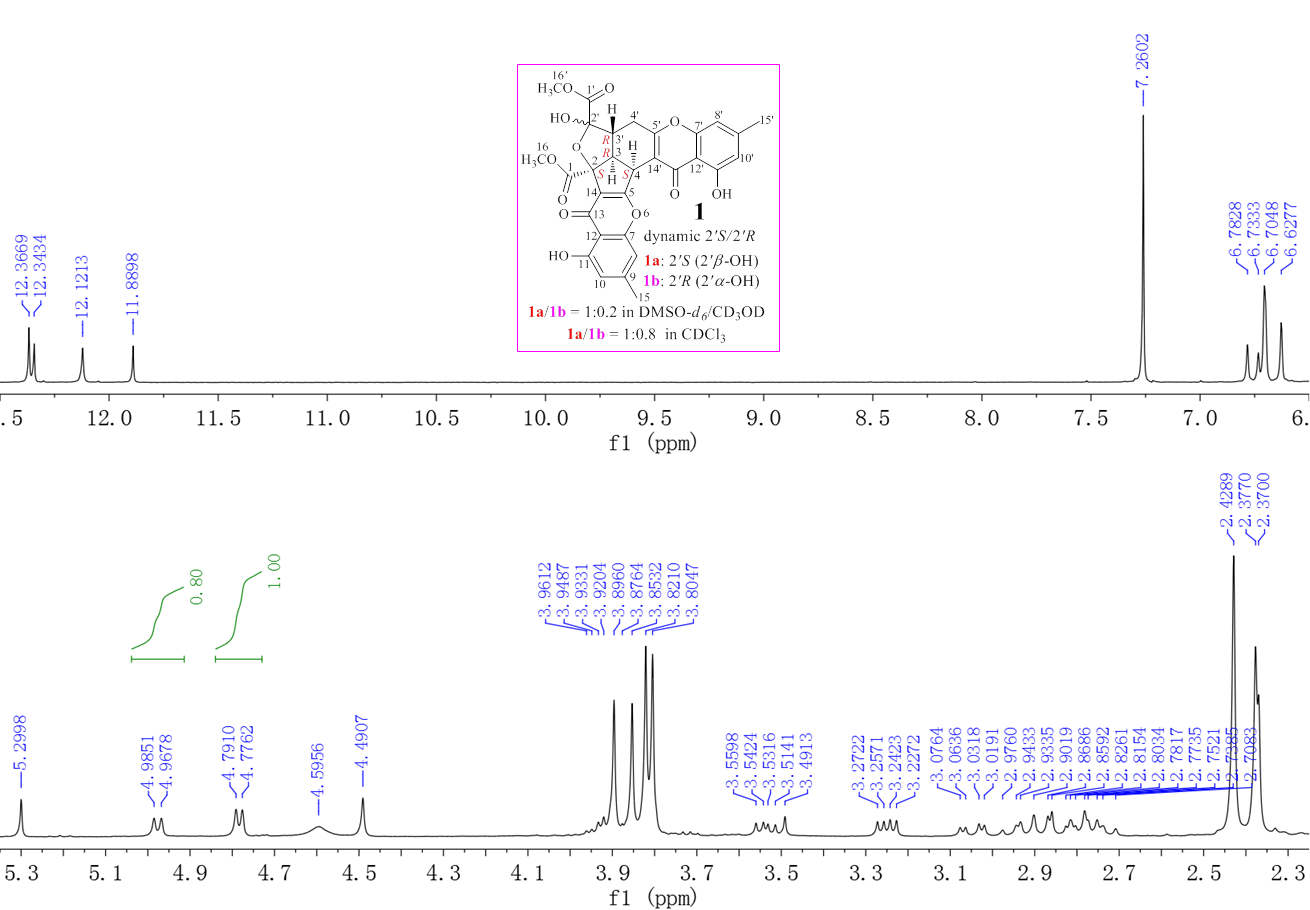
**

**Figure S12.** Expanded 400 MHz ^1^H NMR spectrum of **1** in CDCl_3_.


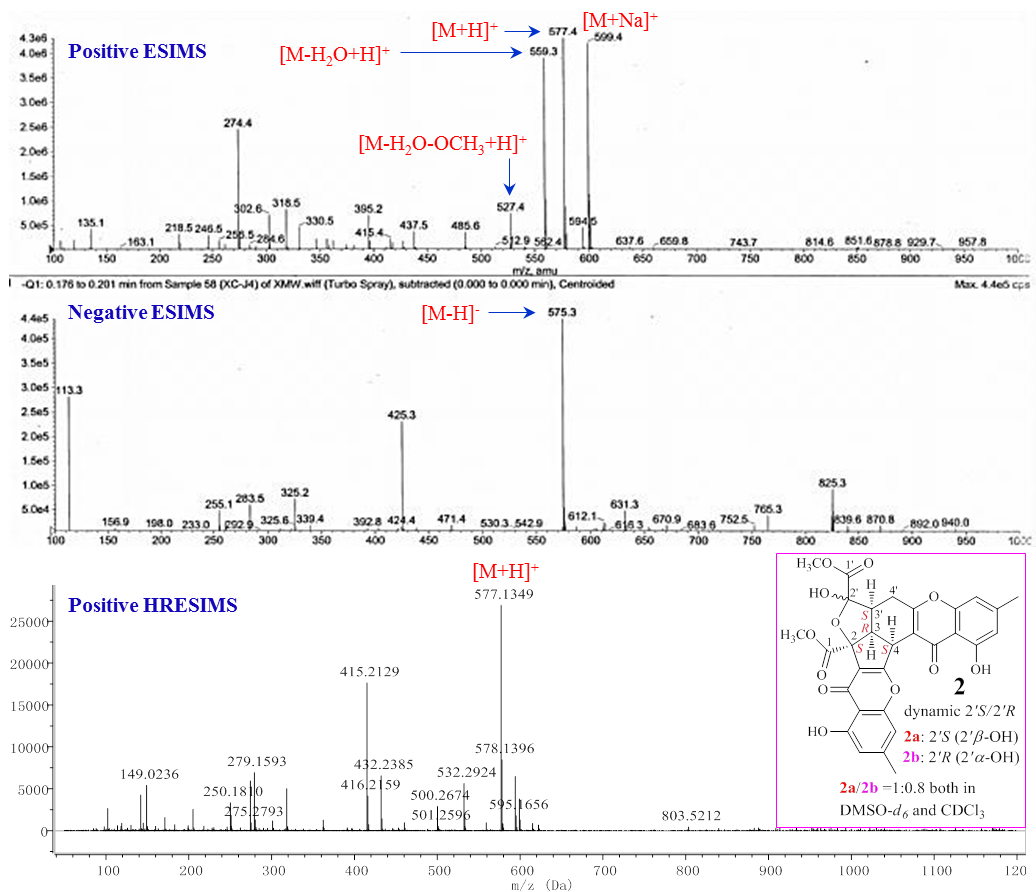


**Figure S13.** ESIMS and HRESIMS Spectra of **2**.


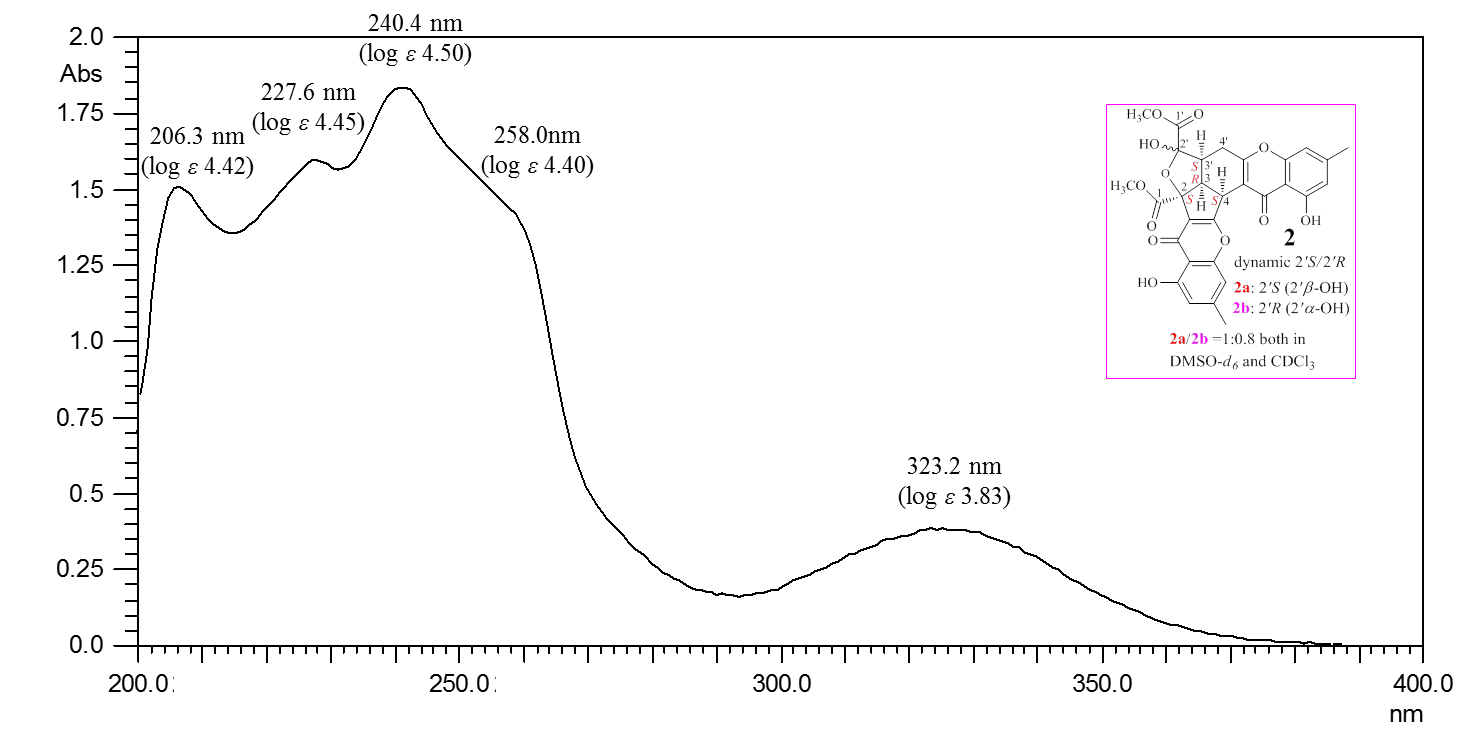


**Figure S14.** UV spectrum of **2** in MeOH.


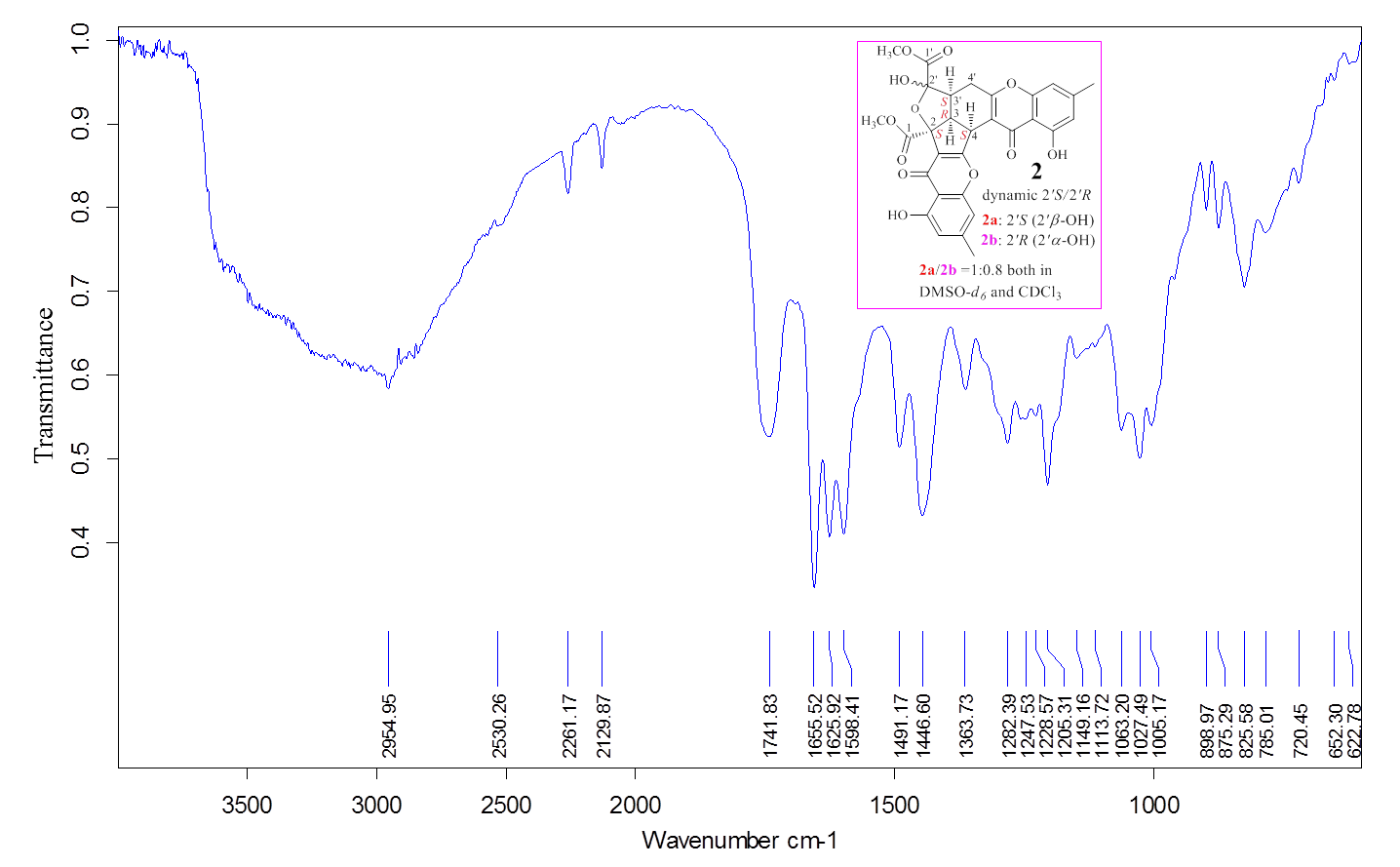


**Figure S15.** IR spectrum of **2**.


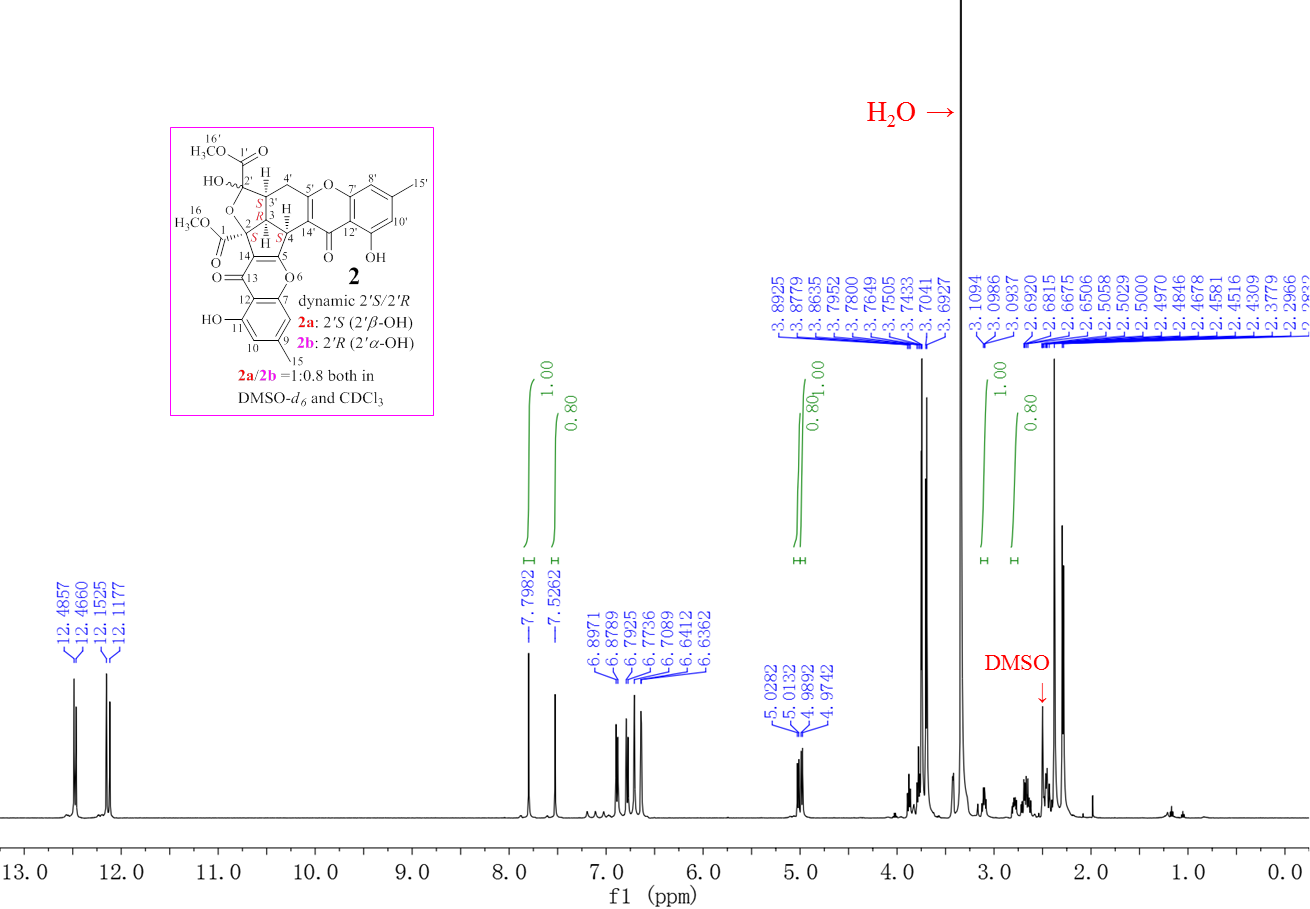


**Figure S16.** 600 MHz ^1^H NMR spectrum of **2** in DMSO-*d*_6_.


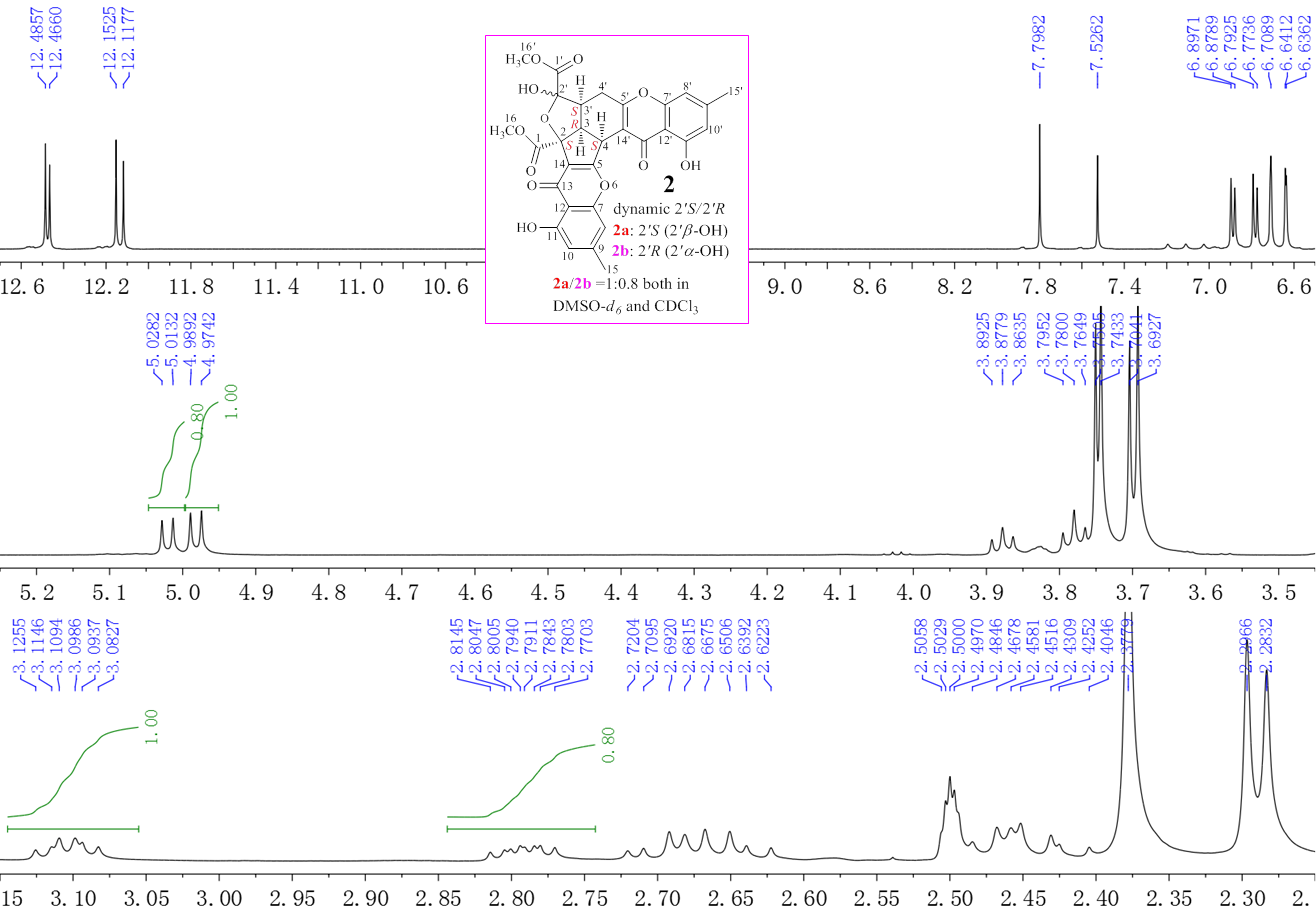


**Figure S17.** Expanded 600 MHz ^1^H NMR spectrum of **2** in DMSO-*d*_6_.


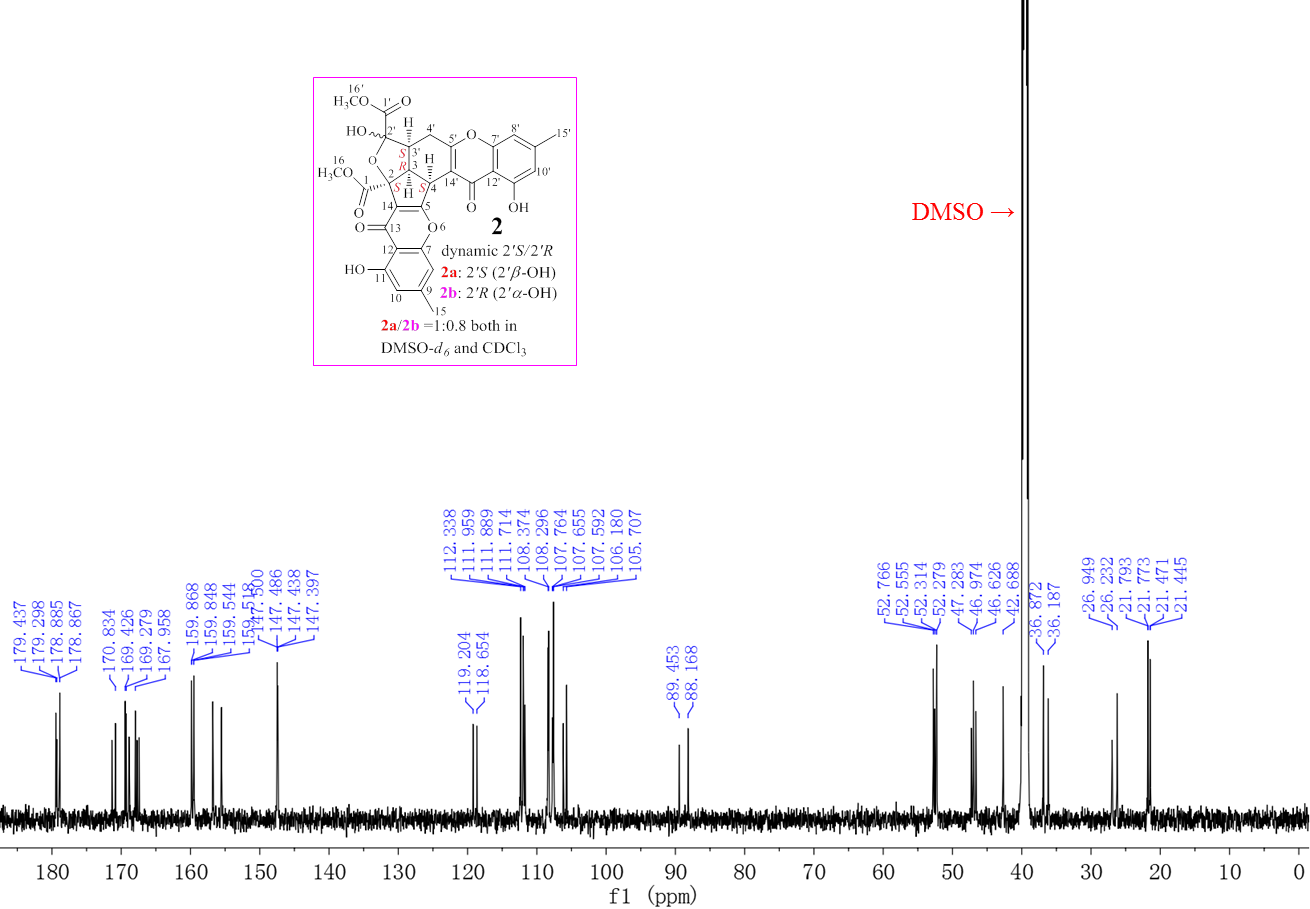


**Figure S18.** 150 MHz ^13^C NMR spectrum of **2** in DMSO-*d*_6_.


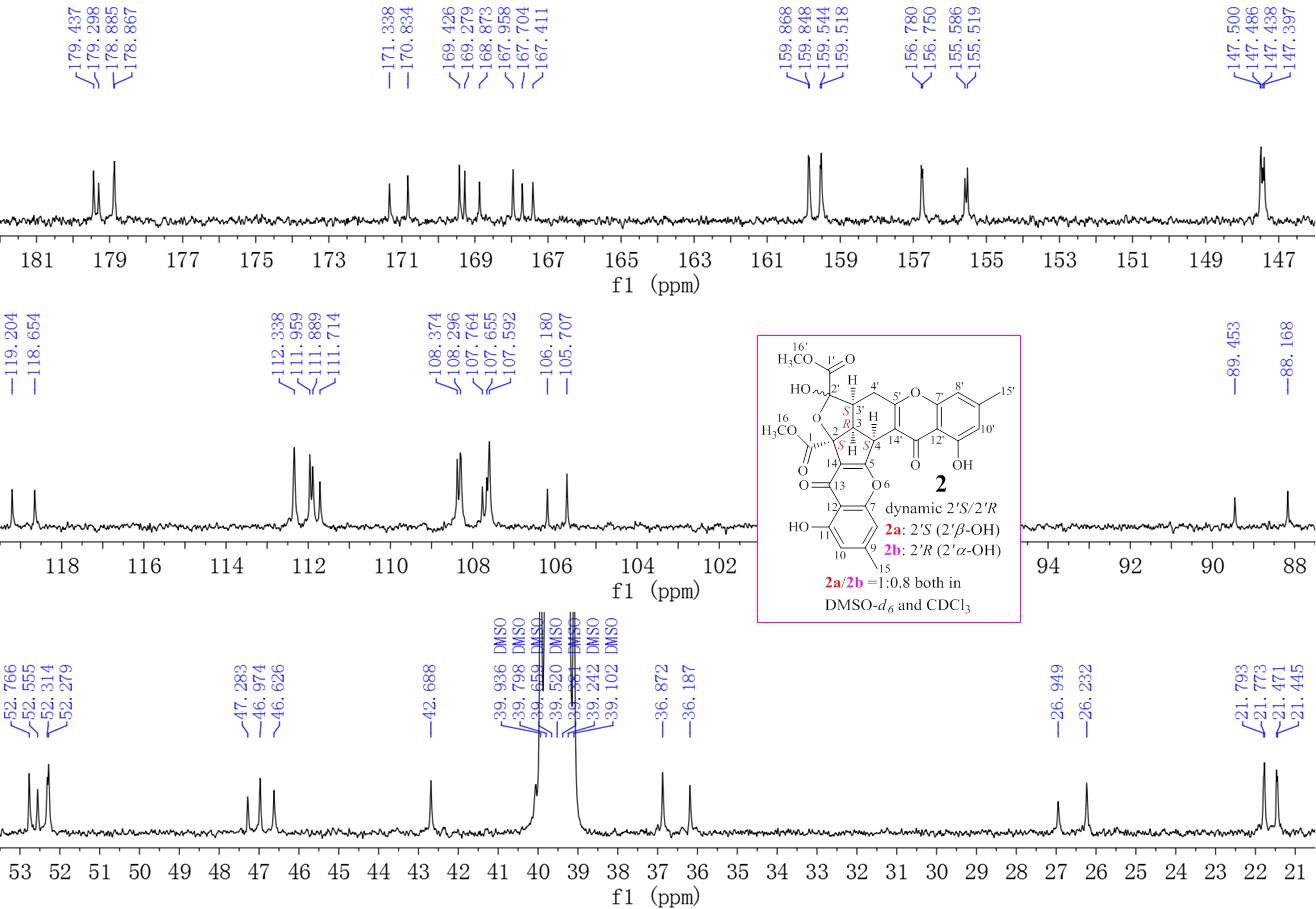


**Figure S19.** Expanded 150 MHz ^13^C NMR spectrum of **2** in DMSO-*d*_6_.


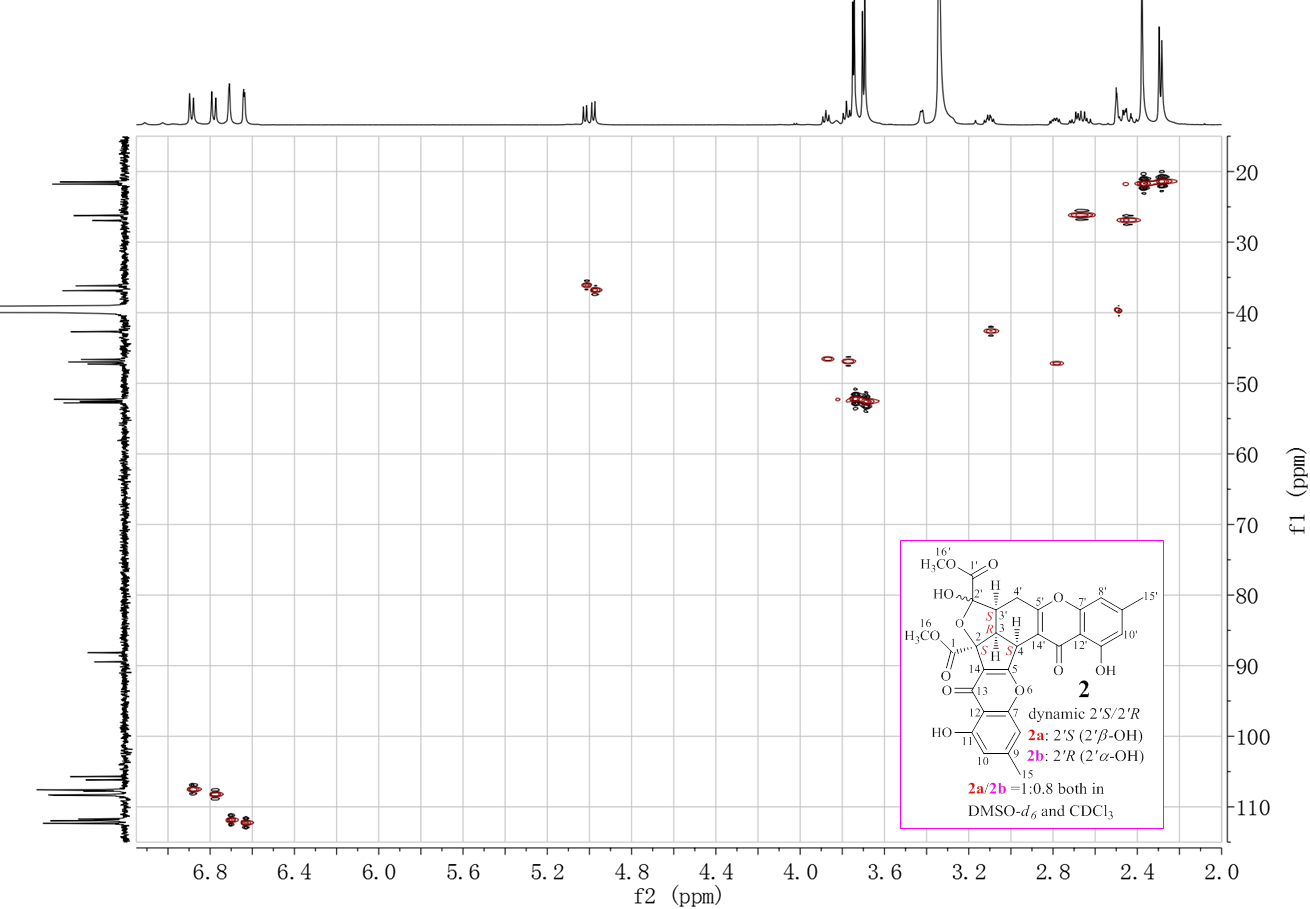


**Figure S20.** 600 MHz HMQC spectrum of **2** in DMSO-*d*_6_.


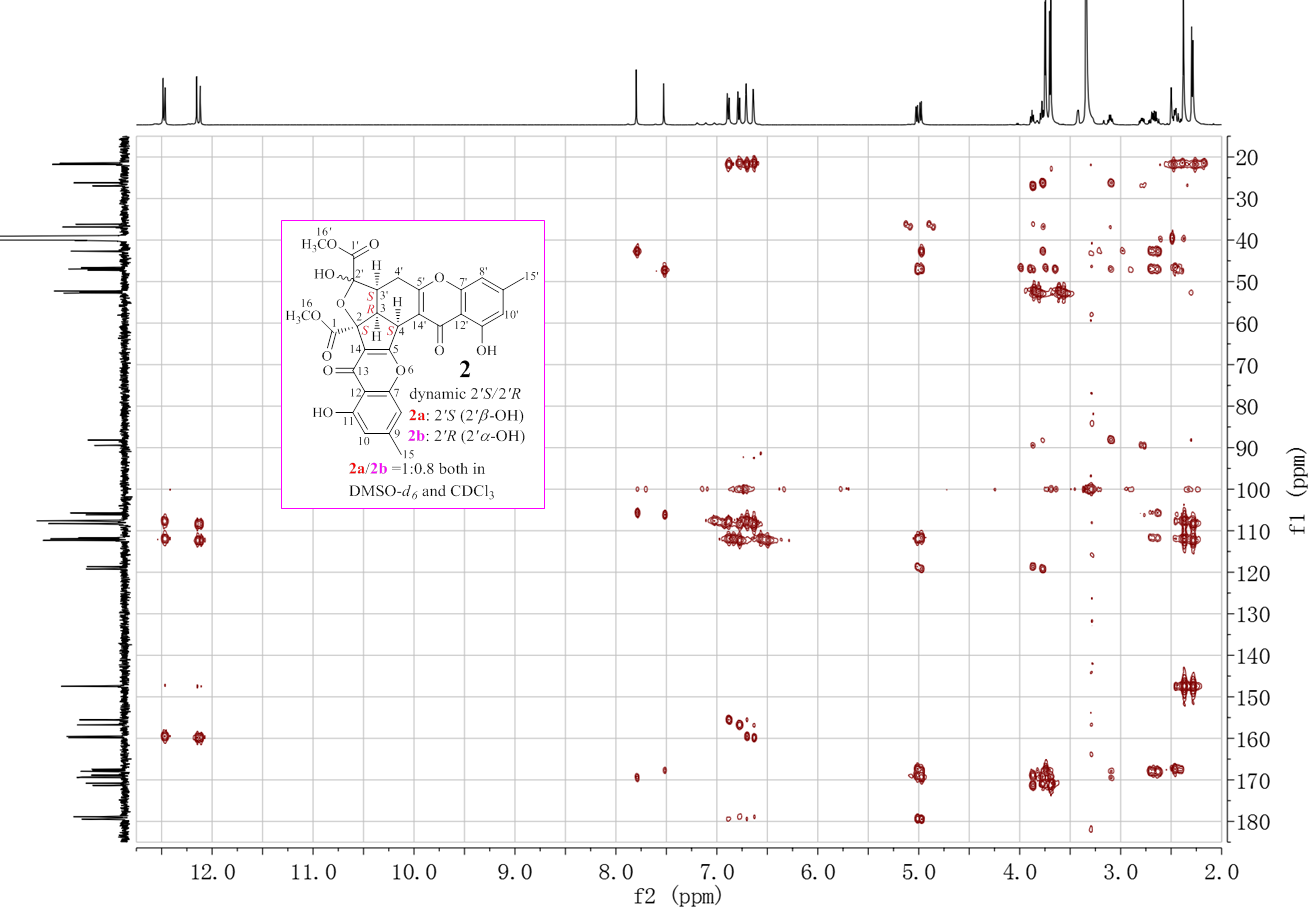


**Figure S21.** 600MHz HMBC spectrum of **2** in DMSO-*d*_6_.


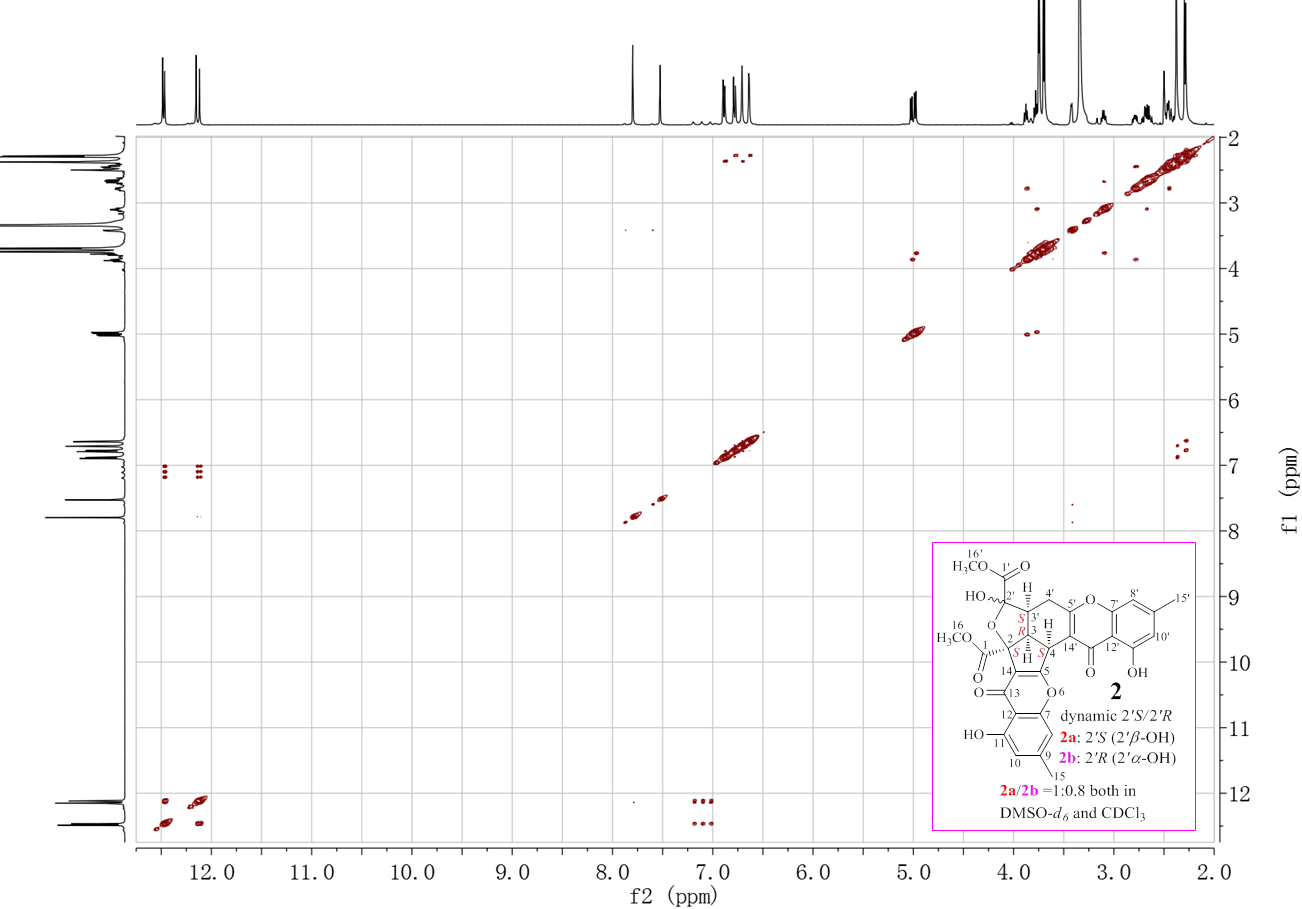


**Figure S22.** 600 MHz NOESY spectrum of **2** in DMSO-*d*_6_.


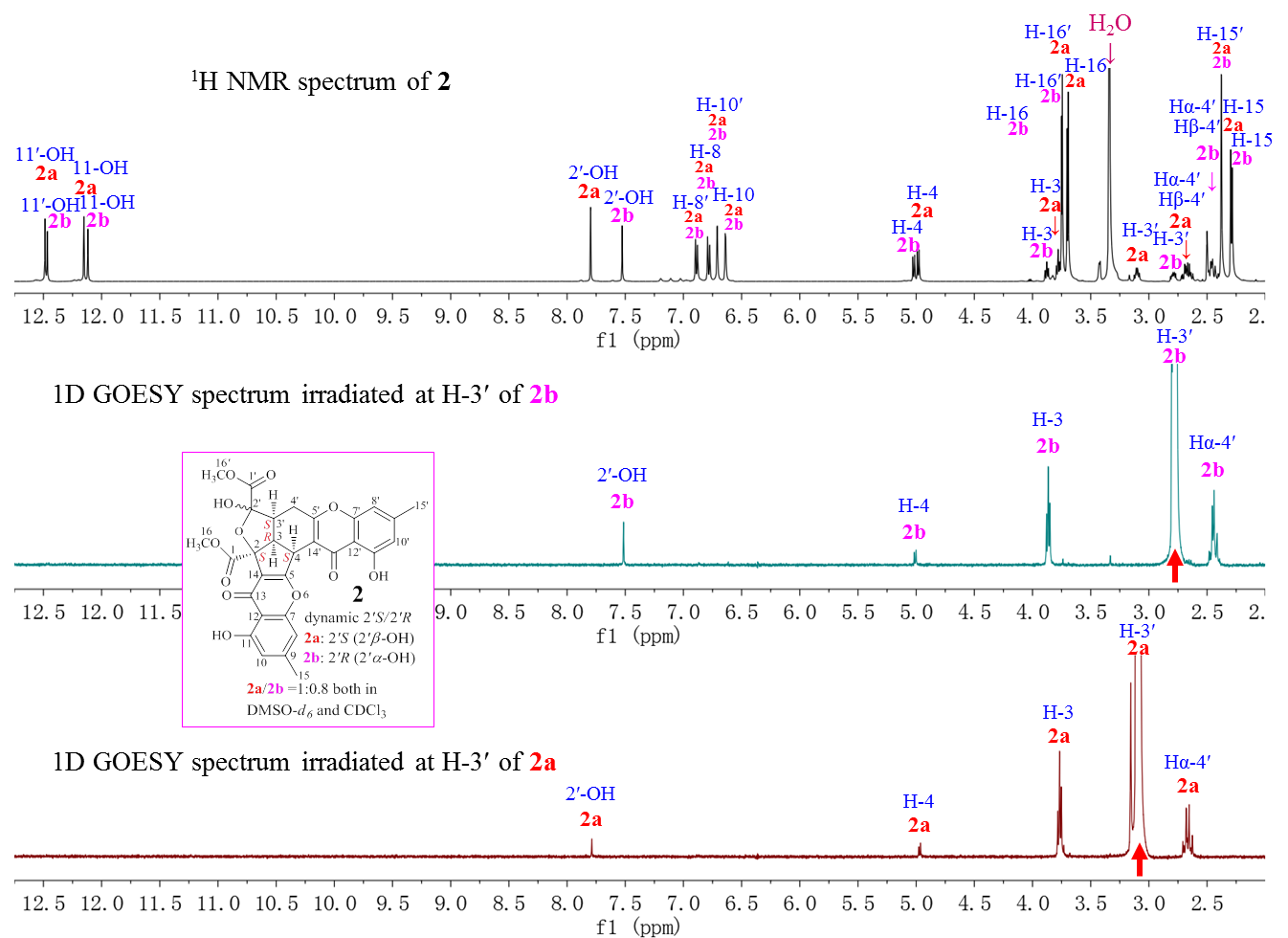


**Figure S23.** 600 MHz ^1^H and 1D GOESY spectra of **2** in DMSO-*d*_6_ (irradiated at H-3′).


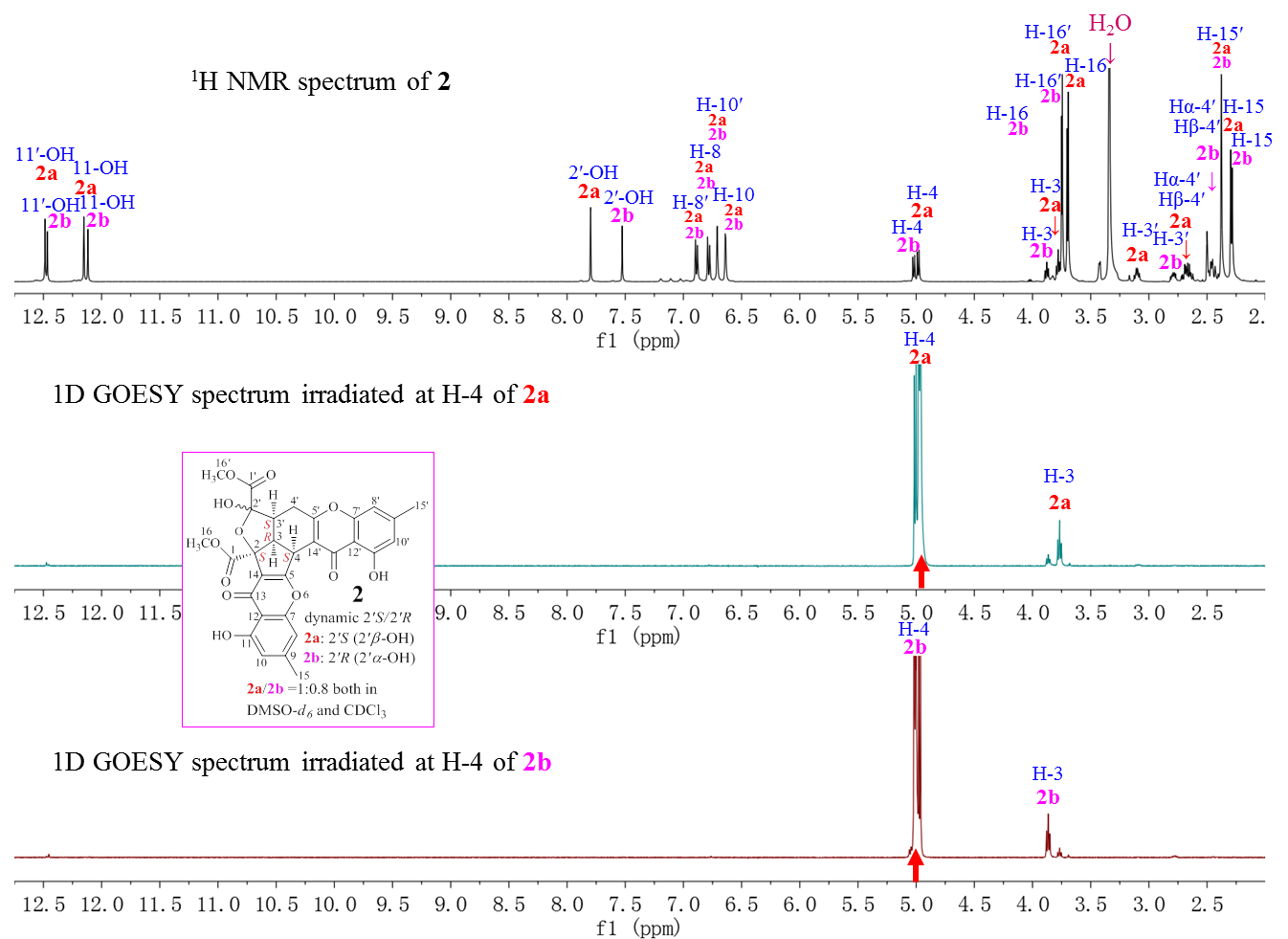


**Figure S24.** 600 MHz ^1^H and 1D GOESY spectra of **2** in DMSO-*d*_6_ (irradiated at H-4).


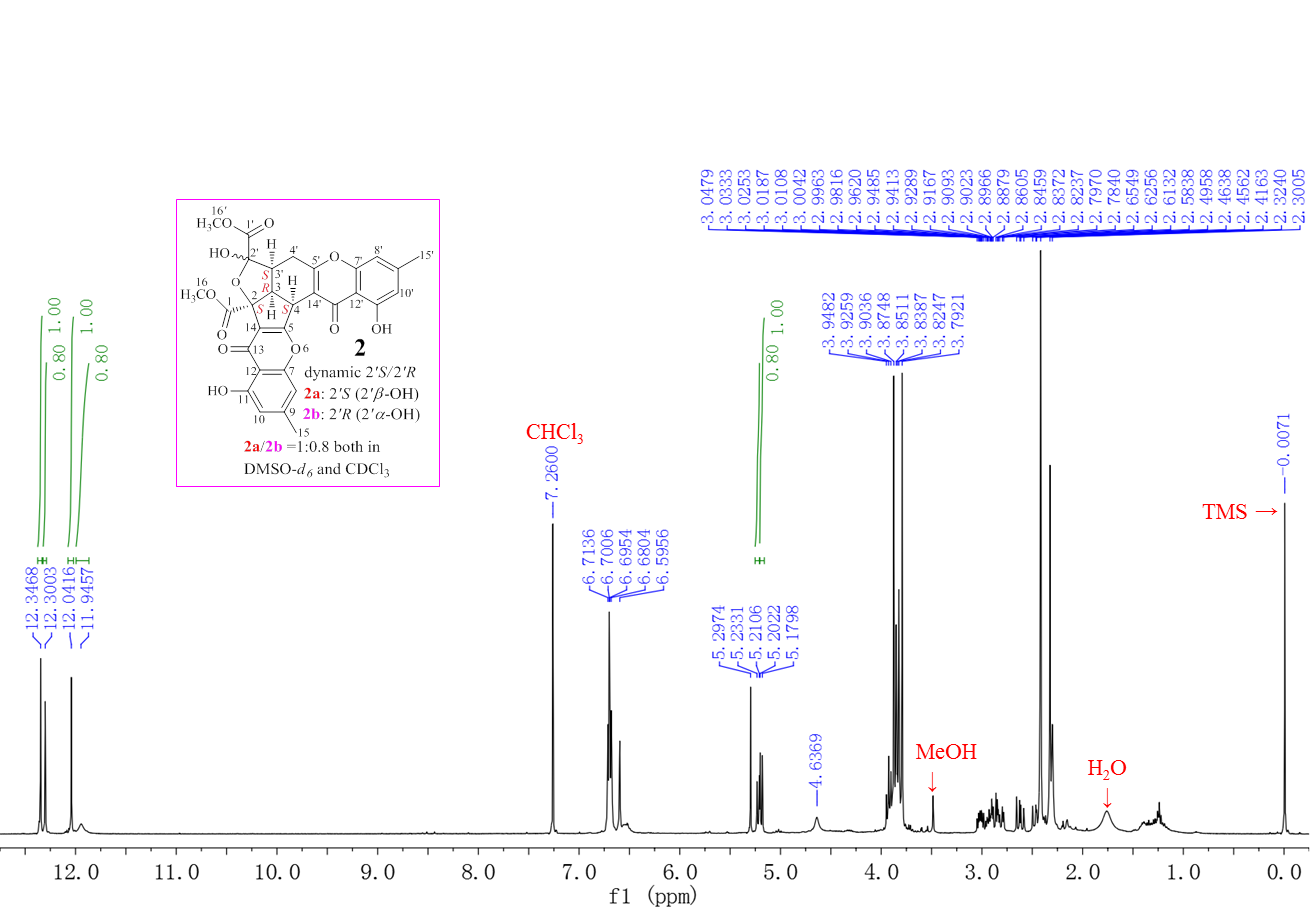


**Figure S25.** 400 MHz ^1^H NMR spectrum of **2** in CDCl_3_.


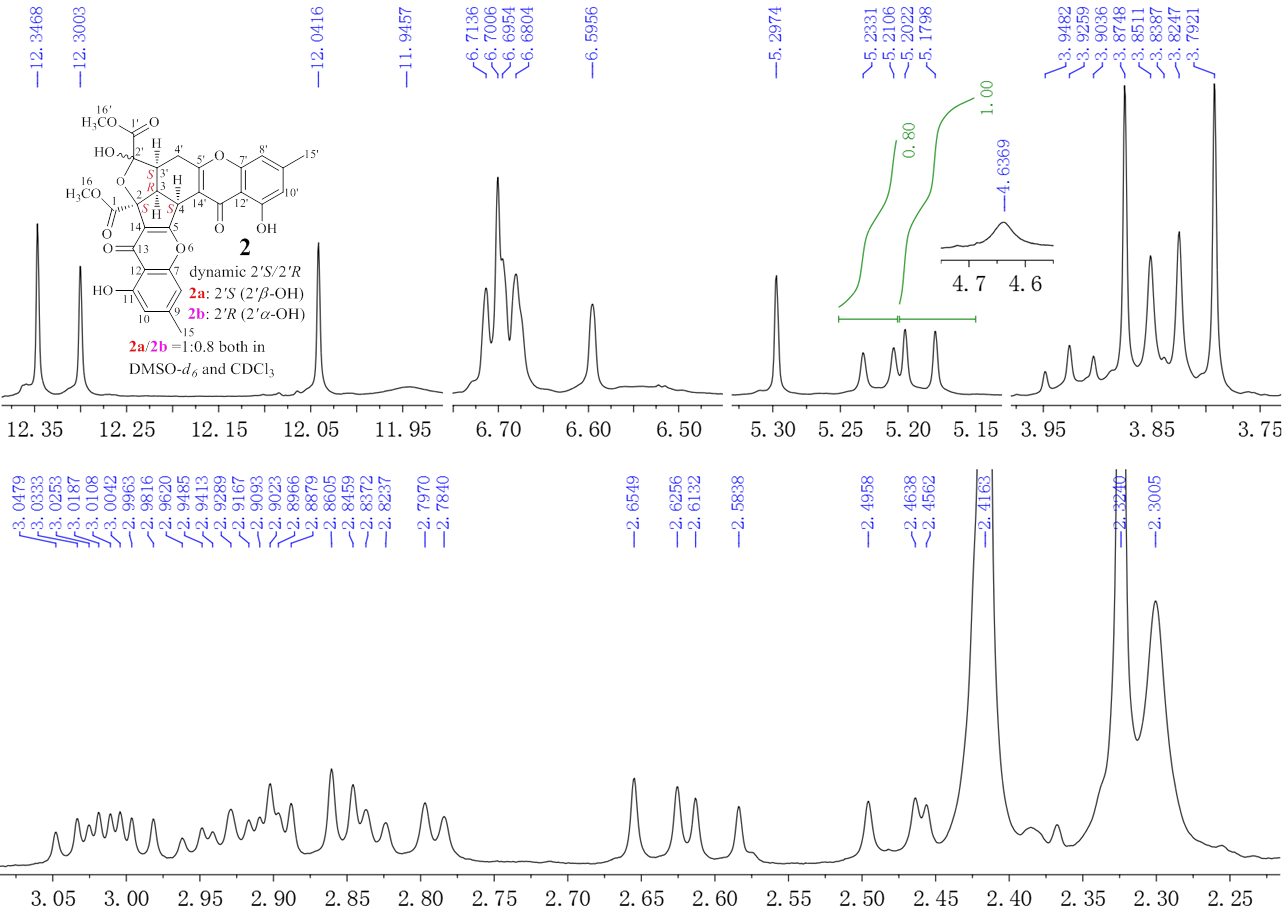


**Figure S26.** Expanded 400 MHz ^1^H NMR spectrum of **2** in CDCl_3_.


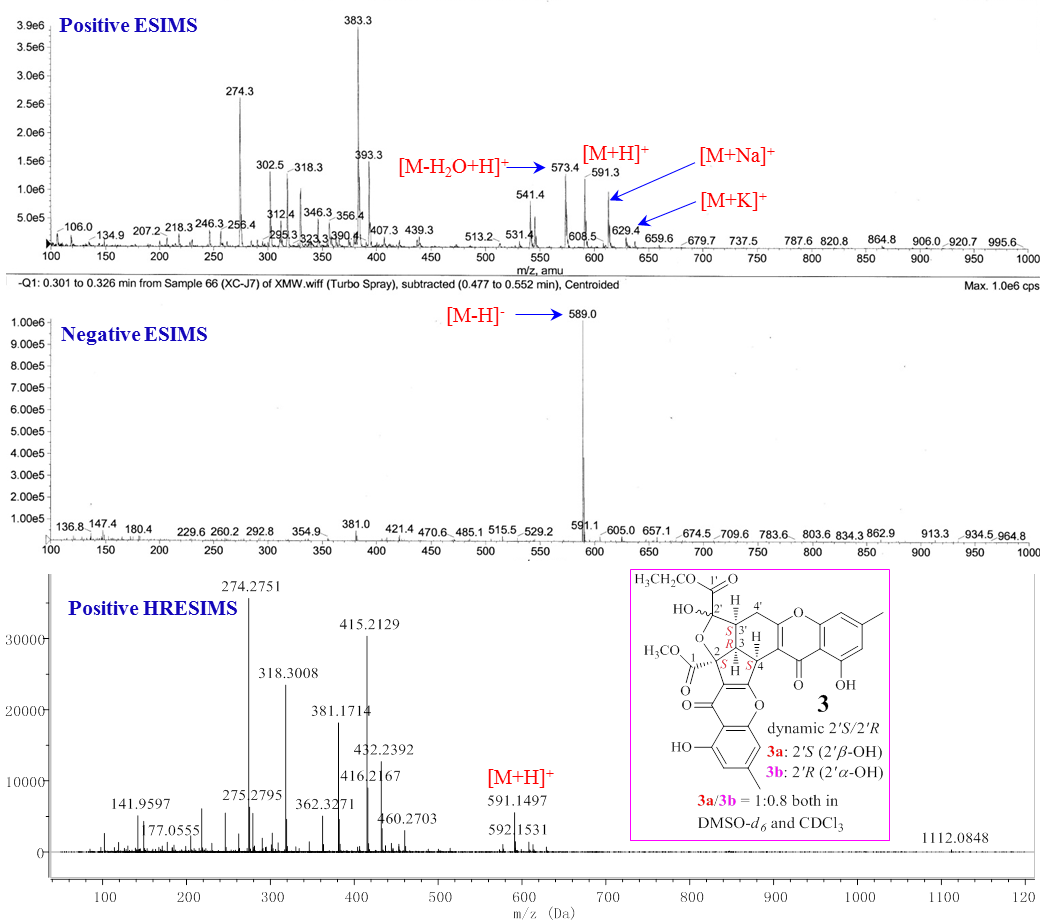


**Figure S27.** ESIMS and HRESIMS spectra of **3**.


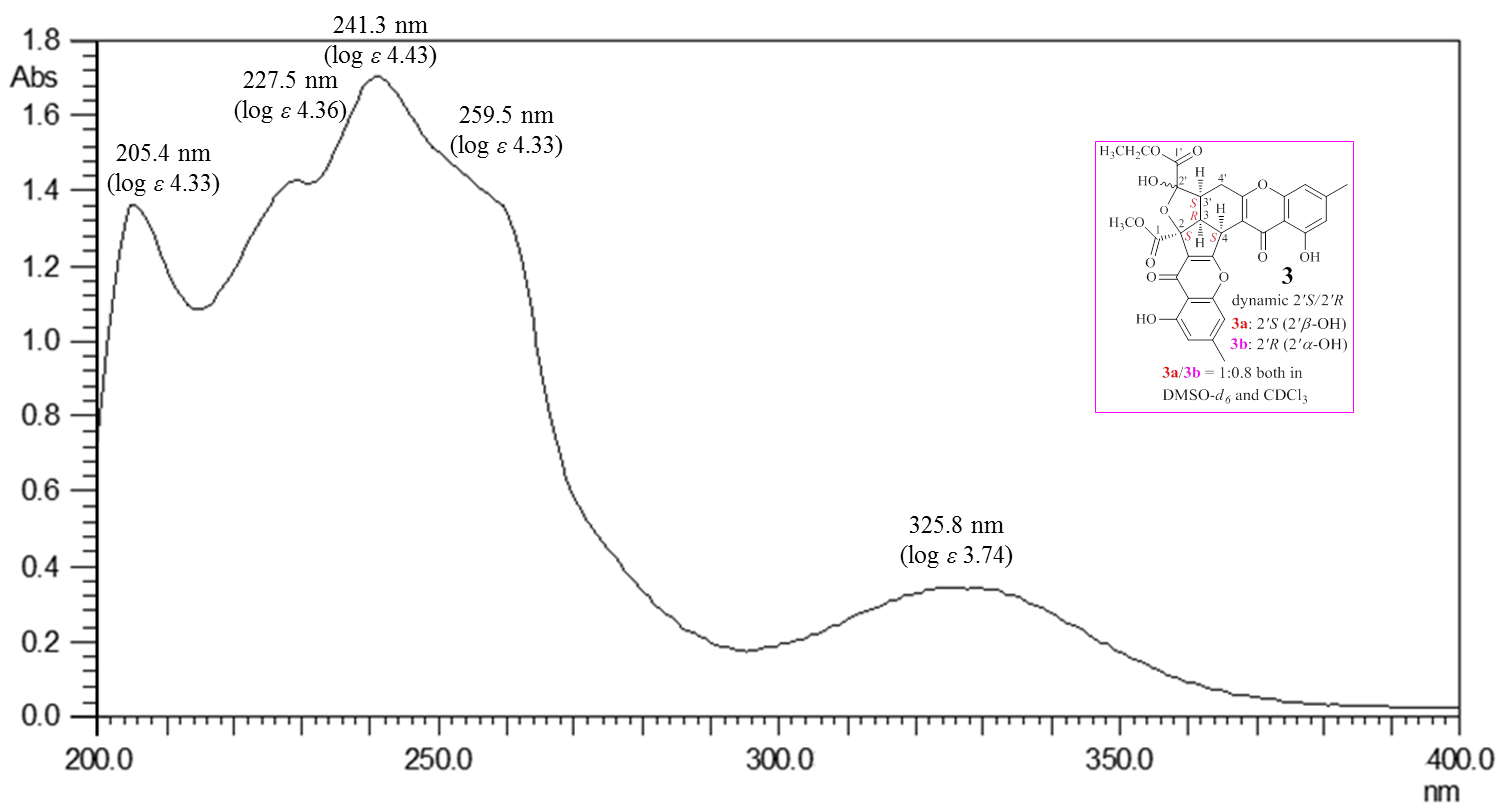


**Figure S28.** UV spectrum of **3** in MeOH.


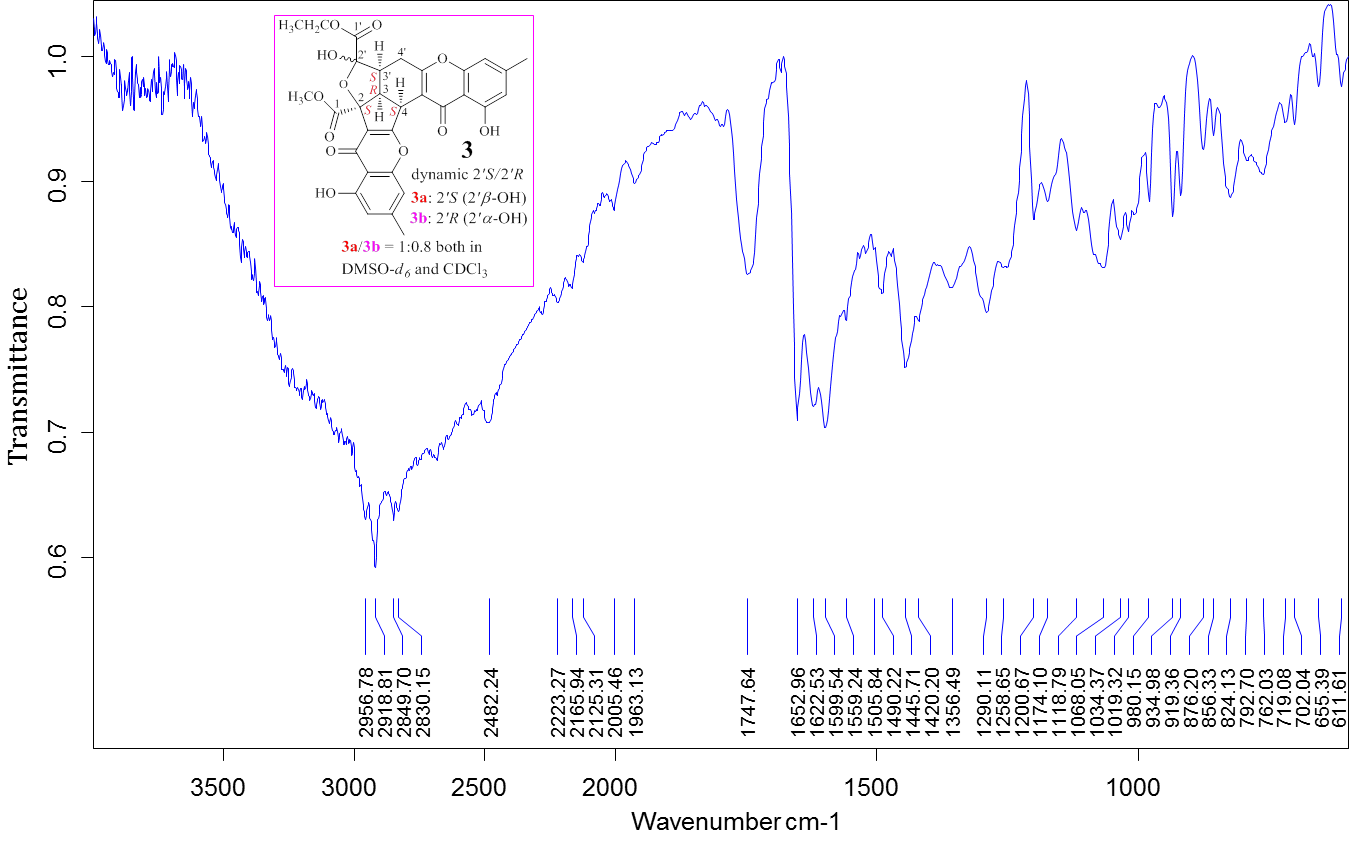


**Figure S29.** IR spectrum of **3**.


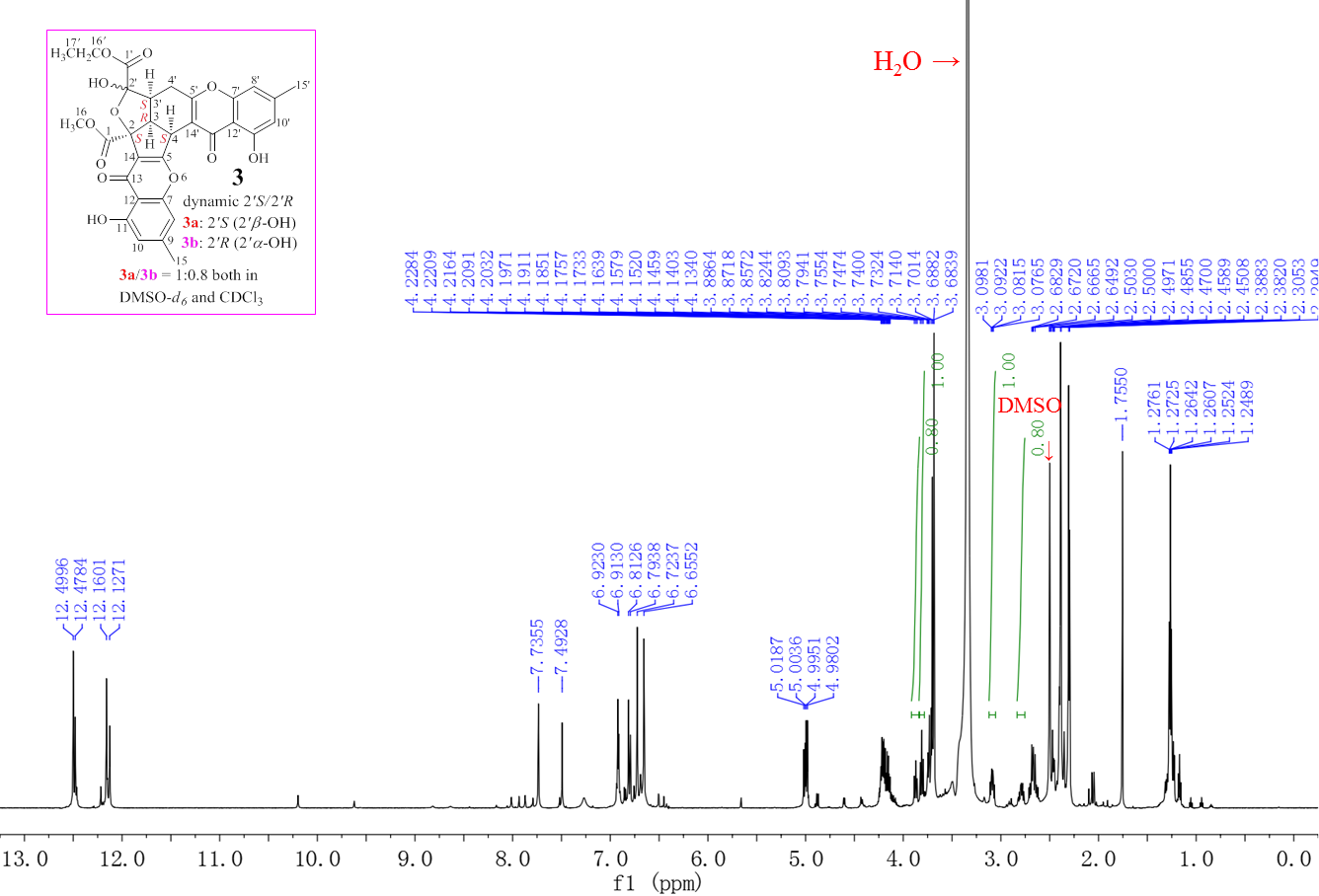


**Figure S30.** 600 MHz ^1^H NMR spectrum of **3** in DMSO-*d*_6_.


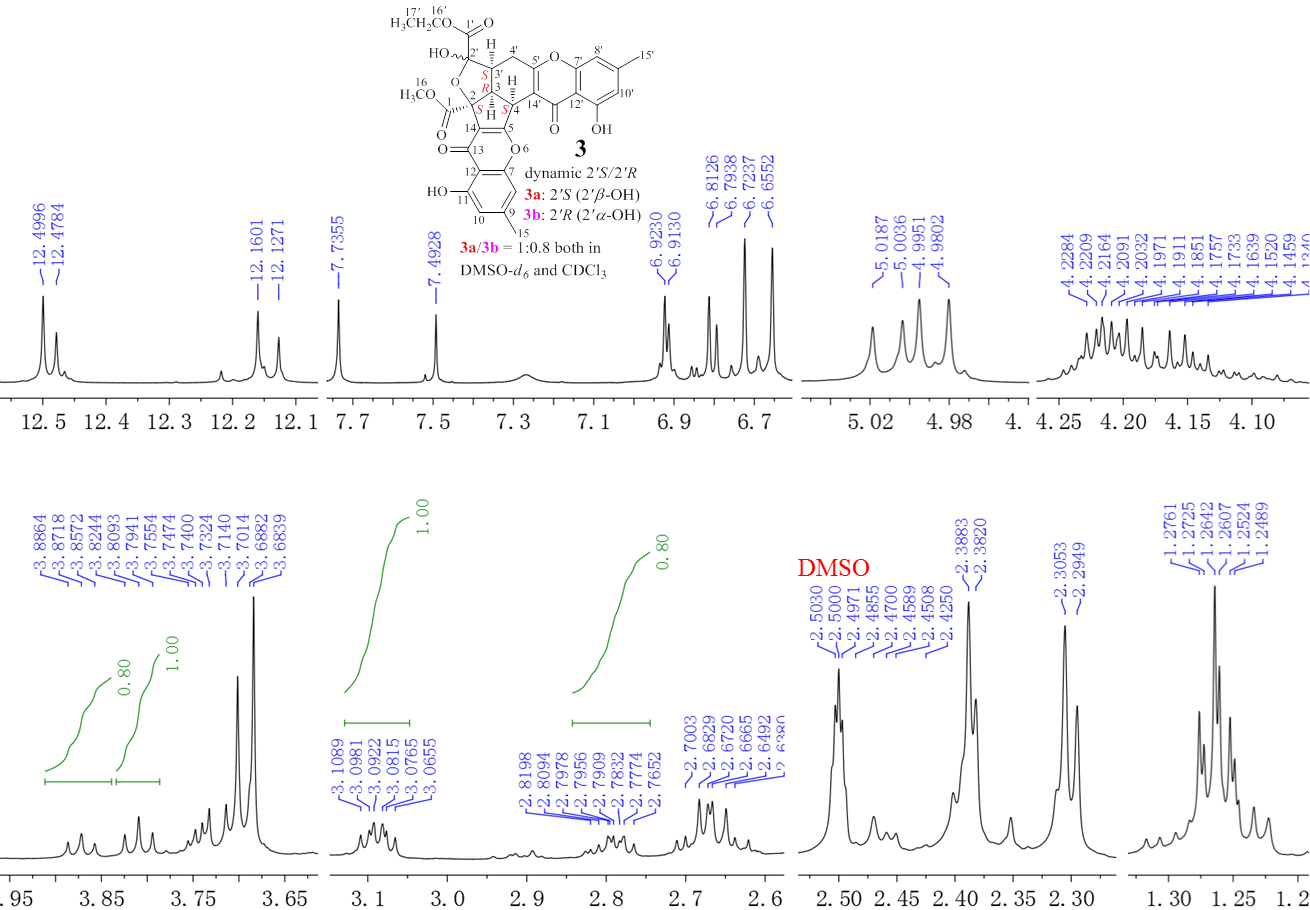


**Figure S31.** Expanded 600 MHz ^1^H NMR spectrum of **3** in DMSO-*d*_6_.


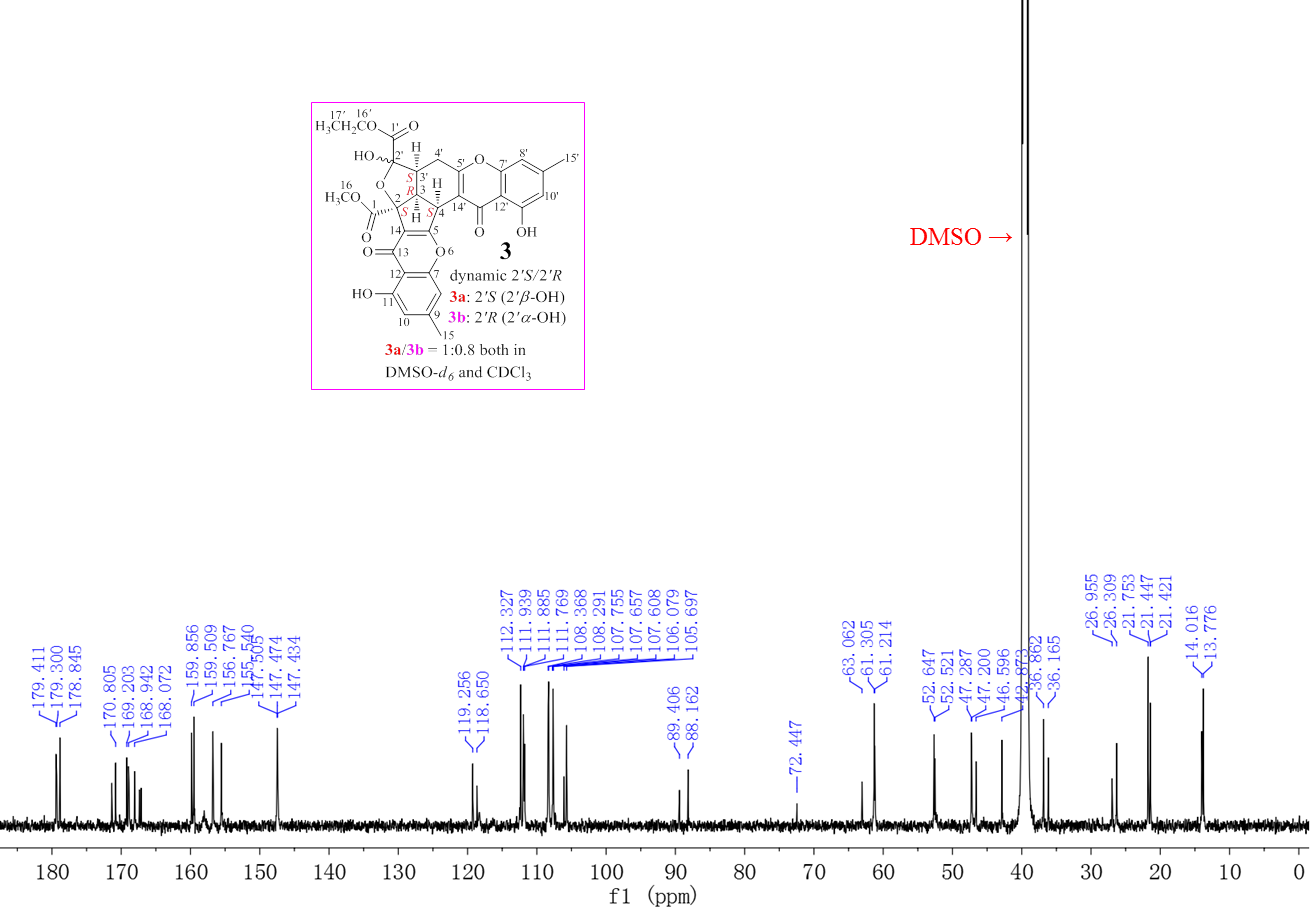


**Figure S32.** 150 MHz ^13^C NMR spectrum of **3** in DMSO-*d*_6_.


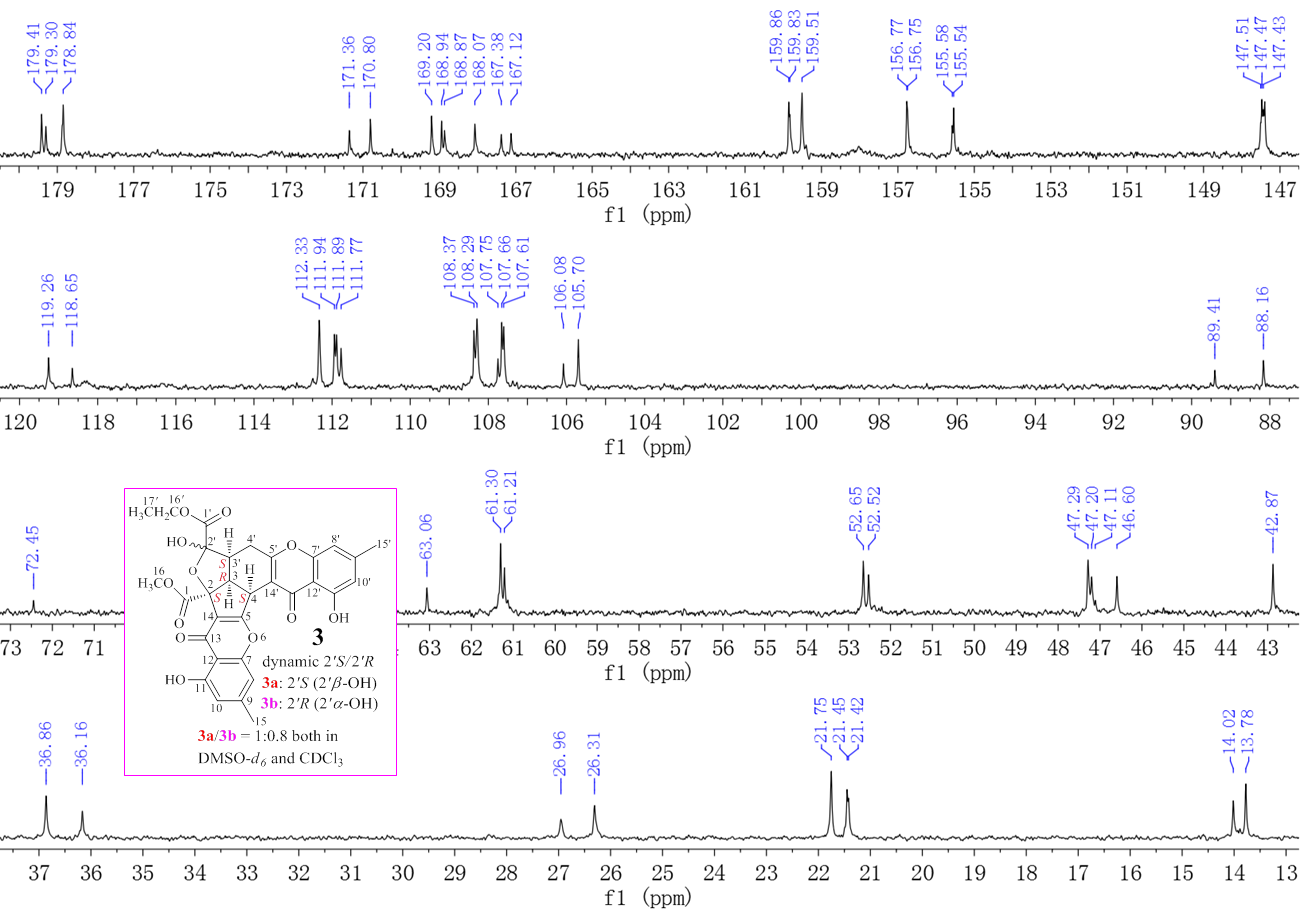


**Figure S33.** Expanded 150 MHz ^13^C NMR spectrum of **3** in DMSO-*d*_6_.


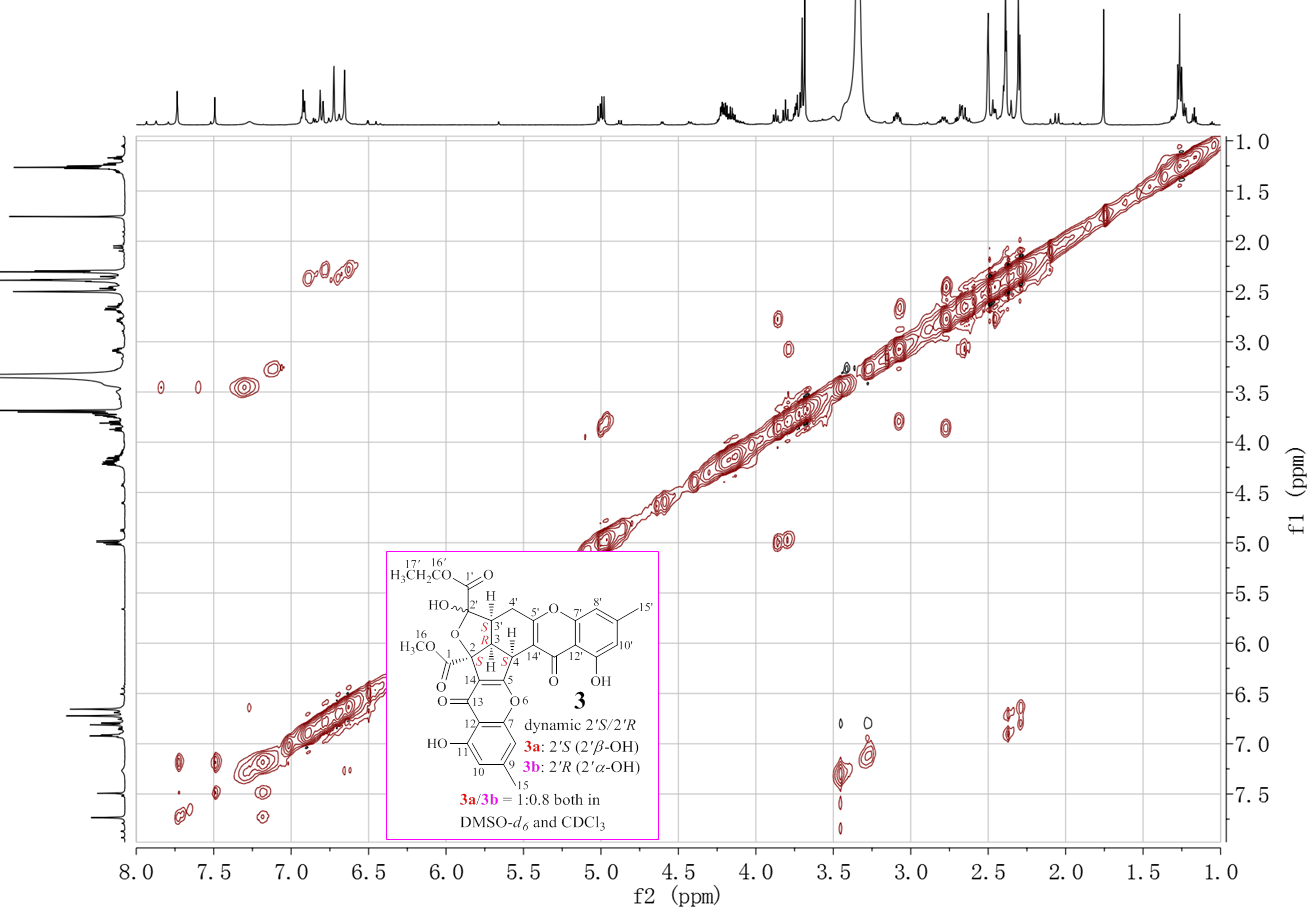


**Figure S34.** 600 MHz NOESY spectrum of **3** in DMSO-*d*_6_.


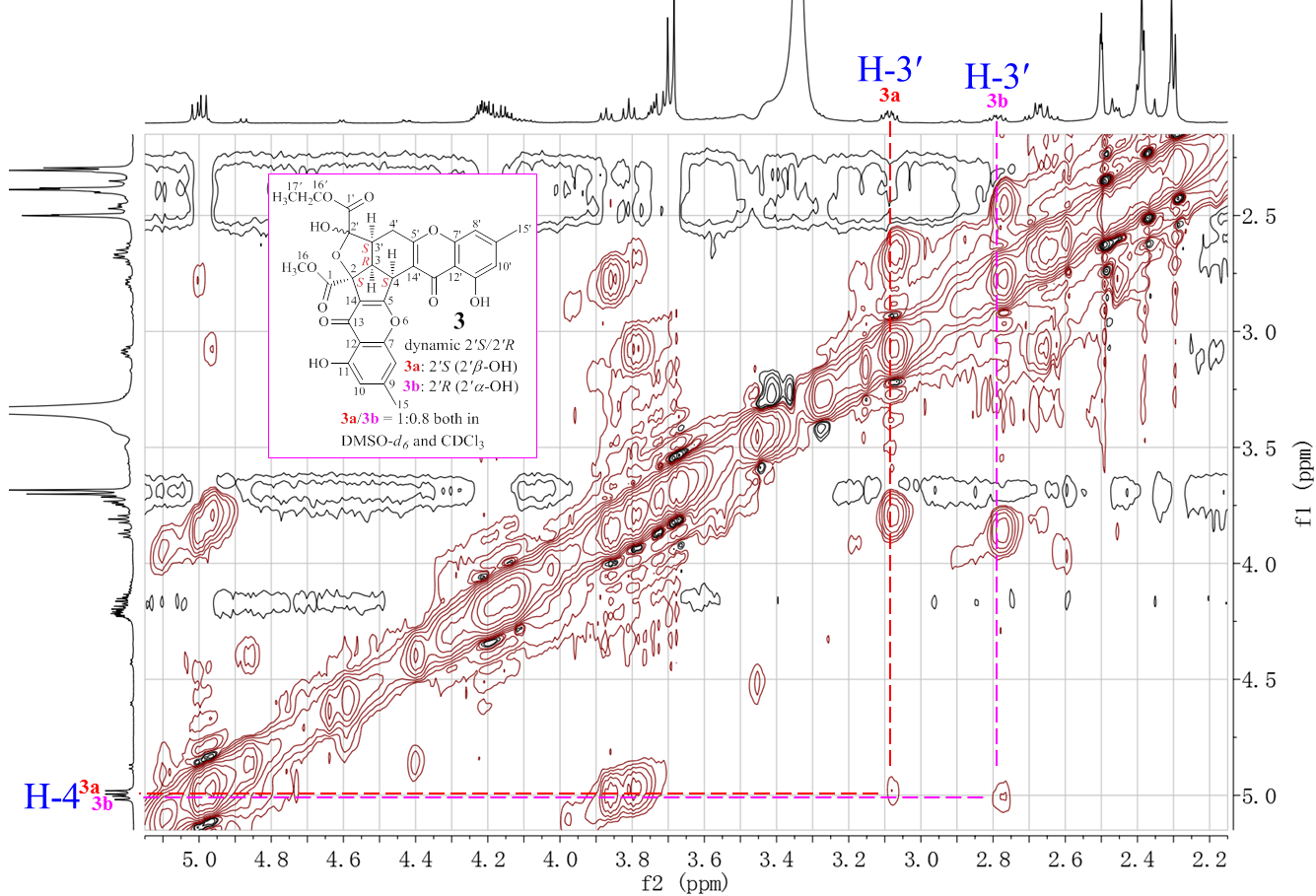


**Figure S35.** Expanded 600 MHz NOESY spectrum of **3** in DMSO-*d*_6_.


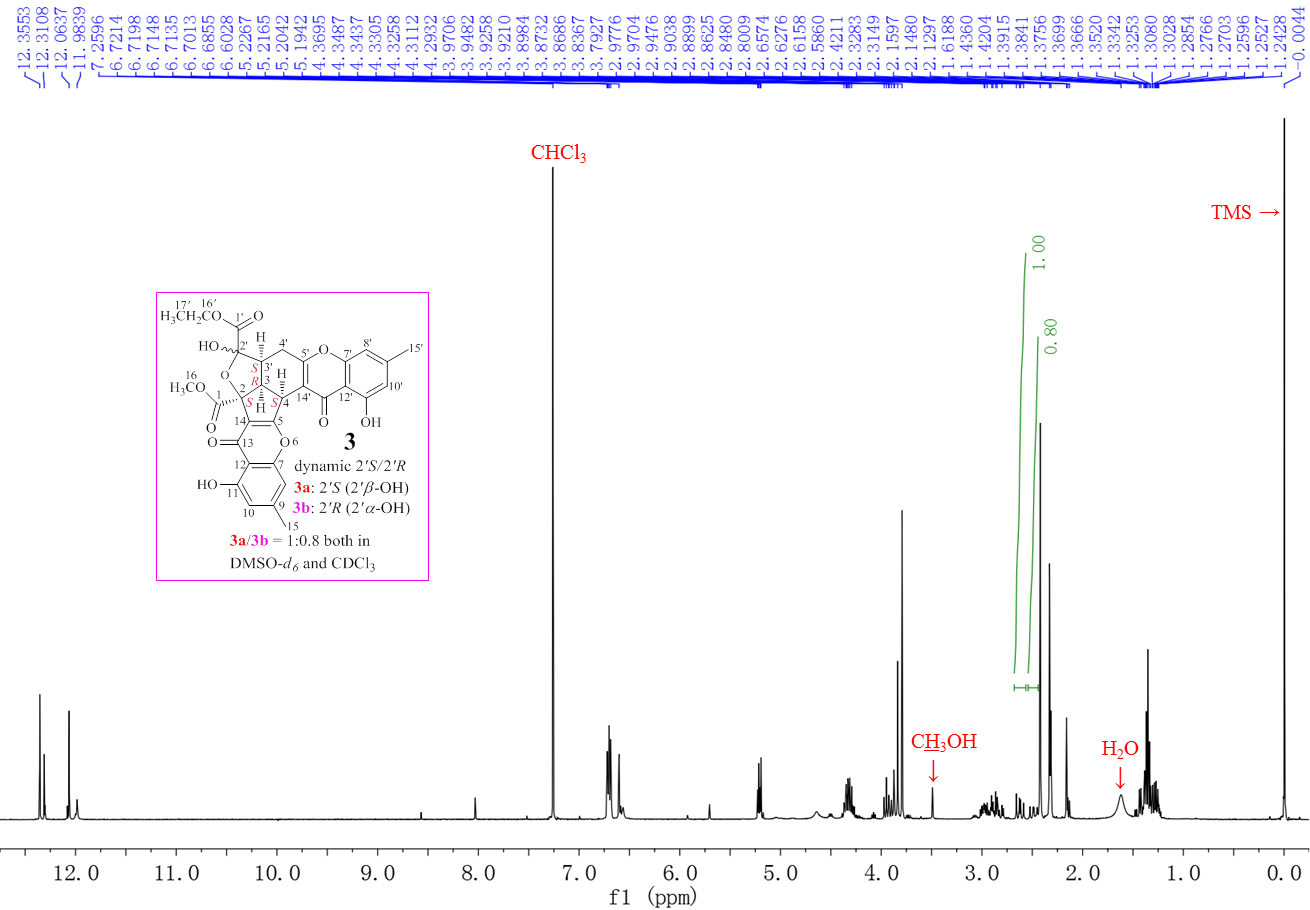


**Figure S36.** 400 MHz ^1^H NMR spectrum of **3** in CDCl_3_.


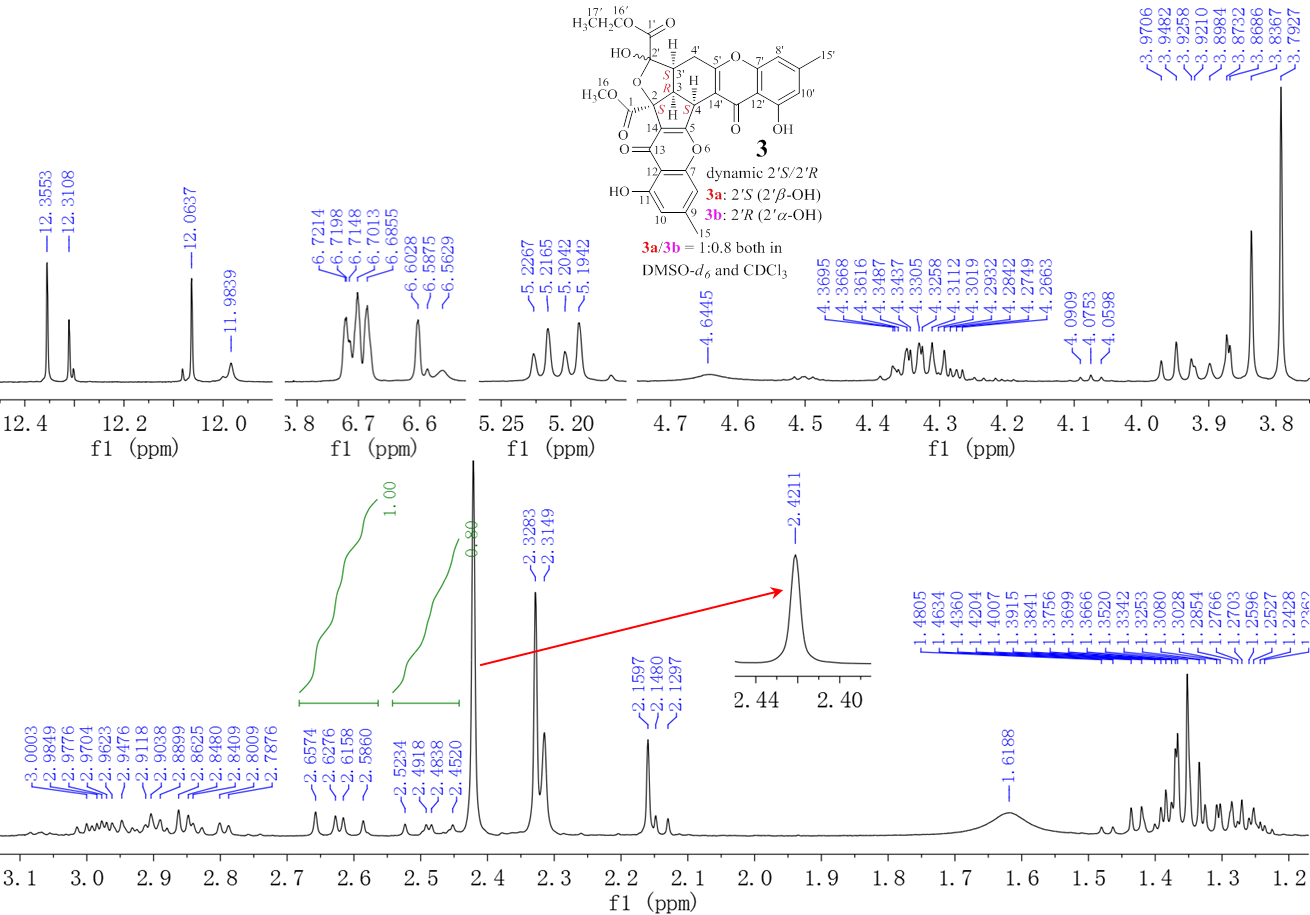


**Figure S37.** Expanded 400 MHz ^1^H NMR spectrum of **3** in CDCl_3_.


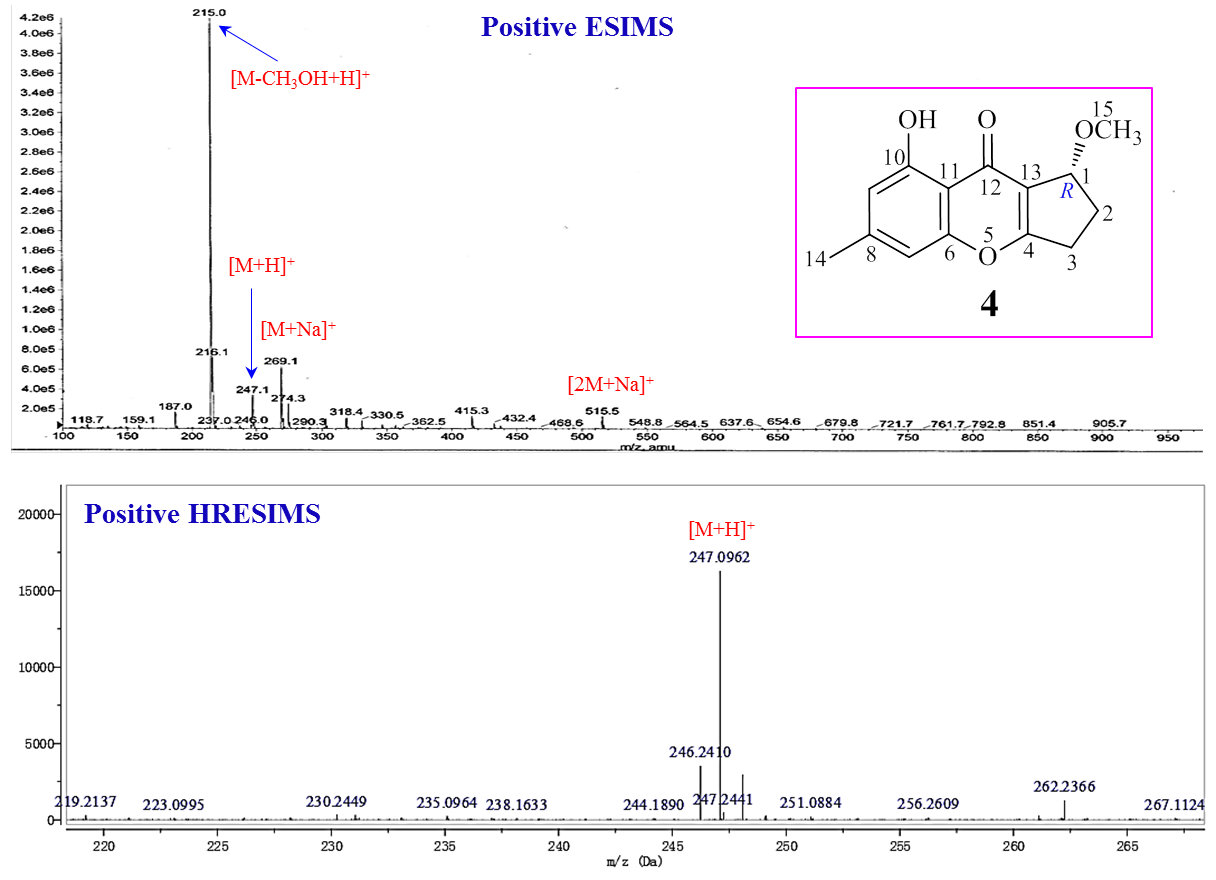


**Figure S38.** ESIMS and HRESIMS spectra of **4**.


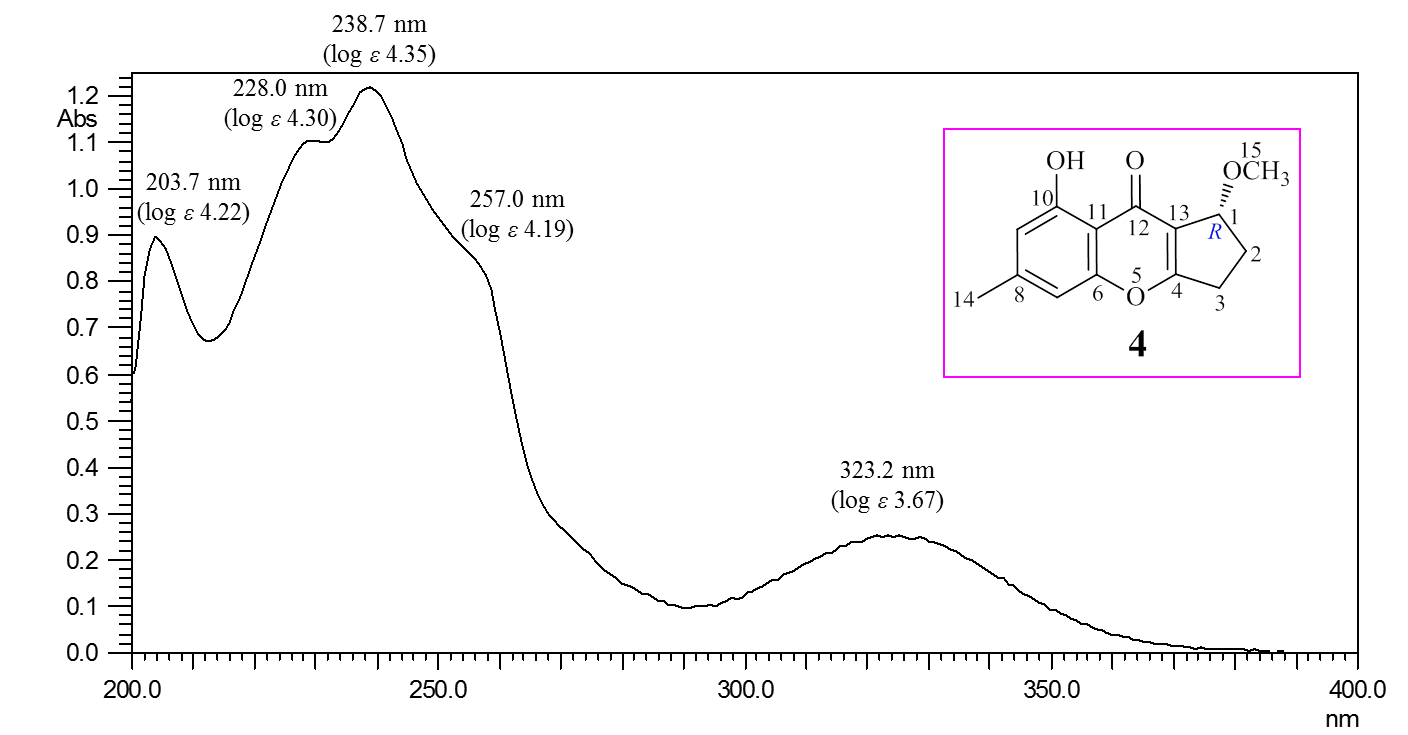


**Figure S39.** UV spectrum of **4** in MeOH.


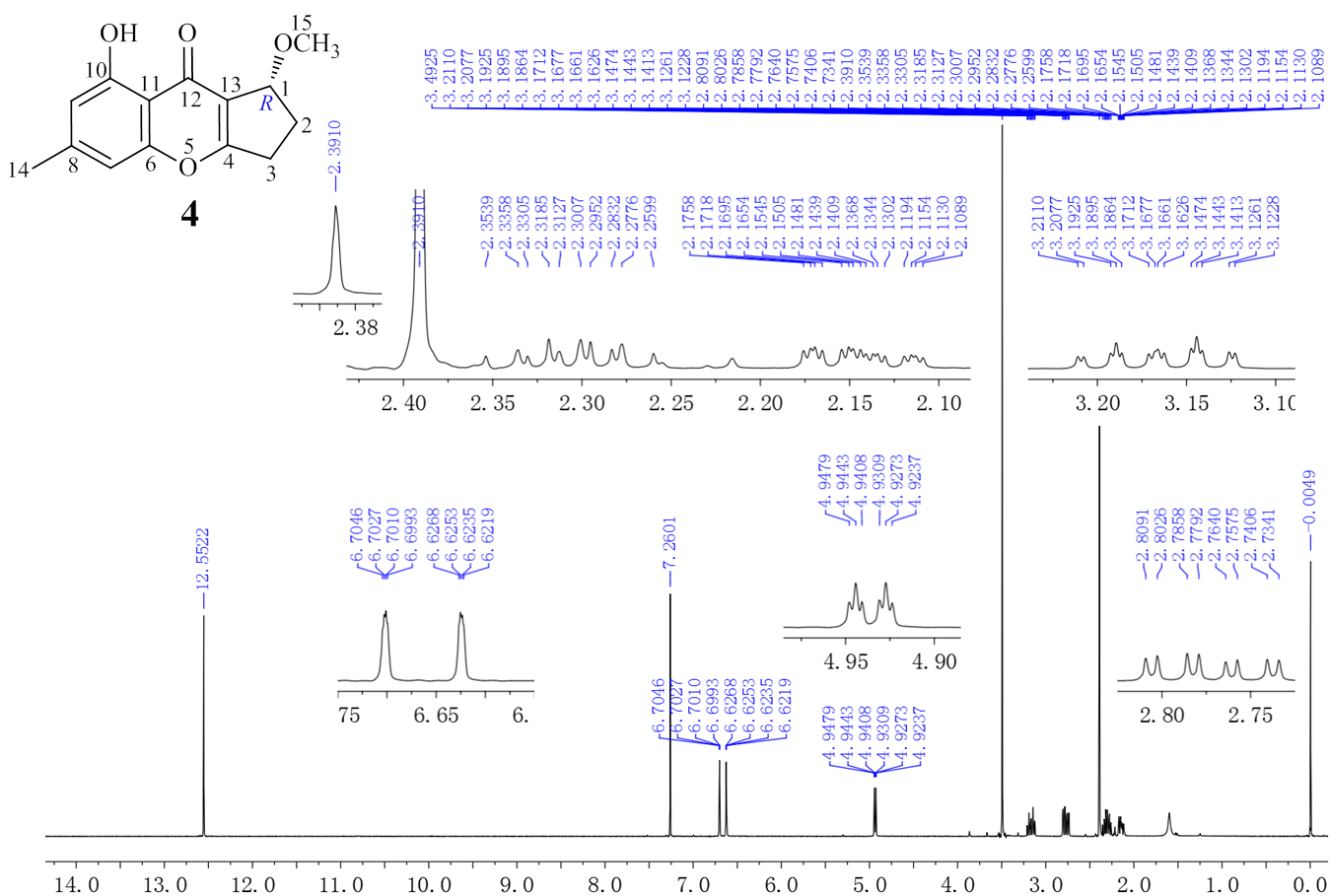


**Figure S40.** 400 MHz ^1^H NMR spectrum of **4** in CDCl_3_.


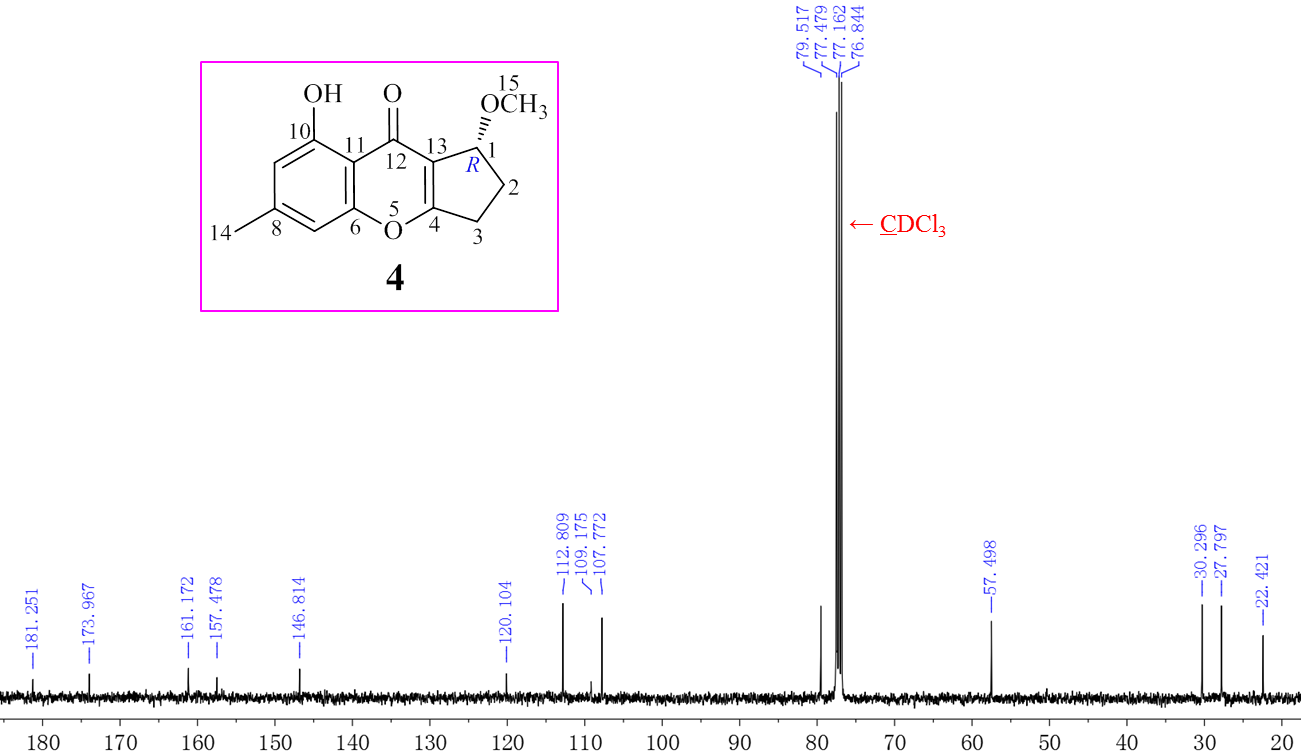


**Figure S41.** 100 MHz ^13^C NMR spectrum of **4** in CDCl_3_.
